# Dynamic principal modeling of cemented phosphogypsum stabilized soil under dry and wet cycles

**DOI:** 10.1371/journal.pone.0316643

**Published:** 2025-08-06

**Authors:** Zhangrong Ji, Kaisheng Chen, Kai Zhang

**Affiliations:** School of Civil Engineering, Guizhou University, Guiyang, Guizhou Province, China; Ural Federal University named after the first President of Russia B N Yeltsin: Ural'skij federal'nyj universitet imeni pervogo Prezidenta Rossii B N El'cina, RUSSIAN FEDERATION

## Abstract

Aiming at the influence of dynamic loading and wet/dry cycles during the operation of roadbed and in response to the proposal of Guizhou Provincial Government to promote the efficient utilization of phosphogypsum to solve the current situation of oversupply of phosphogypsum, the dynamic triaxial experiment was carried out to explore the dynamic constitutive model of phosphogypsum-stabilized soil with different numbers of wet/dry cycles, different peripheral pressures, and different consolidation ratios. The test results show that: (1) the effect of wet and dry cycles has a greater impact on the dynamic constitutive model of the mix, Monismith exponential model is suitable for the dynamic stress-strain curves in the case of wet and dry cycling; (2) The dynamic shear modulus-dynamic shear strain constitutive model of phosphogypsum stabilized soil was established based on dynamic soil mechanics, and the fitted correlation coefficients were all higher than 0.85, and the maximum values of MAE and RMSE were 4.827 and 5.990, respectively; (3) The prediction of the dynamic resilience modulus of phosphogypsum stabilized soil can be fitted by Ni model and power exponential model; (4) By comparing the dynamic and static modulus of rebound, it is concluded that the value of dynamic modulus of rebound for phosphogypsum-stabilized soil under dry and wet cycles is 1.1–1.5 times of the static modulus of rebound, and the recommended value of dynamic modulus of rebound is 90–100 MPa.

## 1 Introduction

Phosphogypsum is an industrial by-product of the production of phosphoric acid through wet chemical production of sulfuric acid and phosphate ores, and its main component is calcium sulfate dihydrate, and contains small amounts of silicon, fluoride, organic matter and radioactive elements. As every 1 ton of phosphoric acid produced in the production process is accompanied by 5 tons of phosphogypsum [1], it leads to a large accumulation of phosphogypsum. These accumulations not only encroach on valuable land resources, but also cause serious pollution to the atmosphere, soil and water environment due to long-term weathering and rain erosion, which in turn poses a potential threat to human health. On the other hand, the unique physical properties of red clay – high liquid limit, high plasticity limit, difficult to compact and easy to crack, which makes its application in the actual engineering is greatly restricted. Especially in the high-temperature and rainy climate of Guizhou, red clay undergoes frequent expansion and contraction cycles, which further weaken its physical and mechanical properties, and have a direct impact on the stability and safety of roads. Therefore, the direct application of red clay in road engineering faces great challenges.

The dynamic stress-strain relationship refers to the relationship between the dynamic stresses applied to a material and the dynamic strains it produces under dynamic loading loading conditions. This relationship is usually characterized by nonlinearity [2], while the dynamic shear modulus-dynamic shear strain relationship refers to the deformation and strength characteristics of soil under dynamic loading. Through this relationship, the change of shear modulus of soil body under different strain levels can be understood, and then the dynamic response and stability of soil body can be analyzed, which is not only of great significance for earthquake engineering and civil engineering, but also has a wide range of practical applications in the fields of geologic disaster prevention and control, urban construction and land development. And the prerequisite for the establishment of this relationship is the establishment of material dynamic principal relationship.

Based on hysteresis curve and backbone curve is one of the main means to construct the dynamic principal model, which is mainly to study the dynamic stress-dynamic strain relationship within the stress cycle by hysteresis curve, and to demonstrate the cumulative characteristics of strain by combining with backbone curve. The hysteresis curves are mainly studied by the viscoelastic medium method and Masing’s twofold method [3], while the backbone curves are mostly utilized by Hardin-Drnevich hyperbola, Ramberg-Osgood and other expressions [4–5]. These models are well applied but often have some limitations. Dynamic resilience modulus is a function that is related to the solubility index, the water content of the soil and the dry density of the soil under dynamic loading. For roadbed materials, dynamic resilience modulus can reflect its mechanical response under traveling load, and can be used to detect the strength and uniformity of pavement materials in road construction. Therefore, it is necessary to study the dynamic resilience modulus of the mix. Currently, the more commonly used models are power index model, Uzan model, NCHRP 1-28A model, etc. [6–10].

As far as the above is concerned, nowadays the study of dynamic principal structure of soil has been developed more maturely. Di Dai et al [11] elucidated the calculation method of asymmetric hysteresis loop by solidified undrained cyclic triaxial shear test. Based on the variation characteristics of the dynamic elastic modulus and damping ratio, empirical formulas were given for the fast estimation of the design.Duan Shuqian et al [12] proved the validity of the Duncan-Chang model for calculating the unloaded rebound modulus.Zhang Pei [13] et al, based on the hyperbolic function, put forward the concept of the normalized shear displacement, and deduced the normal stress in the shear process, the nonlinear Basic model of the effect of shear modulus and friction angle on shear strength. Xie Li et al [14] established a modified Iwan model for red clay soils in Gannan based on the Lwan model, considering the long-term accumulation of strain in red clay soils subjected to dynamic loading. Wang Jiaquan [15] et al. carried out dynamic triaxial tests to investigate the dynamic properties of reinforced gravelly soils, and obtained the equations of the backbone curves of reinforced gravelly soils under different influencing factors. Yin Pingbao et al [16] established the dynamic stress-dynamic strain fitting equation and dynamic elastic modulus decay model for nickel-iron slag-clay using hyperbolic model and negative power law function. Li Dongxue [17] and others used triaxial tests to explore the development of dynamic resilient modulus of clayey roadbed soils under the action of dry and wet cycles. As for the study of phosphogypsum, Li Zhangfeng et al [18] used phosphogypsum improved soil to conduct unconfined compressive test to get the ratios to meet the requirements of the roadbed strength specification.Millena Vasconcelos Silva et al [19] mixed and used semi-aqueous phosphogypsum with fine laterite and cement, and through dynamic triaxial test as well as microscopic test, etc., investigated the dynamic characteristics of the mixture and the microscopic development mechanism. Huang Wendong [20] and others established a new empirical dynamic constitutive model to study the dynamic properties of phosphogypsum stabilized soil and verified that the dynamic long-term modulus of resilience of the mix meets the requirements of the relevant specifications.

Based on the above studies, it can be seen that, regarding the dynamic constitutive modeling of soils, few studies have introduced the actual environmental impacts into the constitutive modeling. Most of the studies on phosphogypsum stabilized soils are focused on general physical properties or hydrostatic studies, and few studies on dynamic properties of phosphogypsum stabilized red clay are known. Therefore, in order to study the dynamic principal model of phosphogypsum-stabilized soil more comprehensively, this paper carries out dynamic triaxial experiments using cemented phosphogypsum as stabilizer and red clay as stabilized object, and incorporates the effect of dry and wet cycles in the test process. By fitting the experimental data to explore the dynamic stress-dynamic strain, dynamic shear modulus-dynamic strain constitutive models under different numbers of wet and dry cycles, different enclosing pressures, and different consolidation ratios and evaluating the accuracy of the models based on the MAE and RMSE error analyses, and then fitting the fitting parameters further to propose a modified dynamic constitutive model to overcome the lack of the study on the effect of wet and dry cycles in the previous studies, and finally, by orthogonal test Sensitivity analysis was added to understand the effect of variable changes on the model. For the study of the dynamic rebound modulus relationship curve is based on the Ni model [21] and the power index model, through the fitting data to explore the effect of different perimeter pressures, different bias stresses, and different numbers of wet and dry cycles on the dynamic rebound modulus and assess the accuracy of the model according to the MAE, RMSE error analysis, and the same increase in the sensitivity analysis to understand the effect of variable changes on the model.

## 2 Raw materials and test methods

### 2.1 Raw materials

#### 2.1.1 Phosphogypsum.

Phosphogypsum is mainly originated from the urnfu phosphorus mine area in Qiannan Buyi and Miao Autonomous Prefecture, Guizhou Province, and its appearance is characterized by a grayish-white surface with an irritating odor. Given that it contains potentially harmful impurities such as phosphorus and fluorine, which may cause adverse effects on the surrounding environment when used as a roadbed filler material, this experiment not only comprehensively detected the basic physical properties and chemical composition of phosphogypsum, but also added the determination of heavy metal content and radioactivity detection. The test results are shown in Tables 1–3.

**Table 1:** Physical mechanics of phosphogypsum and chemical composition test.

Testing Indicators	retrieve a value	Chemical composition	mass fraction/%
Fineness/(%)	43.5	SO3	49.07
$\rho /(g/{cm}^{3})$	2.26	CaO	40.07
SSA/(m2/kg)	101	SiO2	5.78
WS/(%)	5.4	F	1.89
LOI/(%)	18.42	P2O5	1.35
Alkali content/(%)	1.30	Na2O	0.587
Mass fraction of sulfur trioxide/(%)	0.07	Al2O3	0.435
		Fe2O3	0.210

**Table 2:** Phosphogypsum radioactivity test results.

Testing Program	CRa /(Bq·kg-1)	CTh /(Bq·kg-1)	Ck /(Bq·kg-1)	Internal irradiance index(IRa)	External exposure index(Ir)
Technical Requirements				≤1.0	≤1.0
Test results	56.26	3.84	26.65	0.3	0.2
Individual judgment				Eligible (voter etc)	Eligible (voter etc)

**Table 3:** Phosphogypsum heavy metal content test results.

Test element	Constant volume V_0_/mL	Test Solution Element Concentration C_0_/ug·L^-1^	Dilution factor /f	Elemental concentration C_1_/mg·L^-1^	Elemental content C_x_/mg·kg^-1^	Testing Conclusion
(Cd)	10	0.0620	50	3.10	0.26	Meets the requirements of national standards
(Pb)	10	1.5270	50	76.35	6.33	
(Cr)	10	0.2920	50	14.60	0.96	
(As)	10	0.1284	50	6.42	0.42	
(Hg)	10	0.0484	50	2.42	0.16	

#### 2.1.2 Red clay.

The soil used in this test is mainly from Fuquan City, Guizhou Province, cattle field to Daoping highway reconstruction and expansion project along the line, the depth of the soil sampling 0-3m, the surface of the soil samples is brownish-yellow, the soil quality is more uniform accompanied by a small amount of gravel, the structure is dense, the natural water content is high, cohesion, which is consistent with the characteristics of a typical red clay. After retrieval, the basic physical properties and chemical composition were tested first, as shown in Table 4, from which it can be seen that its main chemical composition is SiO2, and has high liquid limit, high plastic limit and other undesirable engineering properties.

**Table 4:** Basic physical indexes and chemical composition of red clay.

Physical index	Retrieve a value	Chemical composition	Mass fraction/%
ω/%	57.88	SiO_2_	54.16
$W_{P}/\;\%$	52.13	Al_2_O_3_	28.70
$W_{L}/\;\%$	74.65	Fe_2_O_3_	10.36
plasticity index	22.52	Si	26.90
$\omega_{op}/\;\%$	32.12	Al	15.90
$\rho_{dmax}/g\cdot {cm}^{-3}$	1.482	Fe	7.94
		else	3.61

#### 2.1.3 Cement.

The cement was purchased from PO42.5 ordinary silica sodium salt cement, gray and dry, produced by Guizhou Senyao Cement Co. The basic parameters are shown in Table 5.

**Table 5:** Basic cement parameters.

Heat loss /%	SO_3_ /%	Alkali content /%	Incipient condensation time /min	Time of final coagulation /min	Stability
1.58	2.87	2.42	302	322	Eligible
Cl^-^/%	Gypsum content /%	3-Day Flexural Strength /Pa	28-Day Flexural Strength /MPa	3-day compressive strength /MPa	28-day compressive strength /MPa
0.018	5.00	5.0	6.7	24.9	43.7

### 2.2 Test method

#### 2.2.1 Test apparatus.

**Fig 1:**
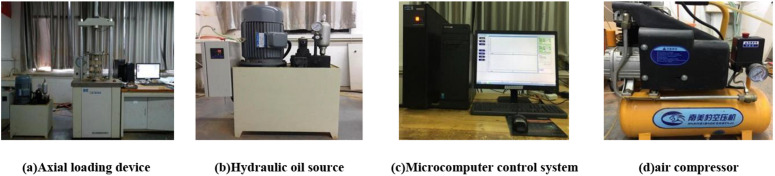
Dynamic triaxial testing machine.

This test mainly adopts SDT-20 dynamic three-axis testing machine as shown in Fig 1. The device is mainly composed of axial loading system, hydraulic oil source, microcomputer control system, air compressor and other parts.

#### 2.2.2 Sample preparation.

At present, there have been a lot of studies at home and abroad on phosphogypsum stabilized soil used as road base material, determined by the recommended reagent dosage of cement stabilized material in the Technical Rules for Construction of Highway Pavement Base Levels (JTG/T F20-2015). For subgrade, the recommended dosage of cement is 6%−14% for roadbase where the stabilized material is soil and the plasticity index is greater than 12. Chen J et al [22] concluded that the fracture rate decreases with the increase of cement as well as phosphogypsum dosage through the fracture test of phosphogypsum stabilized soil, which indicates that the increase of cement dosage can alleviate the generation of fracture. Kun Zhang et al [23] proposed the optimum ratio of phosphogypsum stabilized soil by orthogonal test using polar analysis: cement 7%, phosphogypsum: red clay = 1:2. Peng Bo and Zhou Mingkai et al [24–25] concluded that the cement dosage of phosphogypsum stabilized soil is 4%−6% by indoor test. Therefore, in order to study the dynamic constitutive relationship between phosphogypsum and red clay mixtures in more depth and to positively effect the Guizhou Provincial Government’s proposal to promote the efficient utilization of phosphogypsum, this paper, on the basis of the above mentioned, adopts the cement contents of 4%, 6% and 8%, and extends the ratios of phosphogypsum and red clay (1:0.5, 1:1, 1:2, 1:3, 1:4, 1:5) to prepare specimens for the 18 ratios for the dynamic triaxial test. Among them, 9 ratios of phosphogypsum to red clay in the ratios of 1:0.5, 1:1, 1:2 with 4%, 6% and 8% cement content were selected for the dynamic rebound modulus test.

**Fig 2:**
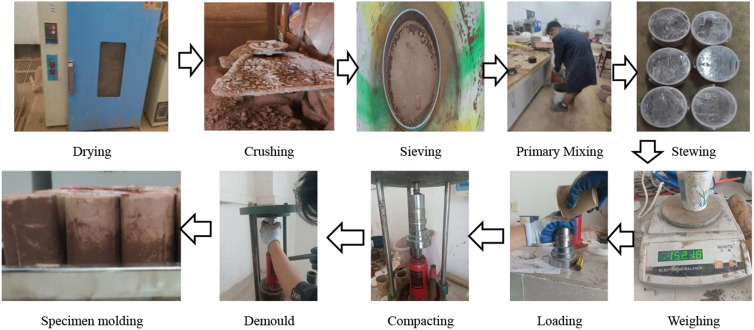
Flow of specimen preparation.

The specimen preparation process is shown in Fig 2, phosphogypsum and red clay were dried, crushed and sieved, weighed and mixed according to the predetermined ratio. Based on the optimal moisture content, the required amount of water was calculated and added, and the material was closed and simmered for 24 hours after mixing. Cement and remaining water were then added for a second mixing and sieved again. Finally, the specimens were molded by hydrostatic compression and placed in a curing box for subsequent testing.

#### 2.2.3 Pilot program.

(1) Dynamic triaxial test program

Sine wave loading can simulate the vibration effect of the train on the roadbed, so for the dynamic stress-strain test this paper chooses gradually increasing sine wave loading, the number of vibrations of each level is taken 10 times [26], the load is taken as 10kN. and because the train load mainly produces low frequency effect, the frequency is affected by the model, the speed of the car, etc., so in the consideration of the model and the grade of the highway, the frequency is taken as 2 Hz, as shown in Table 6. The dynamic rebound modulus program is based on the “Highway Geotechnical Testing Regulations” [27] fine-grained soil specimen loading sequence followed by the standard values shown in Table 7, first of all, the specimen is applied to the specimen 30KPa preloaded perimeter pressure, and the specimen is applied to the specimen at least 1,000 times of the semi-formal impulse loading at a frequency of 10 Hz, the loading time of 0.1s intermittent 0.9s. Then adjust the perimeter pressure and the semiformal impulse loading to the target setting value, and then repeat loading 100 times with 10 Hz frequency, then adjusting the perimeter pressure and semi-formal impulse loading to the target setting value, and repeat loading with 10 Hz frequency. Then adjust the circumferential pressure and semi-formal impulse load to the target setting value and repeat the loading 100 times at a frequency of 10 Hz with a loading time of 0.1s and an interval of 0.9s.

**Table 6:** Dynamic strain and dynamic strain test program.

Frequency/(HZ)	Pressurization/(kPa)	Consolidation ratio	Number of cycles N/times
2	40	1	0, 1, 2, 3, 4, 5
		1.5	0, 1, 2, 3, 4, 5
		2	0, 1, 2, 3, 4, 5
2	80	1	0, 1, 2, 3, 4, 5
		1.5	0, 1, 2, 3, 4, 5
		2	0, 1, 2, 3, 4, 5
2	120	1	0, 1, 2, 3, 4, 5
		1.5	0, 1, 2, 3, 4, 5
		2	0, 1, 2, 3, 4, 5

**Table 7:** Loading sequence of fine-grained soil specimens.

Load Serial Number	peripheral compressive stress σ_3_(Kpa)	contact stress σ_c_(Kpa)	cyclic stress σ_d_(Kpa)	axial stress σ_max_(Kpa)	Number of Load Actions
0	30	6	55	61	1000
1	60	12	30	42	100
2	45	9	30	39	100
3	30	6	30	36	100
4	15	3	30	33	100
5	60	12	55	67	100
6	45	9	55	64	100
7	30	6	55	61	100
8	15	3	55	58	100
9	60	12	75	87	100
10	45	9	75	84	100
11	30	6	75	81	100
12	15	3	75	78	100
13	60	12	105	117	100
14	45	9	105	117	100
15	30	6	105	111	100
16	15	3	105	108	100

(2) Wet-dry cycling test

**Fig 3:**
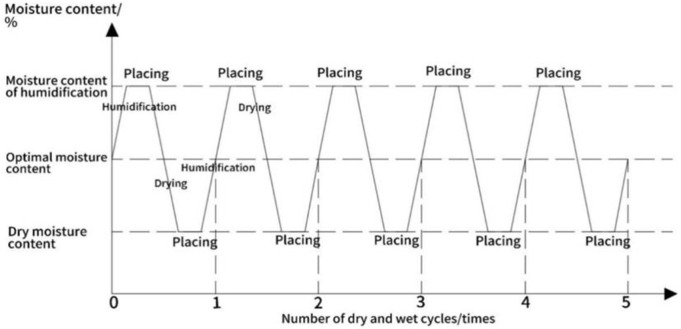
Dry and wet cycle test methods.

Based on the research of scholars such as Tang Yunli and Chen Kaisheng [28–29], we found that the dry-wet cycle of wetting the soil body first and then drying it leads to a more significant strength attenuation compared to the process of drying it first and then wetting it. Considering the most unfavorable conditions in engineering practice, in this paper, we decided to use the wet-first-dry-wet-dry cycling scheme to test the specimens. In addition, the studies of scholars such as Que Yun, Hu Zhi, and Li Peile [30–32] showed that under the influence of natural climate, the moisture content of road base shows periodic or non-periodic fluctuations around the “equilibrium moisture content” (EMC), and the range of fluctuations is roughly EMC ± 5%. Combined with the research results of Zhou Hao, Chen Kaisheng and other scholars [33,34], that is, the strength of the soil body tends to stabilize after 5 ~ 6 times of wet and dry cycles, this paper formulates a wet and dry cycle scheme with 5 times of wet and dry cycles and 10% wet and dry amplitude, in order to simulate and analyze the change of the strength of the soil body under the action of wet and dry cycles [35]. As shown in Fig 3: first, the specimens were placed on permeable stones, closed in an acrylic box, and the humidity in the box was increased by a humidifier until the specimens reached a predetermined weight, after which the humidifier was turned off and left to stand for 24 hours. Subsequently, the specimens were placed in an oven at a set temperature of 40°C for drying, and were continuously weighed until a predetermined dry weight was reached, and then left to dry again for 24 hours to complete a cycle. This process was repeated until five wet and dry cycles were completed.

## 3 Result analysis

### 3.1 Dynamic stress-dynamic stress variability intrinsic modeling of mixes

(1) The effect of surrounding pressure

**Fig 4:**
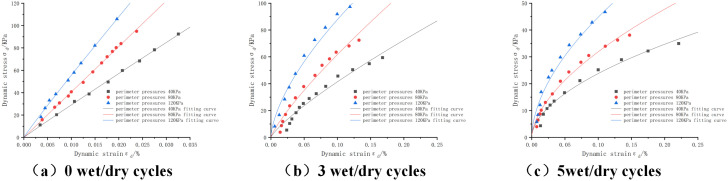
Dynamic stress-strain curves of cement:phosphogypsum:red clay = 6:47:47 mixes with different enclosure pressures, consolidation ratio 1.5.

The dynamic stress-strain curves of the mixture under different pressures are shown in Fig 4: Since the laws of other ratios are similar, the other ratios are not shown anymore. From the figure, we can see that, before the wet and dry cycles, the dynamic stress and the dynamic strain under the pressures of 40Kpa, 80Kpa and 120Kpa are positively correlated, and with the increase of the peripheral pressure, the slope of the dynamic stress-strain curves also increases. This is mainly due to the fact that the soil body is less affected by the external environment and is in the elastic deformation stage before the wet and dry cycles. When in the third cycle, we found that the curve gradually transitioned from linear elasticity to nonlinear curve, and with the increase of the number of wet and dry cycles, the tendency of the curve to slow down is more obvious [36]. From the longitudinal point of view, with the increase of the perimeter pressure under the same strain, the dynamic stress also increases, so it can be seen that the perimeter pressure has a promoting effect on the increase of the dynamic stress, which is mainly due to the fact that when the perimeter pressure increases, the force between the material particles also increases gradually and thus becomes closer, and the friction also increases, making it more and more difficult to dislocate each other in the soil, which ultimately results in the strength of the soil body is also increased.

In order to investigate the dynamic stress-dynamic stress variation constitutive model applicable to phosphogypsum stabilized soil under wet and dry cycles, different dynamic constitutive equations were fitted to the dynamic triaxial test data in this paper, and the minimum values of the fitted correlation coefficients under different numbers of wet and dry cycles are shown in Table 8, from which it can be seen that: the exponential model fit >Bingham’s model> Hooke’s model. therefore, the Monismith [37] exponential function is mainly used for data fitting in the following paper, and the root mean square error (RMSE) and the mean absolute error (MAE) of the model were calculated as an evaluation index of the model’s accuracy after the fitting. The fitting results, MAE and RMSE are shown in Table 9, from which it can be seen that the dynamic stress-dynamic strain curves of 0,3 and 5 wet and dry cycles under different perimeter pressures can be fitted with Monismith exponential function [38], and the results of the fitting are all greater than 0.96, the maximum value of the MAE is 4.2009, and the maximum value of the RMSE is 4.7936, which indicates that the fitting situation is good, small error can be expressed using this function.

**Table 8:** Dynamic stress-dynamic strain fitting parameters for different constitutive equations.

Fitting equation	Number of dry and wet cycles/N	Perimeter pressures/KPa	R^2^
Monismith exponential: $\sigma_{d}=a*\epsilon_{d}^{b}\backslash )$	0	120	0.997
	3	40	0.961
	5	80	0.941
Hooke model [39]: $\sigma_{d}=c\varepsilon_{d}$	0	120	0.995
	3	120	0.832
	5	40	0.646
Bingham model [40]: $\sigma_{d}=\sigma_{0}+c\varepsilon_{d}$	0	120	0.997
	3	120	0.921
	5	80	0.891

**Table 9:** Dynamic stress-dynamic strain fitting parameters under different dry and wet cycles and different perimeter pressures and results of the evaluation of the errors with the models.

Fitting equation	Number of dry and wet cycles/N	Perimeter pressures/KPa	R^2^	MAE	RMSE
$\sigma_{d}=a*\epsilon_{d}^{b}\backslash )$	0	40	0.999	0.7577	0.8475
		80	0.998	0.7613	0.9275
		120	0.997	0.9423	1.3234
	3	40	0.961	4.2009	4.7936
		80	0.972	3.7517	4.5338
		120	0.977	2.9019	3.5273
	5	40	0.980	0.9574	1.3643
		80	0.977	1.6617	1.9199
		120	0.982	1.7910	2.1433

Note: where is the dynamic stress;$\varepsilon_{d}$ is the dynamic strain.

(2) Effect of consolidation ratio

**Fig 5:**
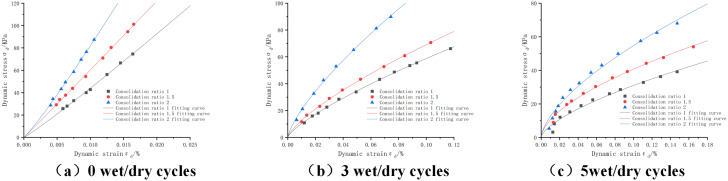
Dynamic stress-strain curves of cement:phosphogypsum:red clay = 6:47:47 mixes with different consolidation ratios, perimeter pressure 80kPa.

The dynamic stress-strain curves of the mixes with different consolidation ratios are shown in Fig 5: due to the space limitation, only the dynamic stress-strain curves with cement:phosphogypsum:red clay = 6:47:47 and 80kPa are shown. It can be seen from the figure that the dynamic stress-strain curves of the three consolidation ratios are positively correlated before the wet and dry cycles. With the increase of the number of wet and dry cycles, the dynamic stress-strain curves gradually show a nonlinear relationship due to the increase of the external disturbance of the mixture. In the longitudinal direction, the slope of the dynamic stress-strain curve increases with the increase of consolidation ratio. This is mainly due to the fact that with the increase of consolidation ratio, the soil body is more compact with each other, which enhances the interaction between particles.

The fitted parameters and model evaluation results of dynamic stress-dynamic strain curves of mixes with different consolidation ratios are shown in Table 10, through the data in the table, we can see that the fitted results of R^2^ are greater than 0.97, the maximum value of MAE is 1.7853, and the maximum value of RMSE is 2.3315, which indicates that the fitting situation is good.

**Table 10:** Fitting parameters of dynamic stress-strain curves of mixes with different consolidation ratios and results of evaluation of the error with modeling.

Fitting equation	Number of dry and wet cycles/N	Consolidation ratio	R^2^	MAE	RMSE
$\sigma_{d}=a*\epsilon_{d}^{b}\backslash )$	0	1	0.998	0.5953	0.7347
		1.5	0.998	0.7352	0.9432
		2	0.998	0.5386	0.7608
	3	1	0.999	0.4016	0.4962
		1.5	0.999	0.4082	0.4904
		2	0.998	0.7261	0.9525
	5	1	0.978	1.1121	1.6458
		1.5	0.993	0.7495	1.1406
		2	0.986	1.7853	2.3315

Note: where $\sigma_{d}$ is the dynamic stress;$\varepsilon_{d}$ is the dynamic strain.

(3) Effect of the number of wet and dry cycles

**Fig 6:**
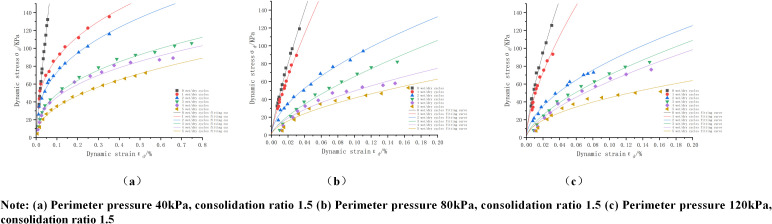
Dynamic stress-strain curve of cement:phosphogypsum:red clay = 6:47:47 mix with different number of dry and wet cycles.

The dynamic stress-strain curves of the mixes with consolidation ratio of 1.5 and C:P:T = 6:47:47 are shown in Fig 6, and only those with consolidation ratio of 1.5 are shown due to the similarity of the curves for other consolidation ratios. From the figure, it can be seen that the dynamic stress-strain is linear at 0 dry and wet cycles, and the curve gradually slows down at the beginning of the dry and wet cycles, and with the increase of the number of dry and wet cycles the curve slows down more and more, from the analysis of the internal mechanism, it can be seen that, when mixing the cement and the phosphogypsum, it will produce hydration reaction and generate calcium alumina to play the role of the skeleton support, which is the source of the strength of the mix in the early stage. When the wet and dry cycles start the curve from steep to slow indicates that the hardening becomes soft, the soil body internal damage in the wet and dry cycles, the soil body curve of the degree of moderation can reflect the size of the internal damage. Further calculations in Fig 6(a) show that the dynamic stress of the first wet-dry cycle is attenuated by 7.5% at the same strain. When it reaches the fifth time, the attenuation is nearly 85%, so it can be seen that the influence of wet and dry cycles on the internal soil body is very serious. From the analysis of the external environment, it can be seen that this may be due to the uneven heating in the process of wet and dry cycles led to the generation of tensile stresses, resulting in cracks, when increasing the number of cycles will lead to this unfavorable phenomenon continues to deepen thus affecting the strength of the soil body size.

The fitted parameters of the dynamic stress-dynamic strain curves of the mixes with different number of wet and dry cycles are shown in Table 11, and the results of the model evaluation are shown in Table 12. From the data in the table, we can see that the fitted results of the R^2^ are greater than 0.92, the maximum value of the MAE is 4.0065, and the maximum value of the RMSE is 4.5077, which indicates that the fitting is good. However, the fitting equations do not reflect the effect of the number of wet and dry cycles, so this paper will be in Table 12, the values of the parameters a, b with different wet and dry cycle times for further fitting. Through the fitting analysis, the relationship equation between parameters a, b and the number of wet and dry cycles can be established as shown in Table 13, and the relationship equation can be substituted into the Monismith exponential model, then a new modified constitutive model can be obtained as shown in Table 14, from which we can see that the R^2^ is greater than 0.80, and therefore the modified constitutive model can provide a reference for the study of the dynamic properties of the stabilized soil with phosphogypsum.

**Table 11:** Fitting parameters of dynamic stress-strain curves of mixes under different dry and wet cycles.

Fitting equation	Peripheral pressure /KPa	Number of dry and wet cycles/N	R^2^	Fitting equation	Peripheral pressure /KPa	Number of dry and wet cycles/N	R^2^
$\sigma_{d}=a*\epsilon_{d}^{b}\backslash )$	40	0	0.996	$\sigma_{d}=a*\epsilon_{d}^{b}\backslash )$	80	3	0.979
		1	0.982			4	0.935
		2	0.989			5	0.940
		3	0.991	$\sigma_{d}=a*\epsilon_{d}^{b}\backslash )$	120	0	0.989
		4	0.976			1	0.971
		5	0.991			2	0.990
$\sigma_{d}=a*\epsilon_{d}^{b}\backslash )$	80	0	0.996			3	0.980
		1	0.993			4	0.972
		2	0.991			5	0.924

Note: where $\sigma_{d}$ is the dynamic stress;$\varepsilon_{d}$ is the dynamic strain.

**Table 12:** Error evaluation results of dynamic stress-strain model for mixes under different dry and wet cycles.

Number of dry and wet cycles/N Peripheral pressure/KPa	40		80		120	
	MAE	RMSE	MAE	RMSE	MAE	RMSE
0	1.1886	1.4828	1.5649	1.7827	2.2551	2.9554
1	2.9719	3.9112	1.2313	1.5289	2.8563	3.4656
2	1.7403	2.4952	1.7593	2.2202	1.7507	1.9442
3	2.7948	3.2932	3.0594	3.5773	2.9098	3.4583
4	3.6036	4.1861	4.0065	4.5077	3.0684	3.7623
5	1.3359	1.9234	3.2552	3.7149	3.5237	4.1293

**Table 13:** Values of fitted parameters for dynamic stress-strain curves of mixes with different perimeter pressures and different numbers of wet and dry cycles.

Pressurization/KPa	Number of dry and wet cycles/N	a	b
40	0	1733.83	0.886
	1	196.82	0.345
	2	174.70	0.379
	3	122.94	0.404
	4	112.48	0.396
	5	98.64	0.461
80	0	1864.55	0.805
	1	1471.16	0.775
	2	335.64	0.586
	3	325.02	0.655
	4	195.74	0.597
	5	164.13	0.598
120	0	1515.70	0.709
	1	914.40	0.617
	2	305.60	0.573
	3	302.34	0.634
	4	278.83	0.647
	5	146.98	0.518

**Table 14:** Parameter a, b fitting relationship equation.

Pressurization/KPa		Parameter a relational equation	R^2^	Parameter b relational equation	R^2^
40	a = 1608.53*e^(-N/0.3275)+125.196		0.999	b = 0.426*e^(N/-0.036)+0.433	0.929
80	a = 2023.99*e^(-N/1.913)-66.746		0.918	b = 1.4478 + 0.852*sin(pi*(N + 22.82)/18.02)	0.800
120	a = 1404.48*e^(-N/1.358)+133.39		0.971	b = 0.715–0.205*N + 0.099*N^2^-0.013* N^3^	0.982

Note: N is the number of wet/dry cycles.

(4) Influence of mix ratio

**Fig 7:**
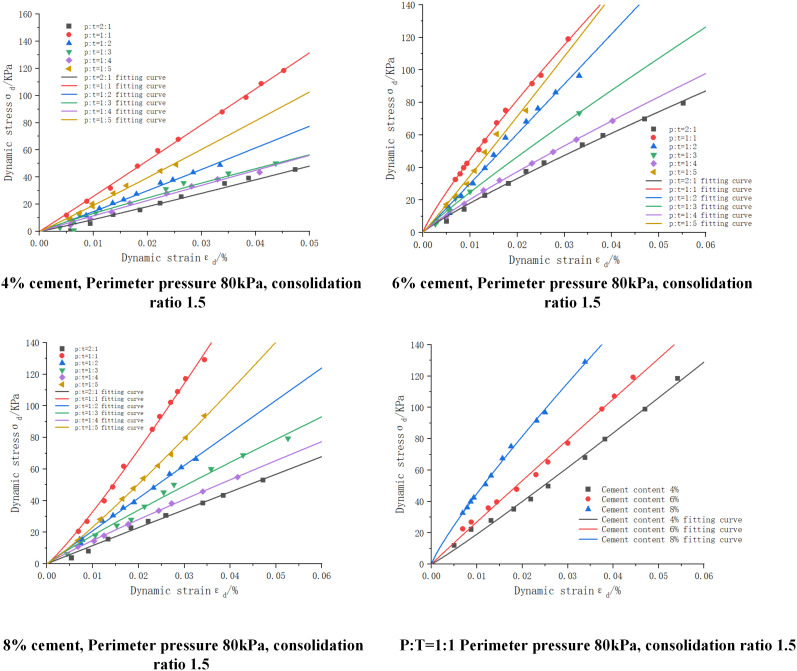
Dynamic stress-strain curves of mixes with different mix ratios under 0 dry and wet cycles.

As shown in Fig 7: due to space limitations, take the perimeter pressure of 80Kpa, consolidation ratio of 1.5, 0 times wet and dry cycles under different mix ratio dynamic stress-strain curve for analysis, from the figure can be seen: when the P:T = 1:1 when the dynamic stress-strain curve slope is the largest, from the internal response this is mainly due to the cement and phosphogypsum generated by the cementitious material is the most, play the most obvious role in supporting the most obvious. The soil body has the best compactness between them and the soil body has the greatest strength. By analyzing Fig 7(a), it can be seen that in the case of the same gypsum: red clay, increasing the cement dosage has the effect of improving the strength of the soil body, and the effect is more obvious with the increase of strain.

The fitted parameters of dynamic stress-strain curves of mixes with different ratios are shown in Table 16. Due to the limited space, only the minimum fitted correlation coefficients of each different cement dosage are shown in the table, and from the table, it can be found that the minimum value of R^2^ is 0.985 for 4% of the cement, 0.978 for 6% of the cement, and 0.985 for 8% of the cement. R^2^ is greater than 0.97, indicating that the fit is good and can be used to fit the curve with the fitting equations in the table.

**Table 15:** Modified eigenstructural model.

Pressurization/KPa	Modified eigenstructural model
40	σd=(1608.53*e^(−N/0.3275)+125.196)*ϵd0.426*e^(N/−0.036)+0.433\)
80	σd=(2023.99*e^(−N/1.913)−66.746)*ϵd1.4478+0.852*sin(pi*(N+22.82)/18.02)\)
120	σd=(1404.48*e^(−N/1.358)+133.39)*εd0.715−0.205*N+0.099*N2−0.013*N3

Note: N is the number of wet/dry cycles.

**Table 16:** Fitting parameters of dynamic stress-strain curves of lower mixes with different ratios.

Fitting equation	Cement content	Peripheral pressure /KPa Consolidation ratio	R^2^min
$\sigma_{d}=a*\epsilon_{d}^{b}\backslash )$	4%	80;1.5	0.985
	6%		0.978
	8%		0.985

Note: where $\sigma_{d}$ is the dynamic stress;$\varepsilon_{d}$ is the dynamic strain.

(5) Proposed Dynamic Stress-Dynamic Stress Variation Structural Model for Phosphogypsum Stabilized Soil

From the fitting images shown in Figs 4-7, the fitting parameters in Tables 9–16, and the MAE and RMSE models, it is concluded that the exponential model has a better fit and lower error for the dynamic stress-dynamic strain curves with different proportions, perimeter pressures, number of dry and wet cycles and consolidation ratios, and the exponential model Eq. (1) is applicable to the dynamic stress-strain curves under the conditions of dry and wet cycling, and The proposed new empirical dynamic constitutive model based on wet and dry cycles has a high degree of fit can provide a reference for the study of dynamic properties of mixes.


$$\sigma_{d}=a*\epsilon_{d}^{b}\mathrm{\backslash ]}$$ (1)

Note: $\sigma_{d}$ is the dynamic stress; $\varepsilon_{d}$ is the dynamic strain; a, b are the fitting parameters

(6) Dynamic Stress Dynamic Strain East Eigenstructure Sensitivity Analysis

In order to investigate the effects of three factors, namely, the perimeter pressure, consolidation ratio and the number of wet and dry cycles, on the dynamic stress dynamic strain principal model, this paper designs orthogonal experiments with the maximum dynamic stress of cement:phosphogypsum:red clay = 6:47:47 mix as an example, and chooses L_9_ (3^3^) orthogonal table for the orthogonal analysis. The levels of each factor and the results of orthogonal test table are shown in Tables 17-18. The experimental data were processed by SPSS software to obtain the data results in Table 19, from which it can be seen that the effects of the number of dry and wet cycles and the perimeter pressure on this dynamic constitutive model are highly significant, and the effect of the consolidation ratio is more significant, the effect of the perimeter pressure > the effect of the number of dry and wet cycles > the effect of the consolidation ratio.

**Table 17:** Levels of factors in orthogonal test.

Level	Pressurization/KPa	Consolidation ratio	Number of dry and wet cycles/N
1	40	1	0
2	80	1.5	3
3	120	2	5

**Table 18:** Table of orthogonal tests.

Test number	Pressurization/KPa	Consolidation ratio	Number of dry and wet cycles/N	Maximum dynamic stress $\sigma_{d}\backslash )$/kPa
1	1	1	1	218.9924
2	1	2	2	183.7487
3	1	3	3	192.505
4	2	1	2	215.118
5	2	2	3	211.5
6	2	3	1	387.6306
7	3	1	3	258.176
8	3	2	2	329.9323
9	3	3	1	451.6886

**Table 19:** Orthogonal test results of cement:phosphogypsum:red clay = 6:47:47 mixes.

Independent variable	Degrees of freedom	Mean square	F	P	$R^{2}$
Number of dry and wet cycles/N	2	8295.123	149.316	0.007**	0.998
Pressurization/KPa	2	16469.975	296.467	0.003**	
Consolidation ratio	2	4987.46	89.777	0.011*	

Note: *p < 0.05 **p < 0.01.

### 3.2 Dynamic shear modulus-dynamic shear strain structural modeling

(1) Influence of envelope pressure

**Fig 8:**
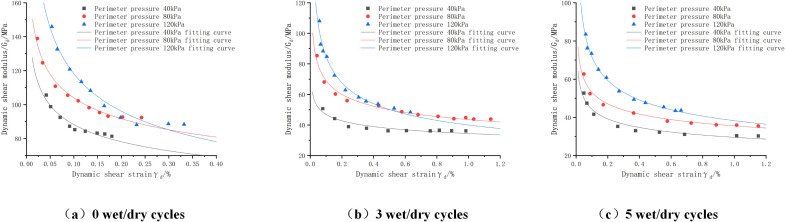
Dynamic shear modulus-dynamic shear strain curves of cement:phosphogypsum:red clay = 6:47:47 mixes with different enclosing pressures, consolidation ratio 1.5.

The dynamic shear modulus-dynamic shear strain curves of cement:phosphogypsum:red clay = 6:47:47 mixes under different enclosure pressures are shown in Fig 8, because the rest of the consolidation ratio has similar patterns, only the dynamic shear modulus-dynamic shear strain curves of the mixes with a consolidation ratio of 1.5, and the number of dry and wet cycles of 0, 3, and 5, and C:P:T = 6:47:47 mixes are shown. From the figure, we can see that the dynamic shear modulus is negatively correlated with the dynamic strain, and with the increase of the dynamic strain the dynamic shear modulus undergoes a trend of rapid decay followed by a gradual flattening. From the longitudinal view, it can be seen that at the same strain, the dynamic shear modulus increases with the increase of the peripheral pressure. This is mainly due to the fact that the increase in the perimeter pressure results in greater densification between the soils, which results in a more uniform force and less loss of force when subjected to dynamic loading.

The fitting parameters of dynamic shear modulus-dynamic shear strain under different enclosing pressures and the results of model evaluation are shown in Table 20, from which we can see that R^2^ is greater than 0.90, the maximum value of MAE is 2.635, and the maximum value of RMSE is 3.612, which is a good fit.

**Table 20:** Results of dynamic shear modulus-dynamic shear strain fitting parameters and model error assessment under different enclosure pressures.

Fitting equation	Number of wet and dry cycles/N	Pressurization/KPa	a	b	R^2^	MAE	RMSE
$G_{d}=\frac{1}{a{\gamma_{d}}^{b}}$	0	40	0.01696	0.17528	0.944	1.651	1.856
		80	0.0146	0.18133	0.980	1.666	2.056
		120	0.01682	0.29856	0.973	2.635	3.162
	3	40	0.02904	0.13482	0.901	1.365	1.579
		80	0.02297	0.20055	0.978	1.372	1.809
		120	0.02501	0.32668	0.976	2.322	2.968
	5	40	0.33383	0.17535	0.951	1.583	1.763
		80	0.02814	0.17472	0.974	1.142	1.395
		120	0.02613	0.2726	0.993	0.888	1.113

(2) Effect of consolidation ratio

**Fig 9:**
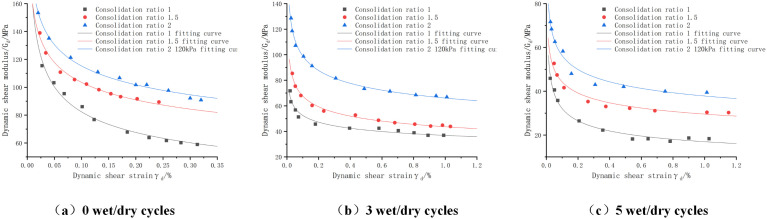
Dynamic shear modulus-dynamic shear strain curves of cement:phosphogypsum:red clay = 6:47:47 mixes with different consolidation ratios, perimeter pressure 80KPa.

The dynamic shear modulus-dynamic shear strain curves of cement:phosphogypsum:red clay = 6:47:47 mixes with different consolidation ratios are shown in Fig 9, and the results of data fitting at 80 KPa are shown only because of the similarity of the rest of the laws under the surrounding pressure. It can be seen from the figure that the dynamic shear modulus increases when the consolidation ratio increases, so there is a positive correlation between the two. This is mainly due to the fact that under the effect of increasing consolidation ratio, the gap between the mixture particles is squeezed, the porosity is reduced, and it becomes denser, which improves the ability of the soil body to resist external deformation.

The fitting parameters of dynamic shear modulus-dynamic shear strain under different consolidation ratios and the results of model evaluation are shown in Table 21, from which we can see that R^2^ is greater than 0.94, the maximum value of MAE is 2.009, and the maximum value of RMSE is 2.376, which is a good fit.

**Table 21:** Results of dynamic shear modulus-dynamic shear strain fitting parameters and model error assessment under different consolidation ratios.

Fitting equation	Number of wet and dry cycles/N	Consolidation ratio	a	b	R^2^	MAE	RMSE
$G_{d}=\frac{1}{a{\gamma_{d}}^{b}}$	0	1	0.02342	0.28522	0.985	2.009	2.376
		1.5	0.01485	0.18687	0.989	1.182	1.541
		2	0.01313	0.17991	0.993	1.321	1.557
	3	1	0.02713	0.15383	0.948	1.720	2.231
		1.5	0.02302	0.19228	0.992	1.018	1.218
		2	0.01518	0.17664	0.994	1.230	1.619
	5	1	0.05897	0.27756	0.985	1.108	1.251
		1.5	0.03383	0.17536	0.951	1.473	1.691
		2	0.02644	0.17261	0.970	1.625	2.101

(3) Effect of the number of wet and dry cycles

**Fig 10:**
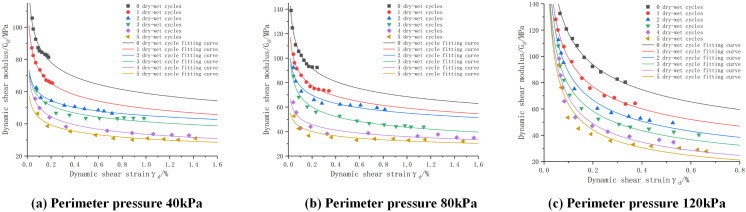
Dynamic shear modulus-dynamic shear strain curves for cement:phosphogypsum:red clay = 6:47:47 mixes with different number of wet and dry cycles, consolidation ratio 1.5.

The dynamic shear modulus-dynamic shear strain curves of cement:phosphogypsum:red clay = 6:47:47 mixes under different numbers of wet and dry cycles are shown in Fig 10, from which it can be seen that with the increase of the number of wet and dry cycles the dynamic shear modulus gradually decays, for example, at the peripheral pressure of 80KPa, 0, 3, and 5 times of wet and dry cycles: the maximum dynamic shear modulus of the mix decays from 138.939MPa to 52.704Mpa; the attenuation was 33.57% and 42.89%, respectively. This is mainly because in the process of wet and dry cycle, through repeated humidification and drying makes the cohesion of the mixture decreased, which makes the mixture resistance to shear weakened, and the shear modulus also decreased, another main factor is because the specimen in the process of humidification of the mixture of red clay will be water absorption and expansion, which produces inhomogeneous tensile stress, resulting in the further development of cracks, accelerating the mixture shear modulus decay. Decay.

The fitted parameters of dynamic shear modulus-dynamic shear strain with different number of wet and dry cycles and the results of model evaluation are shown in Table 22, from which we can see that the dynamic shear modulus-dynamic shear strain curves were fitted using the fitting equations in the table, with R^2^ greater than 0.85, and with the maximum values of MAE of 4.827 and RMSE of 5.990. However, the expression of this dynamic eigenstructural model also fails to express the relationship with the wet and dry cycles, therefore, this paper further analyzes the fitted data through the data in Table 18, and builds up the parameter The relationship equation between a, b and the number of wet and dry cycles is shown in Table 19, from which it can be seen that the fitting coefficients R^2^ are greater than 0.98 indicating that the relationship equation can better express the values of a, b. The relationship equation is then substituted into the modeling model and then into the modeling model. Immediately after substituting the relationship equation into the derivation of the ontological model, a new modified ontological model can be obtained as shown in Table 20.

**Table 22:** Dynamic shear modulus-dynamic shear strain fitting parameters and model error assessment results under dry and wet cycling.

Pressurization/KPa	Number of wet and dry cycles/N	a	b	R^2^	MAE	RMSE
40	0	0.01696	0.17528	0.944	1.651	1.856
	1	0.02017	0.17209	0.996	0.376	0.475
	2	0.02214	0.11681	0.965	0.630	0.883
	3	0.0242	0.13482	0.901	1.638	1.894
	4	0.02984	0.17704	0.976	0.920	1.201
	5	0.0326	0.15471	0.938	1.006	1.162
80	0	0.0146	0.18133	0.980	1.666	2.056
	1	0.01691	0.16866	0.977	1.274	1.531
	2	0.01814	0.14651	0.937	2.243	2.624
	3	0.02297	0.20055	0.978	1.380	1.783
	4	0.02789	0.15197	0.854	2.891	3.658
	5	0.03095	0.13281	0.855	1.982	2.369
120	0	0.01808	0.32739	0.995	1.303	1.470
	1	0.02308	0.35741	0.995	1.272	1.541
	2	0.02845	0.39891	0.982	2.553	2.997
	3	0.03393	0.42423	0.956	3.694	4.525
	4	0.04525	0.49337	0.926	4.633	5.763
	5	0.05319	0.5288	0.914	4.827	5.990

(4) Proposed dynamic shear modulus-dynamic shear stress variation constitutive model for phosphogypsum stabilized soil

It can be concluded from the fitted images in Figs 8–10, the fitted parameters in Tables 20–24 and the evaluation of MAE and RMSE models that Eq. (7) has a good fitting effect and low error for phosphogypsum stabilized soils with different perimeter pressures, consolidation ratios, and number of wet and dry cycles, and the modified dynamic constitutive equations based on the number of wet and dry cycles have a good fitting effect, so that the dynamic properties of phosphogypsum stabilized soils under different perimeter pressures, consolidation ratios, and number of wet and dry cycles can be represented by Eq. (7) and the equations in Tables 20 can be used to express the dynamic properties of phosphogypsum stabilized soil under wet and dry cycles. The derivation of Eq. (7) is shown below, which is mainly introduced by using the dynamic soil mechanics equation and combining Eqs. (2) to (6).

**Table 23:** Parameter a, b fitting relationship equation.

Pressurization/KPa	Parameter a relational equation	R^2^	Parameter b relational equation	R^2^
40	a = 0.01502*e^(-N/-6.998)+0.0022	0.984	b = 0.148 + 0.0347*sin(pi*(N-3.5514)/0.024)	0.989
80	a = 0.01223*e^(-N/-5.694)+0.0021	0.984	b = 0.0324 + 0.0347*sin(pi*(N + 0.375)/1.425)	0.894
120	a = 0.02218*e^(-N/-5.219)-0.0041	0.995	b = 0.31881 + 0.04115*N	0.982

Note: N is the number of wet/dry cycles.

**Table 24:** Modified eigenmodel.

Pressurization/KPa	Modified eigenstructural model
40	Gd=1(0.01502*e^(−N/−6.998)+0.0022gammad0.148+0.0347*sin(pi*(N−3.5514)/0.024)
80	Gd=1(0.01223*e^(−N/−5.694)+0.0021)γd0.0324+0.0347*sin(pi*(N+0.375)/1.425)
120	Gd=1(0.02218*e^(−N/−5.219)−0.0041gammadb=0.31881+0.04115*N

Note: N is the number of wet/dry cycles.


$$\sigma_{d}=\frac{\varepsilon_{d}}{a{\varepsilon_{d}}^{b}}$$ (2)

$$\tau_{d}=\frac{\sigma_{d}}{2}$$ (3)

$$G_{d}=\frac{\tau_{d}}{\gamma_{d}}$$ (4)

$$G_{d}=\frac{E_{d}}{2(1+\mu )}$$ (5)

$$\gamma_{d}=\epsilon_{d}(1+\mu )$$ (6)

$$G_{d}=\frac{1}{a{\gamma_{d}}^{b}}$$ (7)

Note: $G_{d}$ is the dynamic shear modulus; $\gamma_{d}$ is the dynamic shear strain;${\;\tau }_{d}$ is the dynamic shear stress; μ is Poisson’s ratio;$\gamma_{d\;}$is the dynamic shear strain;$E_{d\;}$is the dynamic modulus of elasticity;$\sigma_{d}$ is the dynamic stress; a, b are the fitting parameters

(5) Dynamic shear modulus dynamic shear stress structural sensitivity analysis

In order to investigate the effects of three factors, namely, the enclosing pressure, the consolidation ratio and the number of wet and dry cycles, on the dynamic shear modulus dynamic shear strain constitutive model, this paper designs orthogonal experiments with the maximum dynamic shear modulus of cement:phosphogypsum:red clay = 6:47:47 mixes as an example, and chooses the orthogonal table of L_9_ (3^3^) for orthogonal analysis in order to improve the robustness of the model. The levels of each factor and the orthogonal test table are shown in Tables 25–26. Through the SPSS software processing test data to get Table 27 data results, from the table can be seen, the number of dry and wet cycle influence is more significant, for the dynamic constitutive model, the influence of the number of dry and wet cycles is greater than the consolidation ratio is greater than the peripheral pressure, so for the wet and dry cycle of the study has a high value significance.

**Table 25:** Levels of factors in orthogonal test.

Level	Pressurization/KPa	Consolidation ratio	Number of dry and wet cycles/N
1	40	1	0
2	80	1.5	3
3	120	2	5

**Table 26:** Table of orthogonal tests.

Test number	Pressurization/KPa	Consolidation ratio	Number of dry and wet cycles/N
1	1	1	1
2	1	2	2
3	1	3	3
4	2	1	2
5	2	2	3
6	2	3	1
7	3	1	3
8	3	2	2
9	3	3	1

**Table 27:** Orthogonal test results of cement:phosphogypsum:red clay = 6:47:47 mixes.

Independent variable	Degrees of freedom	Mean square	F	P	$R^{2}$
Consolidation ratio	2	278.824	17.24	0.345	0.982
Number of dry and wet cycles/N	2	2665.79	19.696	0.0378*	
Pressurization/KPa	2	125.85	8.65	0.766	

Note: *p < 0.05 **p < 0.01.

### 3.3 Dynamic resilience modulus eigenmodel for mixes

(1) Influence of circumferential pressure on dynamic resilience modulus

**Fig 11:**
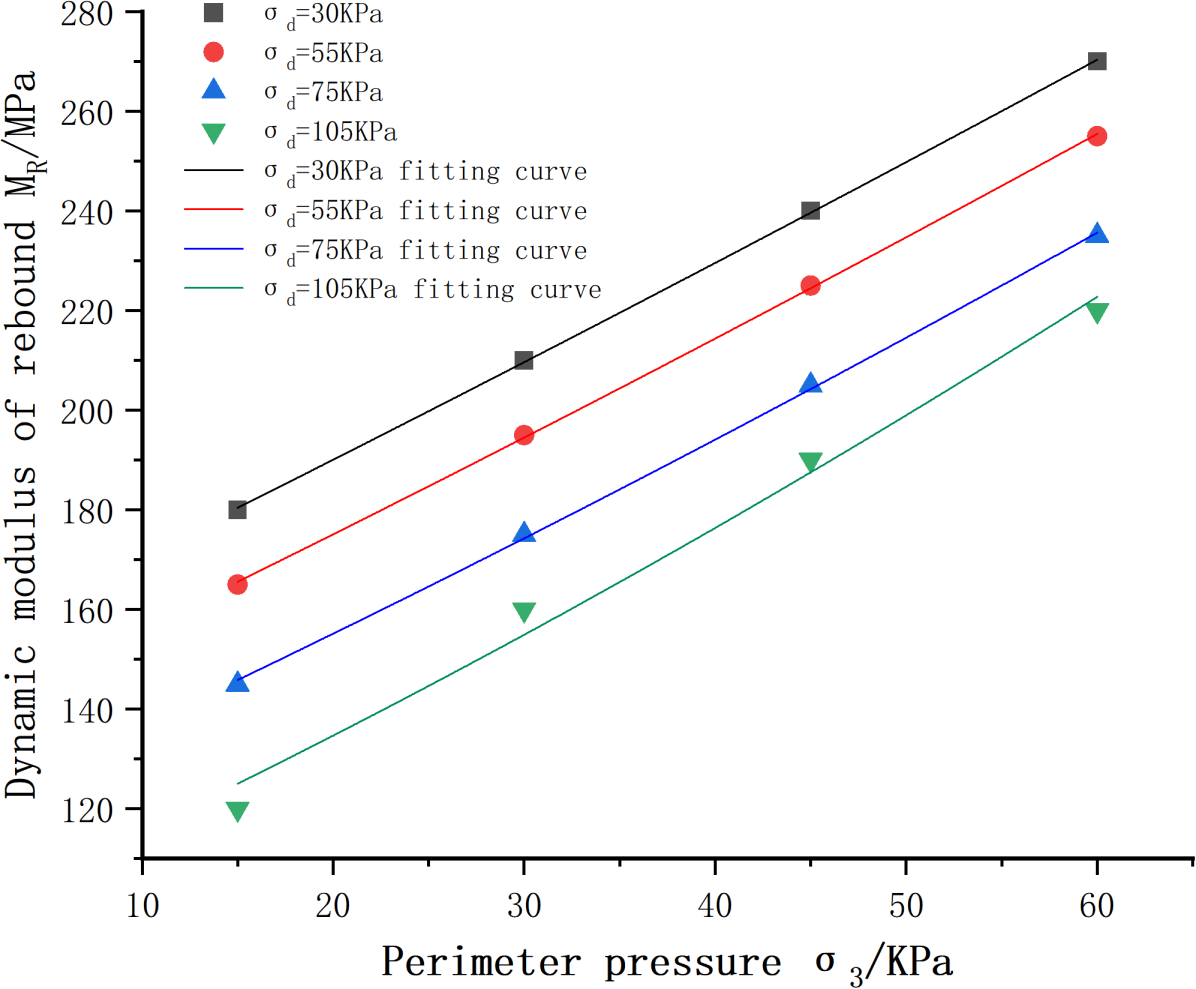
Dynamic resilient modulus-perimeter pressure variation curves of cement:phosphogypsum:red clay = 6:47:47 mixes under different bias stresses.

The dynamic resilient modulus-perimeter pressure fitting curve is shown in Fig 11. Since the variation rules of other cases are similar, they are not repeated here. As can be seen from the figure, under the same bias stress, the dynamic resilience modulus shows an increasing trend with the increase of the circumferential pressure, and this increasing trend is almost linear. This is mainly due to the fact that as the circumferential pressure increases, the degree of compactness between the internal structures rises, and the mix is close to being an elastomer. In the longitudinal direction, when we fix the value of the circumferential pressure and observe the change of the dynamic modulus of resilience under different bias stresses, it can be seen that the dynamic modulus of resilience decreases with the increase of the bias stress. This is mainly due to the fact that the increase of bias stress will cause the particles to be broken or rearranged. Particle fragmentation leads to an increase in porosity within the specimen, which in turn reduces the overall stiffness of the specimen. At the same time, the rearrangement of particles will also change the internal structure of the specimen, making it more susceptible to deformation. Specifically, when the bias stress increases from 30 kN to 55 kN, 75 kN, and then to 105 kN, the corresponding dynamic modulus of rebound decreases from 210 Kpa to 195 Kpa, 175 Kpa, and finally to 160 Kpa, respectively.During this process, the reduction of dynamic modulus of rebound is roughly maintained in the range of 15–20 Kpa, and the percentage reduction is 7.14%, 10.2% and 8.57%, showing a more stable decreasing trend. The dynamic rebound modulus versus perimeter pressure curve fitting parameters and model error assessment results are shown in Table 28, from which it can be seen that R^2^ are greater than 0.98, MAE, RMSE maximum values of 1.533, 4.023, respectively, the fit is good. Therefore Ni model can be used to predict the dynamic resilient modulus values of phosphogypsum stabilized soil without wet and dry cycles.

**Table 28:** Dynamic modulus of rebound versus perimeter pressure curve fitting parameters and model error assessment results.

Bias stress/KPa	$k_{1}$	$k_{2}$	$k_{3}$	R^2^	MAE	RMSE
30	1.07966	1.22461	1.30455	0.998	0.148	0.370
55	0.94213	1.31303	0.86794	0.999	0.205	0.515
75	0.81852	1.45281	0.66941	0.998	0.297	0.745
105	0.67500	1.74847	0.51847	0.988	1.533	4.023

(2) Effect of the number of wet and dry cycles

**Fig 12:**
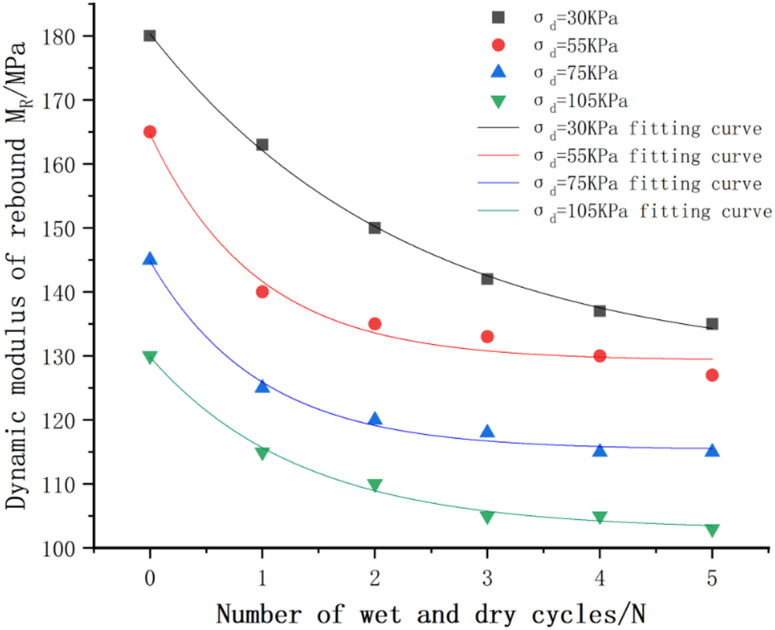
Variation curve of dynamic resilience modulus of cement:phosphogypsum:red clay = 6:47:47 mix under bias stress – number of wet and dry cycles.

The relationship between the dynamic resilience modulus of the mix and the number of wet and dry cycles under different bias stresses is shown in Fig 12. From the figure, it can be seen that: the dynamic resilience modulus of the mix and the number of wet and dry cycles show a negative correlation, with the increase of the number of wet and dry cycles, the dynamic resilience modulus modulus gradually decreases [41], which is mainly due to the fact that the incorporation of the wet and dry cycles will destroy the internal structure of the mix, weakening the support of the skeleton particles. As the number of times is superimposed, the unfavorable effect is intensified. But further we can find that the first two dry and wet cycles have a greater influence on the dynamic resilience modulus, and the slope of the curve gradually decreases when it reaches the third time, which indicates that the dry and wet cycles are no longer the main factors affecting the dynamic resilience modulus after three times.

Dynamic rebound modulus and the number of wet and dry cycles of the relationship between curve fitting parameters and model error assessment results are shown in Table 29, from the table can be seen that R^2^ are greater than 0.98, MAE, RMSE maximum value of 1.370, 1.621, respectively, indicating that the fit is good, so can be used to predict the value of the dynamic rebound modulus of phosphogypsum-stabilized soils affected by wet and dry cycles of Eq. (9).

**Table 29:** Results of curve fitting parameters and model error evaluation of dynamic modulus of resilience versus number of wet and dry cycles.

Bias stress/KPa	$k_{1}$	$k_{2}$	$k_{3}$	R^2^	MAE	RMSE
30	128.268	1.16213	2.31662	0.998	0.544	0.589
55	129.291	0.88999	0.94956	0.983	1.370	1.621
75	115.375	0.7835	0.97228	0.993	0.770	0.842
105	102.866	0.70812	1.34242	0.994	0.651	0.708

(3) Proposed intrinsic model for dynamic rebound modulus of phosphogypsum stabilized soil

By evaluating the fitted images in Figs 11-12 above and the fitted parameters in Tables 28-29 with the MAE and RMSE models, it can be seen that the Ni model (as shown in Eq. (8)) has a better fit as well as a lower error to the experimental data, and it can be used to study the relationship between the dynamic resilience modulus of the mix and the bias stress and perimeter compression, whereas the relationship with the wet and dry cycles is fitted by introducing Eq. e^(−N/k3) on the basis of the power exponential model as shown in Eq. (9) is fitted.


MR=k1pa(σ3/pa+1)^k2(σd/pa+1)^k3 (8)

Note: $M_{R}$ for the dynamic resilience modulus;$k_{1}$, $k_{2}$, $k_{3}$ for the model parameters; $p_{a}$ for the absolute value of atmospheric pressure, usually take 100KPa;$\;\sigma_{3}$ for the surrounding pressure; $\sigma_{d}$ for the bias stress;


MR=k1+σd^k2*e^(−N/k3) (9)

Note: $k_{1}$, $k_{2}$, $k_{3}$ are model parameters

(4) Dynamic modulus of resilience sensitivity analysis

In order to explore the effect of each factor on the dynamic resilience modulus of the mix, to do orthogonal experimental analysis with cement:phosphogypsum:red clay = 6:47:47 mix as an example, choose L_9_ (3^3^) orthogonal table, the level of each factor and the orthogonal table as shown in Table 30-31. Through the SPSS software to process the experimental data to obtain the results of the test in Table 32, from the table, it can be seen that the effect of the perimeter pressure on the dynamic resilience modulus of the mixture is more significant, from the F-value can be seen that the effect of the perimeter pressure > the number of dry and wet cycles > the consolidation ratio.

**Table 30:** Levels of factors in orthogonal test.

Level	Pressurization/KPa	Bias stress/KPa	Number of dry and wet cycles/N
1	15	30	0
2	30	55	3
3	45	75	5

**Table 31:** Table of orthogonal tests.

Test number	Pressurization/KPa	Bias stress/KPa	Number of dry and wet cycles/N	Dynamic modulus of resilience/Mpa
1	1	1	1	180
2	1	2	2	145
3	1	3	3	115
4	2	1	2	170
5	2	2	3	155
6	2	3	1	175
7	3	1	3	190
8	3	2	2	175
9	3	3	1	205

**Table 32:** Orthogonal test results of cement:phosphogypsum:red clay = 6:47:47 mixes.

Independent variable	Degrees of freedom	Mean square	F	p	R^2^
Pressurization/KPa	2	1411.11	19.844	0.048*	0.975
Bias stress/KPa	2	442.778	6.227	0.138	
Number of dry and wet cycles/N	2	951.11	13.375	0.070	

Note: *P < 0.05**P < 0.01.

#### 3.3.1 Comparison of dynamic and static resilience modulus.

In the application of phosphogypsum stabilized red clay as roadbed material, the current specification and academic research have not yet established an exact performance index value, and the specification only provides a general range, which is of limited significance in guiding the actual engineering design and construction. In view of this, this paper innovatively selects the dynamic resilience modulus value under extreme adverse conditions of 15Kpa perimeter pressure and 105KN bias stress as the benchmark, aiming to provide a safer and more reliable reference basis for engineering practice.

**Fig 13:**
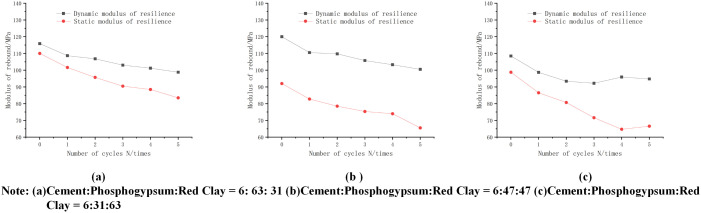
Representative values of dynamic resilience modulus of mixes with different mix ratios-dry and wet cycle variation curves.

By comparing and analyzing the changes in dynamic and static resilient modulus for different ratios (cement:phosphogypsum:red clay) with different numbers of wet and dry cycles (shown in Fig 13), we find that the dynamic resilient modulus is generally higher than the static value, and the difference gradually increases with the increase in the number of wet and dry cycles. However, from the figure, we can see that the dynamic and static resilience modulus tends to stabilize with the increase of wet and dry cycles, which indicates that further cycles have little effect on the dynamic modulus. It is particularly noteworthy that the difference between the dynamic and static resilient modulus reaches a maximum of 31.2 MPa when the ratio is cement:phosphogypsum:red clay = 6:31:63 and the fourth wet/dry cycle is performed, whereas the difference between the two is the smallest, only 5.88 MPa, when the ratio is cement:phosphogypsum:red clay = 6:63:31 and no wet/dry cycle is performed.

Further, under the most unfavorable conditions (i.e., ratio of 6:31:63 with five wet and dry cycles), the representative value of dynamic modulus of resilience of the mix decreased to 94.75Mpa, which is lower than the preset safety threshold of 100Mpa, a finding that is important for evaluating the long term performance of the material and optimizing the design of the proportioning.

The representative and static values of dynamic modulus of resilience for the most unfavorable condition with perimeter pressure of 15 Kpa and bias stress of 105 are shown in Table 33 below:As can be seen from Table 23, the recommended value of dynamic resilience modulus of cemented phosphogypsum stabilized soil under dry and wet cycles is 90−100 MPa, and under the same conditions, the value of dynamic resilience modulus is about 1.1–1.5 times of static resilience modulus.

**Table 33:** Dynamic and static modulus of resilience values.

specifications	C:P:T = 6:47:47		C:P:T = 6:63:31		C:P:T = 6:31:63	
Number of wet and dry cycles	dynamic value(MPa)	static value(MPa)	dynamic value(MPa)	static value(MPa)	dynamic value(MPa)	static value(MPa)
0	120.000	92.000	115.880	110.000	108.460	98.760
1	110.500	82.740	108.640	101.600	98.700	86.500
2	109.750	78.500	106.800	95.700	93.400	80.700
3	105.750	75.320	103.000	90.440	92.200	71.620
4	103.250	74.000	101.170	88.470	95.900	64.700
5	100.500	65.530	98.780	83.480	94.750	66.540
Average of last three	103.17	71.62	100.98	87.46	94.28	67.62
Dynamic/static	1.44		1.15		1.39	
	1.1-1.5					

## 4 Conclusion

In this paper, the following conclusions were obtained by various methods such as fitting analysis, model error analysis, and sensitivity analysis of the dynamic triaxial experimental data of phosphogypsum stabilized soil by adding the effect of dry and wet cycles:

(1) The effect of wet and dry cycles has a greater impact on the dynamic constitutive model of the mix, Monismith exponential model is suitable for the dynamic stress-strain curve under the wet and dry cycles, the minimum fitting coefficient is 0.90, and the maximum MAE and RMSE values are 4.0065 and 4.5077, respectively, while the proposed modified constitutive model based on the number of wet and dry cycles also has a better fitting effect, the minimum fitting coefficient is 0.80.
(2) The dynamic shear modulus is negatively correlated with the dynamic shear strain, increasing the number of wet and dry cycles decreases the dynamic shear modulus, while increasing the consolidation ratio and perimeter pressure has a certain promotion effect on the dynamic shear modulus. The intrinsic equation $G_{d}=\frac{1}{a{\gamma_{d}}^{b}}$ derived using soil dynamics provides a good fit to the dynamic shear modulus-dynamic shear strain of phosphogypsum stabilized soil with a low error.
(3) The dynamic resilience modulus of the mix was analyzed by Ni model and power index model, and the dynamic resilience modulus was negatively correlated with the bias stress, the number of wet and dry cycles, and positively correlated with the peripheral pressure. Comparing the dynamic and static resilience modulus under different phosphogypsum and red viscous ratios, it can be seen that the dynamic resilience modulus is higher than the static resilience modulus, and the enhancement ratio reaches 1.09–1.48 times, with the range of 5.8–35MPa, and the difference is significant.
(4) A sensitivity analysis of the above models using orthogonal experiments shows that the number of wet and dry cycles and the enclosing pressure have highly significant effects on the dynamic stress-dynamic strain constitutive model, and the effect of consolidation ratio is more significant. For the dynamic shear modulus-dynamic shear strain constitutive model, the effect of the number of wet and dry cycles is more significant. For the dynamic resilience modulus prediction model, the effect of perimeter pressure is more significant. Therefore, the study of wet and dry cycles is of high value.
(5) By comparing the representative value of dynamic resilience modulus with the static value under the most unfavorable conditions with peripheral pressure of 15Kpa and bias stress of 105kPa, it is concluded that the value of dynamic resilience modulus of phosphogypsum-stabilized soil under dry and wet cycles is 1.1–1.5 times of the static resilience modulus, and the dynamic resilience modulus is recommended to be 90–100MPa, which can provide scientific basis for the design of the road pavement.
(6) In summary, the kinetic constitutive model of phosphogypsum stabilized soil proposed in this paper can provide certain reference for the resource utilization of phosphogypsum, and can solve the problem of poor engineering properties of red clay with high liquid limit and high plastic limit to a certain extent, but due to the regional differences, the results obtained from the test materials in different regions have certain effects, so it is necessary to further carry out a lot of practice and research, from which experience can be summarized to enrich the of phosphogypsum stabilized red clay.

## Supporting information

S1 Data Minimum data set. (DOCX)

## References

[1] Junpeng L, Wei T. Research on the application of phosphogypsum in highway roadbed. Low Carbon World. 2018;(11):227–8.

[2] Duan C. Experimental study on the dynamic characteristics of improved loess under acidic environment. Xi’an University of Technology. 2024.

[3] Xie D. Soil dynamics. Xi’an: Xi’an Jiaotong University Press. 2004.

[4] Kallioglou P, Tika TH, Pitilakis K. Shear modulus and damping ratio of cohesive soils. Journal of Earthquake Engineering. 2008;12(6):879–913.

[5] Yuan P, Zhu L, Zhong X, et al. Experimental study on the dynamic characteristics of site soil reinforced by enzyme-induced calcium carbonate precipitation. Geotechnics. 2022;43(12):3385–92.

[6] Uzan J. Characterization of Granular Materials. Washington D C: Transportation Research Board, National Research 1022, Transportation Research Board, National Research Council. 1985.

[7] Witczak MW, Uzan J. The Universal Airport Pavement Design System - Report I of V: Granular Material Characterization. Washington D C: Department of Civil Engineering, University of Maryland; 1988.

[8] Lytton RL, Uzan J, Fernando EG. Development and validation of performance prediction models and specifications for asphalt binders and paving mixes. SHRP A-357. Washington D C: Strategic Highway Research Program, National Research Council; 1993.

[9] NCHRP Project 1-28. Laboratory Determination of Resilient Modulus for Flexible Pavement Design-Final Report. Washington DC: National Cooperative Highway Research Program, Transportation Research Board, National Research Council, 1997.

[10] Hongyuan F, Qiyi Y, Ling Z, et al. Dynamic resilience of pre-disintegrated carbonaceous mudstone under the influence of multiple factors. Journal of Civil Engineering. 2024;57(02):117–28.

[11] Dai i, Peng J, Wei R, Li L, Lin H. Improvement in dynamic behaviors of cement-stabilized soil by super-absorbent-polymer under cyclic loading. Soil Dynamics and Earthquake Engineering. 2022;163:107554.

[12] Duan S, Jiang Q, Xu D, Liu G. Experimental Study of Mechanical Behavior of Interlayer Staggered Zone under Cyclic Loading and Unloading Condition. Int J Geomech. 2020;20(3). doi: 10.1061/(asce)gm.1943-5622.0001602

[13] Zhang P, Fei K, Dai D. Modeling of granular soil-structure interface under monotonic and cyclic loading with the nonlinear approach. Computers and Geotechnics. 2023;159.

[14] Xie L i. Static-dynamic characterization and constitutive modeling of saturated red clay. Jiangxi University of Science and Technology. 2020.

[15] Wang J, Hou S, Lin Z. Study on dynamic properties of reinforced gravelly soil under semi-sinusoidal cyclic traffic dynamic load. Vibration and Shock. 2022;41(03):90–8.

[16] Pingbao Y, Min Z, Wei H, et al. Dynamic properties and constitutive modeling of nickel-iron slag-clay under cyclic loading . Chinese Journal of Highway. 2024;37(04): 155–65.

[17] Dongxue L, Jianming L, Jinsong Q, et al. Evolution of resilient modulus of clayey subgrade soil under humidity cycle. Journal of Tongji University (Natural Science Edition),2013,41(07):1051–5.

[18] Zhangfeng LI, Xinwen CAO, Chunlei WANG. Experimental study on the feasibility of using phosphogypsum-amended soil as roadbed fill. Roadbed Engineering, 2008(05): 157–8.

[19] Silva MV, Rezende LR de, Mascarenha MM dos A. Phosphogypsum, tropical soil and cement mixtures for asphalt pavements under wet and dry environmental conditions. Resources, Conservation and Recycling. 2019;144:123–36.

[20] Huang WD, Chen KS, Zhang BL. Dynamic characterization of cement-phosphogypsum stabilized red clay under dry and wet cycles. Journal of Guangxi University (Natural Science Edition). 2023;48(06):1316–30.

[21] Ran W, Chen H, Li L. Evolution of rebound modulus of coarse-grained soil under dry and wet cycles and model prediction and modification. Journal of Jilin University (Engineering Edition). 2021;51(06):2079–86.

[22] Chen J, Chen K, Liu Z. Research on the expansion, shrinkage properties and fracture evolution of red clay stabilised with phosphogypsum under dry-wet cycles. PLoS One. 2024;19(8):e0308616. doi: 10.1371/journal.pone.0308616 39163397 PMC11335147

[23] Kun Z, Kai-Sheng C. Design of mix ratio of phosphogypsum stabilized soil based on orthogonal test[J]. China Water Transportation(Next Half Month),2023,23(07):151-152.

[24] Mingkai Z, Xiaoqiao Z, Xiao C, et al. Research on the performance of cement phosphogypsum stabilized gravel pavement base material. Highway,2016,61(04):186-190.

[25] Bo P, Wenyong S, Hongwei Z, et al. Research on mechanical properties and reasonable dosage of comprehensive stabilized soil with phosphogypsum. New Building Materials,2020,47(08):86-90+127.

[26] Chen K-S, Luo G-F, Zhou B, et al. Experimental study on the dynamic characteristics of lime-phosphate gypsum stabilized red clay. Journal of Chongqing Jiaotong University (Natural Science Edition). 2023;42(10):75–82.

[27] Institute of Highway Science, Ministry of Transportation. Geotechnical test specification for highway:JTG E40-2007[M]. People’s Transportation Press, 2007.

[28] Chen K. Triaxial test of compacted red clay under dry and wet cycle. Highway. 2017;62(11):215–20.

[29] Yunli T. Characterization of unconfined compressive strength of Yunnan red clay under dry and wet cycling. Kunming University of Science and Technology, 2021.

[30] Que Y, Yao X. Capillary characterization of granite residual soil embankment under high water table conditions. Journal of Fuzhou University (Natural Science Edition). 2011;39(05):754–9.

[31] Li P. Monitoring and analysis of internal water content of highway subgrade. Shanxi Construction. 2021;47(12):104–6.

[32] Zhi HU, Lianbo AI, Zhichao LI, et al. Resistivity evolution of compacted pulverized clay under dry-wet cycling-dynamic load penetration coupling. Geotechnics, 2021.

[33] Hao Z. Research on mechanical properties and slope stability of red clay under dry and wet cycles. Beijing Jiaotong University. 2019.

[34] Kaisheng C. Research on shear strength characteristics of red clay under dry and wet cycling. Highway. 2016;61(02):45–9.

[35] Yingzi XU, Chao SU, Dezhi LIU, et al. Effect of root doping rate on cracking of expansive soils under dry and wet cycles[J]. Science Technology and Engineering,2022,22(12):4938–44.

[36] Min LI, Wen LI, Yitong C, et al. Three-dimensional curves of soil moisture characteristics under natural expansion and contraction of dry and wet cycles in expansive soils[J]. Journal of Irrigation and Drainage,2023,42(01):121-129.

[37] Monismith C, Ogawa N, Freeme C. Permanent deformation characteristics of subgrade soils due to repeated loading. Transportation Research Record Journal of the Transportation Research Board. 1975;537:1–17.

[38] Zhang B. Research on road performance of cemented phosphogypsum stabilized red clay under dry and wet cycles. Guizhou University. 2023.

[39] Jie FU, Chengchun HAO, Siqi Y, et al. Blasting criterion for free boundary problems with unpressurized new Hooke-type elastic dynamics. Science China:Mathematics. 2024;1–16.

[40] Stanway R, Sproston J, Stevens NG. Non-linear modeling of an electro-rheological vibration damper. J Electrostatics. 1987;20:167–84.

[41] Changgui LI, Jiankun HU. Experimental study on dynamic resilient modulus of high liquid limit pulverized soil under dry and wet cycle. Chinese and foreign highway,2021,41(03):347–51.

