# Supplementary material for: Dynamic principal modeling of cemented phosphogypsum stabilized soil under dry and wet cycles
Source: PLoS One. 2025 Aug 6;20(8):e0316643. doi: 10.1371/journal.pone.0316643 (PMC12327651; doi:10.1371/journal.pone.0316643)
Supplement: S1 Data — (DOCX) [file pone.0316643.s001.docx]

**Minimum data set**

**Table .1 Physical mechanics of phosphogypsum and chemical composition test**

| Testing Indicators | retrieve a value |  | Chemical composition | mass fraction/% |
| --- | --- | --- | --- | --- |
| Fineness/(%) | 43.5 |  | SO_3_ | 49.07 |
| $\rho$/(g/cm3) | 2.26 |  | CaO | 40.07 |
| SSA/(m2/kg) | 101 |  | SiO_2_ | 5.78 |
| WS/(%) | 5.4 |  | F | 1.89 |
| LOI/(%) | 18.42 |  | P_2_O_5_ | 1.35 |
| Alkali content/(%) | 1.30 |  | Na_2_O | 0.587 |
| Mass fraction of sulfur trioxide/(%) | 0.07 |  | Al_2_O_3_ | 0.435 |
|  |  |  | Fe_2_O_3_ | 0.210 |

Table .2 Phosphogypsum radioactivity test results

| Testing Program | CRa  /(Bq·kg-1) | CTh  /(Bq·kg-1) | Ck  /(Bq·kg-1) | internal irradiance index(IRa) | external exposure index(Ir) | |  |
| --- | --- | --- | --- | --- | --- | --- | --- |
| Technical Requirements |  |  |  | ≤1.0 | | ≤1.0 | |
| Test results | 56.26 | 3.84 | 26.65 | 0.3 | | 0.2 | |
| Individual judgment |  |  |  | Eligible (voter etc) | | Eligible (voter etc) | |

Table. 3 Phosphogypsum heavy metal content test results

| Test element | Constant volume V_0_/mL | Test Solution Element Concentration  C_0_/ug·L^-1^ | Dilution factor  /f | Elemental concentration  C_1_/mg·L^-1^ | Elemental content  C_x_/mg·kg^-1^ | Testing Conclusion |
| --- | --- | --- | --- | --- | --- | --- |
| （Cd） | 10 | 0.0620 | 50 | 3.10 | 0.26 | Meets the requirements of national standards |
| （Pb） | 10 | 1.5270 | 50 | 76.35 | 6.33 |  |
| （Cr） | 10 | 0.2920 | 50 | 14.60 | 0.96 |  |
| （As） | 10 | 0.1284 | 50 | 6.42 | 0.42 |  |
| （Hg） | 10 | 0.0484 | 50 | 2.42 | 0.16 |  |

Table. 4 Basic physical indexes and chemical composition of red clay

| physical index | retrieve a value | chemical composition | mass fraction/% |
| --- | --- | --- | --- |
| $\omega$/% | 57.88 | SiO_2_ | 54.16 |
| $W_{P}$/% | 52.13 | Al_2_O_3_ | 28.70 |
| $W_{L}$/% | 74.65 | Fe_2_O_3_ | 10.36 |
| plasticity index | 22.52 | Si | 26.90 |
| $\omega_{op}$/% | 32.12 | Al | 15.90 |
| $\rho_{dmax}$/g·cm^-3^ | 1.482 | Fe | 7.94 |
|  |  | else | 3.61 |

| Table. 5 Basic Cement Parameters | | | | | |
| --- | --- | --- | --- | --- | --- |
| Heat loss  /% | SO_3_  /% | Alkali content  /% | Incipient condensation time  /min | Time of final coagulation  /min | Stability |
| 1.58 | 2.87 | 2.42 | 302 | 322 | Eligible |
| Cl^-^/% | Gypsum content  /% | 3-Day Flexural Strength  /MPa | 28-Day Flexural Strength  /MPa | 3-day compressive strength  /MPa | 28-day compressive strength  /MPa |
| 0.018 | 5.00 | 5.0 | 6.7 | 24.9 | 43.7 |

Table. 6 Dynamic strain and dynamic strain test program

| Frequency/（HZ） | Pressurization/（kPa） | Consolidation ratio | Number of cycles N/times |
| --- | --- | --- | --- |
| 2  2  2 | 40 | 1 | 0、1、2、3、4、5 |
|  |  | 1.5 | 0、1、2、3、4、5 |
|  |  | 2 | 0、1、2、3、4、5 |
|  | 80 | 1 | 0、1、2、3、4、5 |
|  |  | 1.5 | 0、1、2、3、4、5 |
|  |  | 2 | 0、1、2、3、4、5 |
|  | 120 | 1 | 0、1、2、3、4、5 |
|  |  | 1.5 | 0、1、2、3、4、5 |
|  |  | 2 | 0、1、2、3、4、5 |

Table. 7 Loading sequence of fine-grained soil specimens

| Load Serial Number | peripheral compressive stress  σ_3_（Kpa） | contact stress  σ_c_（Kpa） | cyclic stress  σ_d_（Kpa） | axial stress  σ_max_（Kpa） | Number of Load Actions |
| --- | --- | --- | --- | --- | --- |
| 0 | 30 | 6 | 55 | 61 | 1000 |
| 1 | 60 | 12 | 30 | 42 | 100 |
| 2 | 45 | 9 | 30 | 39 | 100 |
| 3 | 30 | 6 | 30 | 36 | 100 |
| 4 | 15 | 3 | 30 | 33 | 100 |
| 5 | 60 | 12 | 55 | 67 | 100 |
| 6 | 45 | 9 | 55 | 64 | 100 |
| 7 | 30 | 6 | 55 | 61 | 100 |
| 8 | 15 | 3 | 55 | 58 | 100 |
| 9 | 60 | 12 | 75 | 87 | 100 |
| 10 | 45 | 9 | 75 | 84 | 100 |
| 11 | 30 | 6 | 75 | 81 | 100 |
| 12 | 15 | 3 | 75 | 78 | 100 |
| 13 | 60 | 12 | 105 | 117 | 100 |
| 14 | 45 | 9 | 105 | 117 | 100 |
| 15 | 30 | 6 | 105 | 111 | 100 |
| 16 | 15 | 3 | 105 | 108 | 100 |

| 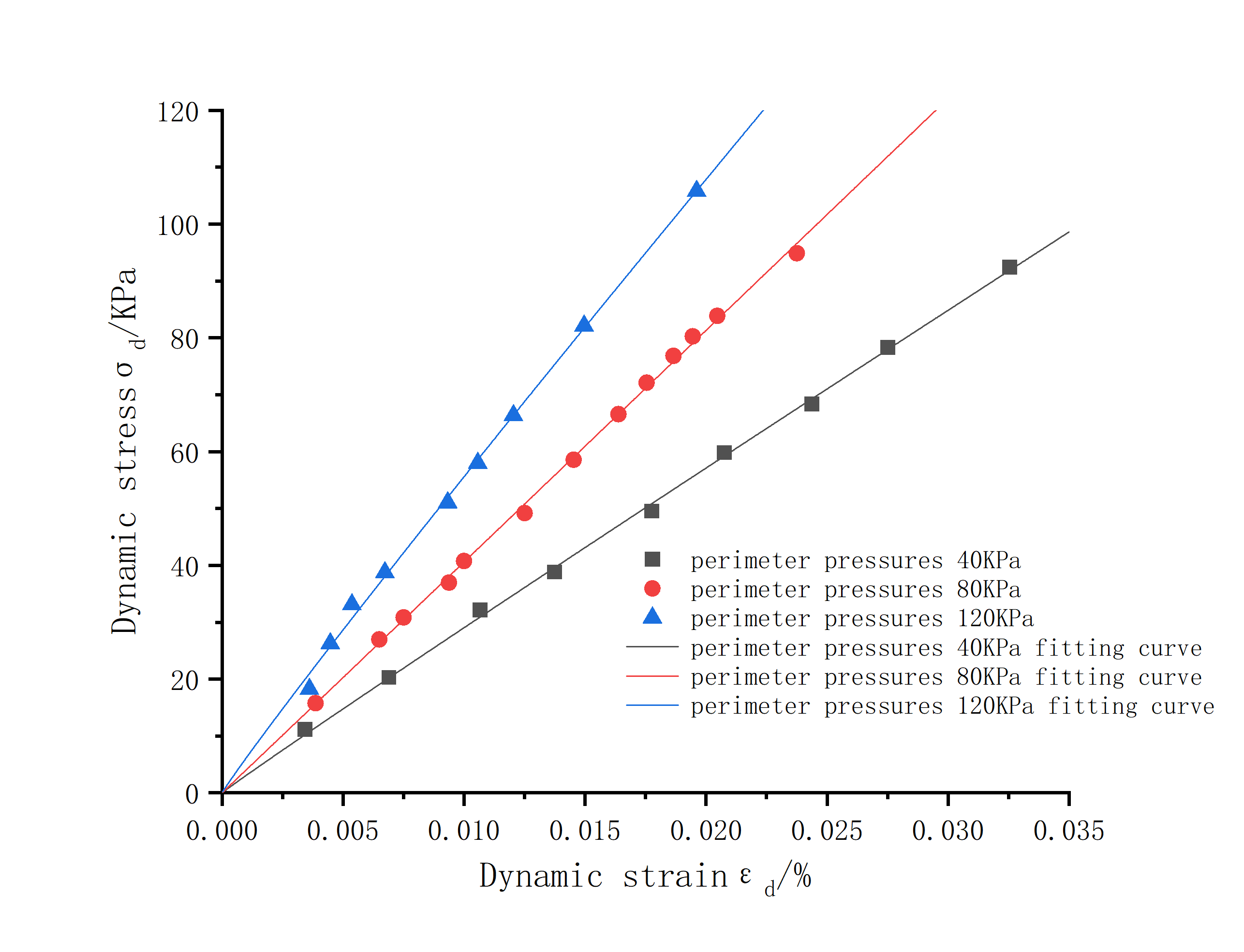 | 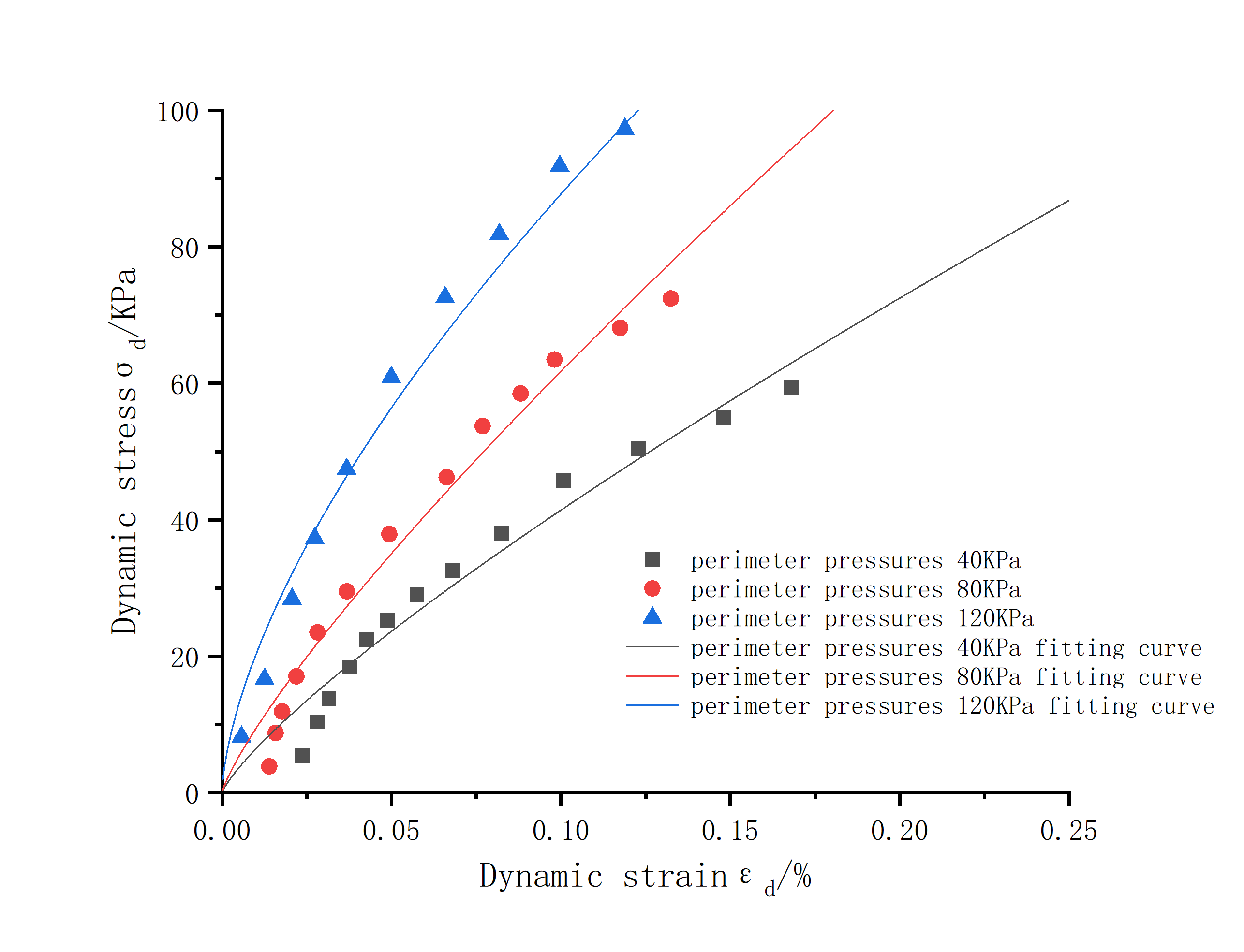 |
| --- | --- |
| **（a）0 wet/dry cycles** | **（b）3 wet/dry cycles** |
| 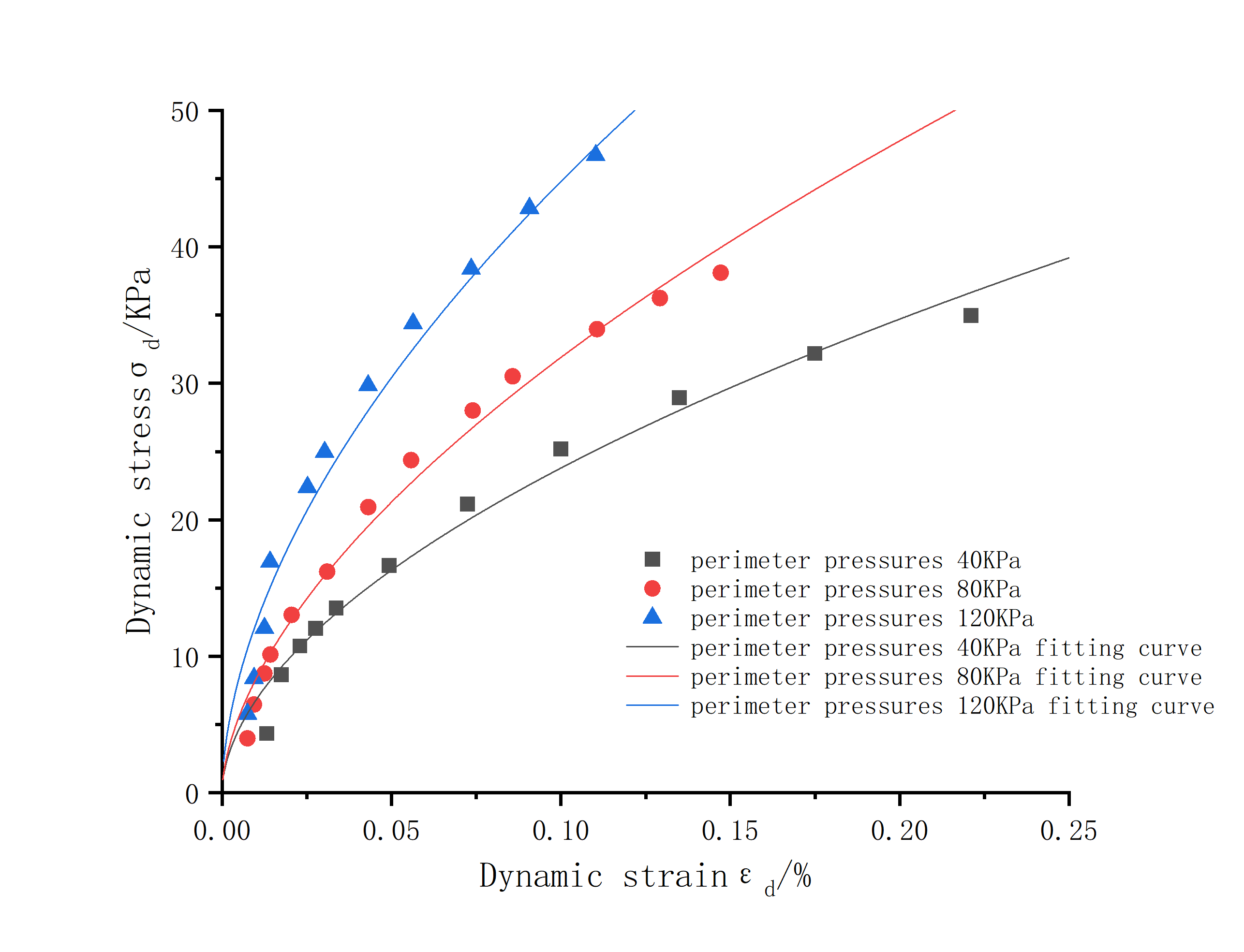 | |
| **（c）5wet/dry cycles**  Fig. 4 Dynamic stress-strain curves of cement:phosphogypsum:red clay=6:47:47 mixes with different enclosure pressures, consolidation ratio | |

**Raw data for Figure 4**

| 0 wet/dry cycles | | | | | |
| --- | --- | --- | --- | --- | --- |
| dynamic stress/% | Dynamic strain/KPa | dynamic stress/% | Dynamic strain/KPa | dynamic stress/% | Dynamic strain/KPa |
| 0.00344 | 11.184 | 0.00387 | 15.767 | 0.00361 | 18.2302 |
| 0.0069 | 20.279 | 0.0065 | 26.967 | 0.00447 | 26.2652 |
| 0.01066 | 32.121 | 0.0075 | 30.844 | 0.00537 | 33.12898 |
| 0.01375 | 38.837 | 0.00938 | 36.943 | 0.00673 | 38.7446 |
| 0.01775 | 49.511 | 0.01 | 40.751 | 0.00932 | 51.0585 |
| 0.02075 | 59.818 | 0.0125 | 49.191 | 0.01057 | 57.9725 |
| 0.02438 | 68.349 | 0.01453 | 58.542 | 0.01204 | 66.3677 |
| 0.0275 | 78.307 | 0.01638 | 66.58 | 0.01496 | 82.1343 |
| 0.03254 | 92.423 | 0.01754 | 72.124 | 0.01961 | 105.7894 |
|  |  | 0.01865 | 76.845 |  |  |
|  |  | 0.01945 | 80.275 |  |  |
|  |  | 0.02046 | 83.846 |  |  |
|  |  | 0.02375 | 94.873 |  |  |

| 3 wet/dry cycles | | | | | |
| --- | --- | --- | --- | --- | --- |
| dynamic stress/% | Dynamic strain/KPa | dynamic stress/% | Dynamic strain/KPa | dynamic stress/% | Dynamic strain/KPa |
| 0.02379 | 5.456 | 0.01394 | 3.876 | 0.00574 | 8.2462 |
| 0.02826 | 10.377 | 0.01585 | 8.782 | 0.01261 | 16.7082 |
| 0.03156 | 13.756 | 0.01775 | 11.927 | 0.02067 | 28.41998 |
| 0.03777 | 18.409 | 0.022 | 17.061 | 0.02742 | 37.3006 |
| 0.04278 | 22.416 | 0.02819 | 23.532 | 0.03682 | 47.4275 |
| 0.04878 | 25.278 | 0.03688 | 29.515 | 0.04996 | 60.9065 |
| 0.05754 | 28.982 | 0.04939 | 37.893 | 0.06583 | 72.5887 |
| 0.06819 | 32.621 | 0.06629 | 46.221 | 0.08186 | 81.8473 |
| 0.0825 | 38.031 | 0.0769 | 53.721 | 0.09968 | 91.8384 |
| 0.1007 | 45.703 | 0.08813 | 58.496 | 0.11888 | 97.2726 |
| 0.123 | 50.457 | 0.09813 | 63.497 | 0.1482 | 104.59263 |
| 0.148 | 54.916 | 0.11752 | 68.126 |  |  |
| 0.168 | 59.457 | 0.1325 | 72.426 |  |  |
| 0.191 | 62.653 | 0.1545 | 75.45 |  |  |

| 5 wet/dry cycles | | | | | |
| --- | --- | --- | --- | --- | --- |
| dynamic stress/% | Dynamic strain/KPa | dynamic stress/% | Dynamic strain/KPa | dynamic stress/% | Dynamic strain/KPa |
| 0.01325 | 4.348 | 0.00754 | 3.985 | 0.00754 | 5.784 |
| 0.01749 | 8.653 | 0.00944 | 6.462 | 0.00944 | 8.4123 |
| 0.023 | 10.752 | 0.01253 | 8.742 | 0.01253 | 12.075 |
| 0.0276 | 12.0475 | 0.01432 | 10.138 | 0.01418 | 16.9192 |
| 0.03374 | 13.531 | 0.02056 | 13.041 | 0.02523 | 22.39998 |
| 0.04941 | 16.65 | 0.03106 | 16.202 | 0.03029 | 24.9706 |
| 0.07248 | 21.137 | 0.04319 | 20.935 | 0.04313 | 29.8475 |
| 0.09997 | 25.198 | 0.05581 | 24.372 | 0.05638 | 34.3855 |
| 0.135 | 28.935 | 0.07399 | 28.011 | 0.07353 | 38.3787 |
| 0.175 | 32.189 | 0.08579 | 30.511 | 0.09075 | 42.8443 |
| 0.221 | 34.954 | 0.11067 | 33.966 | 0.11028 | 46.7084 |
|  |  | 0.12927 | 36.247 | 0.13118 | 51.0226 |
|  |  | 0.14721 | 38.096 | 0.16151 | 57.56263 |
|  |  | 0.18253 | 40.35 |  |  |
|  |  | 0.213 | 42.36 |  |  |

Table .8 Dynamic stress-dynamic strain fitting parameters for different constitutive equations

| Fitting equation | | Number of dry and wet cycles/N | Perimeter pressures/KPa | | R^2^ | | |  |  |  |
| --- | --- | --- | --- | --- | --- | --- | --- | --- | --- | --- |
| Monismith exponential：$\sigma_{d}=a*\varepsilon_{d}^{b}$ | | 0 | 120 | | 0.997 | | |  |  |  |
|  |  | 3 | 40 | | 0.961 | | |  |  |  |
|  |  | 5 | 80 | | 0.941 | | |  |  |  |
| empirical kinetic isomorphism model^34^$\sigma_{d}=\frac{\varepsilon_{d}}{a{\varepsilon_{d}}^{b}}$ | | 0 | 40 | | 0.991 | | |  |  |  |
|  |  | 3 | 40 | | 0.922 | | |  |  |  |
|  |  | 5 | 120 | | 0.977 | | |  |  |  |
| Hardin-Drnevich hyperbola^35^：$\sigma_{d}=\frac{\varepsilon_{d}}{a*\varepsilon_{d}+b}$ | | 0 | 80 | | 0.325 | | |  |  |  |
|  |  | 3 | 40 | | 0.653 | | |  |  |  |
|  |  | 5 | 80 | | 0.606 | | |  |  |  |
| Davidenkov model^36^：$H(\gamma)=({\frac{({\gamma/\gamma0)}^{2B}}{1+({\gamma/\gamma0)}^{28}})}^{A}$ | | 0 | 120 | | 0.668 | | |  |  |  |
|  |  | 3 | 120 | | 0.832 | | |  |  |  |
|  |  | 5 | 40 | | 0.394 | | |  |  |  |
| Hooke model^37^：$\sigma_{d}=c\varepsilon_{d}$ | | 0 | 120 | | 0.995 | | |  |  |  |
|  |  | 3 | 120 | | 0.832 | | |  |  |  |
|  |  | 5 | 40 | | 0.646 | | |  |  |  |
| Bingham model^38^：$\sigma_{d}$=$\sigma_{0}+c\varepsilon_{d}$ | | 0 | 120 | | 0.997 | | |  |  |  |
|  |  | 3 | 40 | | 0.901 | | |  |  |  |
|  |  | 5 | 120 | | 0.891 | | |  |  |  |
| Table .9 Dynamic stress-dynamic strain fitting parameters under different dry and wet cycles and different perimeter pressures | | | | | | | | | |  |
| Fitting equation | | Number of dry and wet cycles/N | | Perimeter pressures/KPa | R^2^ | | MAE | RMSE | | |
| $\sigma_{d}=a*\varepsilon_{d}^{b}$ | | 0 | | 40 | 0.999 | | 0.7577 | 0.8475 | | |
|  |  |  |  | 80 | 0.998 | | 0.7613 | 0.9275 | | |
|  |  |  |  | 120 | 0.997 | | 0.9423 | 1.3234 | | |
|  |  | 3 | | 40 | 0.961 | | 4.2009 | 4.7936 | | |
|  |  |  |  | 80 | 0.972 | | 3.7517 | 4.5338 | | |
|  |  |  |  | 120 | 0.977 | | 2.9019 | 3.5273 | | |
| $\sigma_{d}=\frac{\varepsilon_{d}}{a{\varepsilon_{d}}^{b}}$ | | 5 | | 40 | 0.980 | | 0.9574 | 1.3643 | | |
|  |  |  |  | 80 | 0.977 | | 1.6617 | 1.9199 | | |
|  |  |  |  | 120 | 0.982 | | 1.7910 | 2.1433 | | |

Note: where is the dynamic stress;$\varepsilon_{d}$ is the dynamic strain;

| 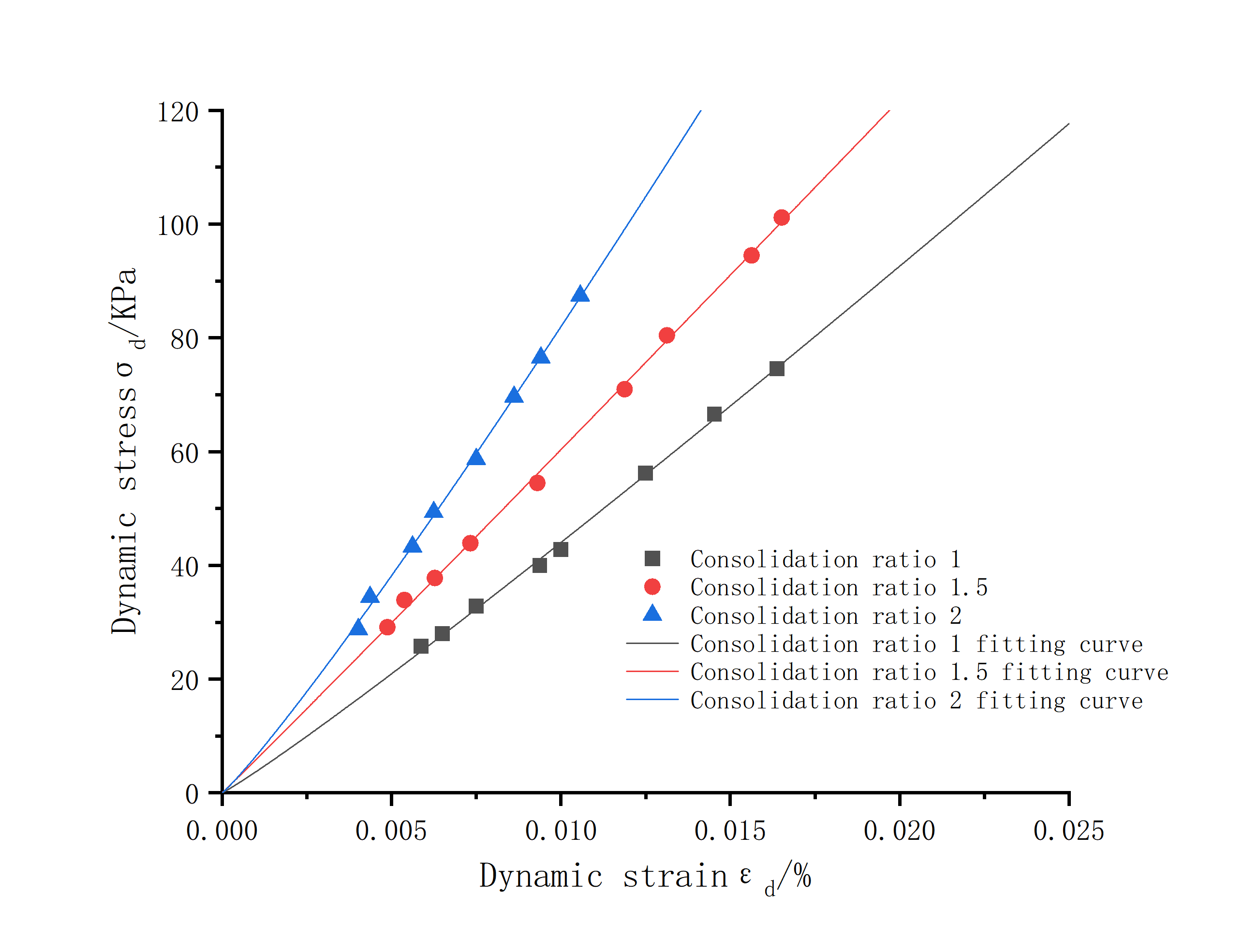 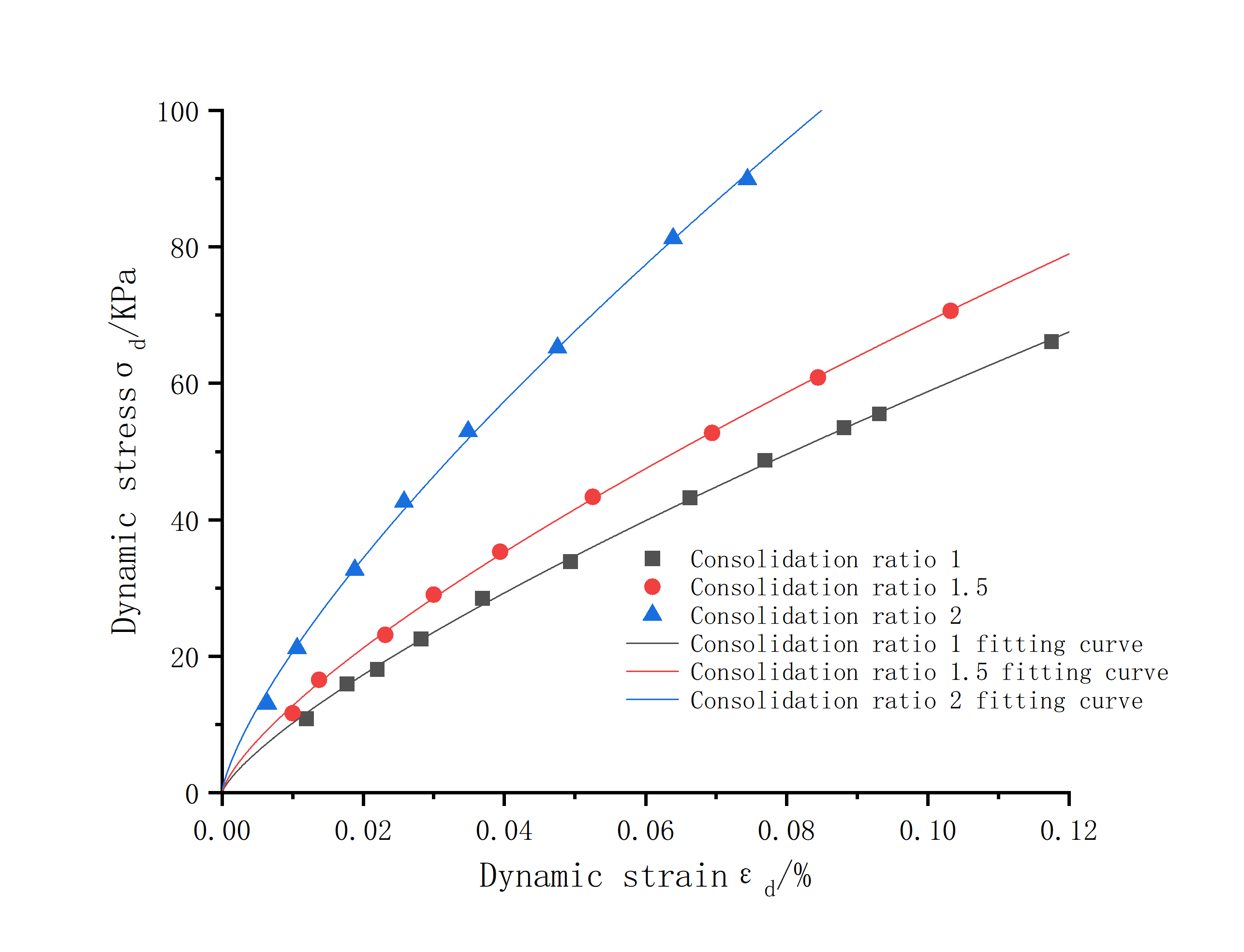  **（a）0 wet/dry cycles （b）3 wet/dry cycles**  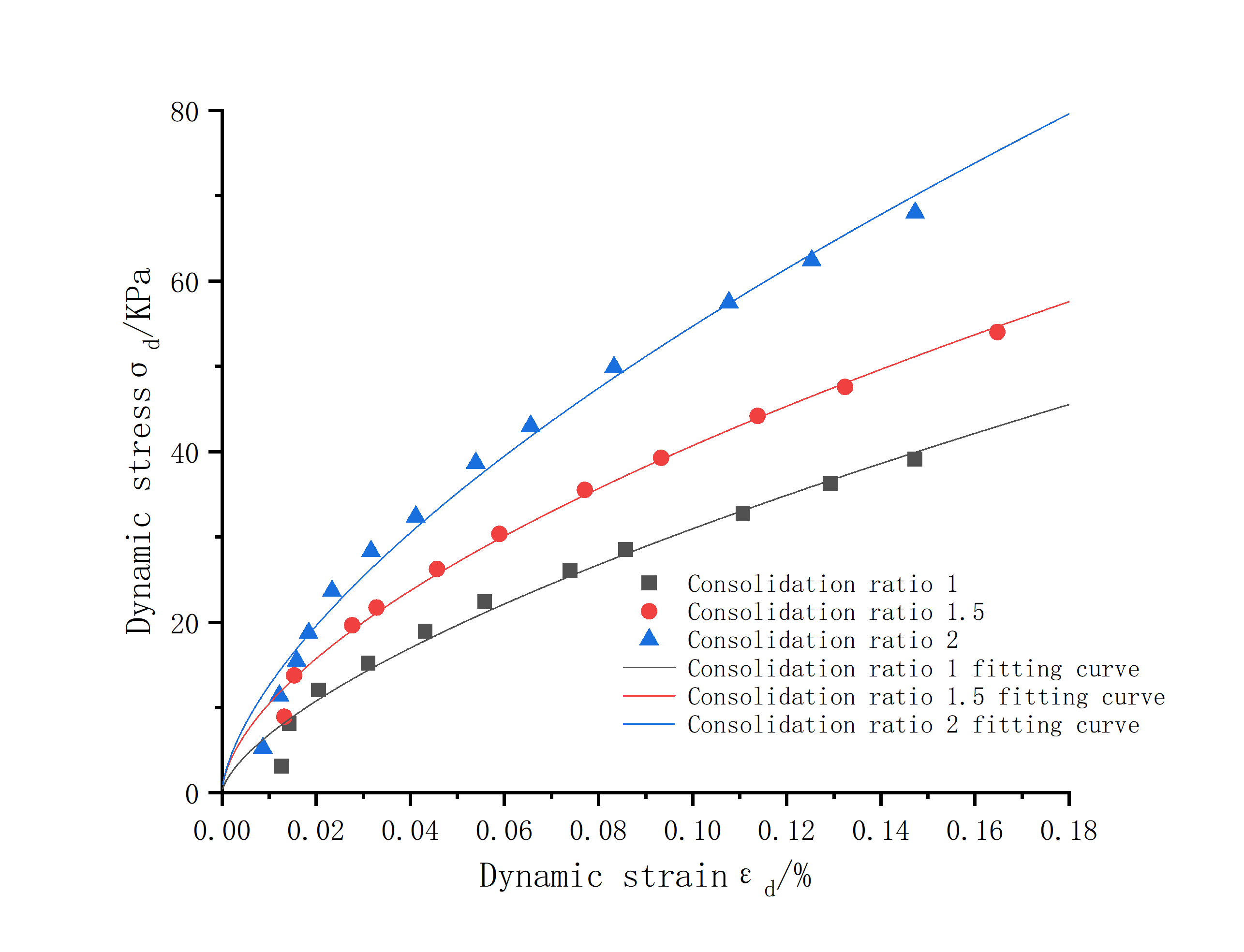  **（c）5 wet/dry cycles** |
| --- |

Fig. 5 Dynamic stress-strain curves of cement:phosphogypsum:red clay=6:47:47 mixes with different consolidation ratios, perimeter pressure 80kPag

**Raw data for Figure 5**

| 0 wet/dry cycles | | | | | |
| --- | --- | --- | --- | --- | --- |
| dynamic stress/% | Dynamic strain/KPa | dynamic stress/% | Dynamic strain/KPa | dynamic stress/% | Dynamic strain/KPa |
| 0.00587 | 25.767 | 0.00488 | 29.107 | 0.00402 | 28.7853 |
| 0.0065 | 27.967 | 0.00538 | 33.8943 | 0.00437 | 34.426 |
| 0.0075 | 32.844 | 0.00628 | 37.7566 | 0.00562 | 43.301 |
| 0.00938 | 39.943 | 0.00733 | 43.87 | 0.00625 | 49.341 |
| 0.01 | 42.751 | 0.00931 | 54.486 | 0.0075 | 58.685 |
| 0.0125 | 56.191 | 0.01188 | 70.946 | 0.00862 | 69.64 |
| 0.01453 | 66.542 | 0.01313 | 80.419 | 0.00941 | 76.54 |
| 0.01638 | 74.58 | 0.01563 | 94.5 | 0.01057 | 87.423 |
|  |  | 0.01652 | 101.146 |  |  |

| 3 wet/dry cycles | | | | | |
| --- | --- | --- | --- | --- | --- |
| dynamic stress/% | Dynamic strain/KPa | dynamic stress/% | Dynamic strain/KPa | dynamic stress/% | Dynamic strain/KPa |
| 0.01194 | 10.876 | 0.01 | 11.661 | 0.00646 | 13.006 |
| 0.01775 | 15.927 | 0.01375 | 16.55 | 0.01063 | 21.2 |
| 0.022 | 18.061 | 0.02313 | 23.161 | 0.01882 | 32.681 |
| 0.02819 | 22.532 | 0.03 | 29.042 | 0.02578 | 42.654 |
| 0.03688 | 28.515 | 0.03939 | 35.315 | 0.03487 | 52.955 |
| 0.04939 | 33.893 | 0.05252 | 43.353 | 0.04752 | 65.203 |
| 0.06629 | 43.221 | 0.06942 | 52.721 | 0.06389 | 81.241 |
| 0.0769 | 48.721 | 0.08442 | 60.859 | 0.07442 | 89.882 |
| 0.08813 | 53.496 | 0.1032 | 70.616 |  |  |
| 0.09313 | 55.497 |  |  |  |  |
| 0.11752 | 66.126 |  |  |  |  |

| 5 wet/dry cycles | | | | | |
| --- | --- | --- | --- | --- | --- |
| dynamic stress/% | Dynamic strain/KPa | dynamic stress/% | Dynamic strain/KPa | dynamic stress/% | Dynamic strain/KPa |
| 0.01263 | 3.128 | 0.01325 | 8.913 | 0.00871 | 5.258 |
| 0.01432 | 8.138 | 0.01532 | 13.761 | 0.0122 | 11.411 |
| 0.02056 | 12.041 | 0.02769 | 19.641 | 0.01587 | 15.4637 |
| 0.03106 | 15.202 | 0.03287 | 21.712 | 0.01842 | 18.7463 |
| 0.04319 | 18.935 | 0.0457 | 26.235 | 0.02338 | 23.661 |
| 0.05581 | 22.372 | 0.05894 | 30.332 | 0.03165 | 28.324 |
| 0.07399 | 26.011 | 0.07712 | 35.511 | 0.04118 | 32.375 |
| 0.08579 | 28.511 | 0.09331 | 39.256 | 0.05394 | 38.682 |
| 0.11067 | 32.766 | 0.1138 | 44.186 | 0.06559 | 43.031 |
| 0.12927 | 36.247 | 0.1324 | 47.567 | 0.08331 | 49.879 |
| 0.14721 | 39.096 | 0.16473 | 53.996 | 0.10769 | 57.486 |
|  |  |  |  | 0.1253 | 62.417 |
|  |  |  |  | 0.14728 | 67.996 |

Table. 10 Fitting parameters of dynamic stress-strain curves for mixes with different consolidation ratios

| Fitting equation | Number of dry and wet cycles/N | Consolidation ratio | R^2^ | MAE | RMSE |
| --- | --- | --- | --- | --- | --- |
| $\sigma_{d}=a*\varepsilon_{d}^{b}$ | 0 | 1 | 0.998 | 0.5953 | 0.7347 |
|  |  | 1.5 | 0.998 | 0.7352 | 0.9432 |
|  |  | 2 | 0.998 | 0.5386 | 0.7608 |
|  | 3 | 1 | 0.999 | 0.4016 | 0.4962 |
|  |  | 1.5 | 0.999 | 0.4082 | 0.4904 |
|  |  | 2 | 0.998 | 0.7261 | 0.9525 |
| $\sigma_{d}=\frac{\varepsilon_{d}}{a{\varepsilon_{d}}^{b}}$ | 5 | 1 | 0.978 | 1.1121 | 1.6458 |
|  |  | 1.5 | 0.993 | 0.7495 | 1.1406 |
|  |  | 2 | 0.986 | 1.7853 | 2.3315 |

Note: where $\sigma_{d}$ is the dynamic stress;$\varepsilon_{d}$ is the dynamic strain;

| \| 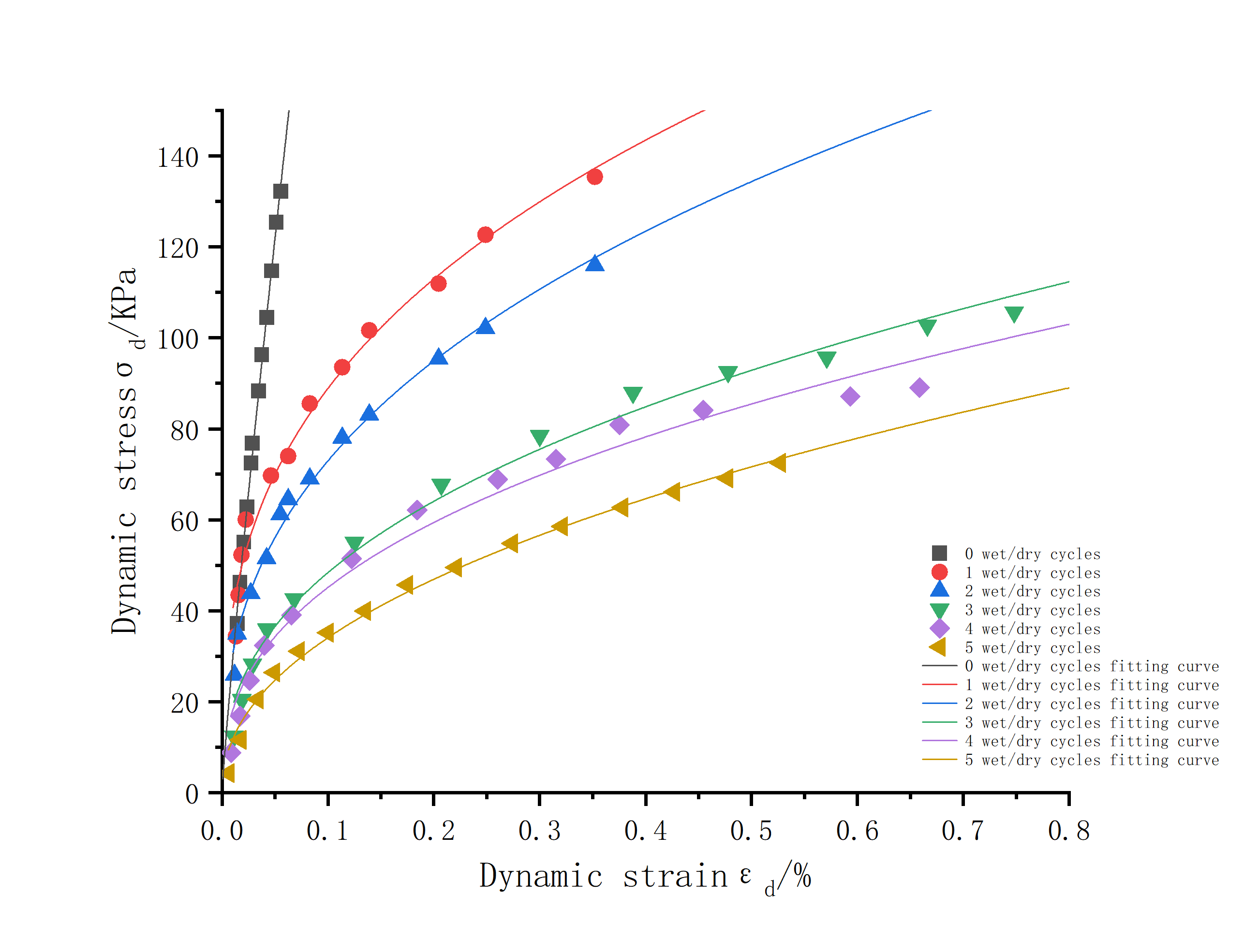 \| 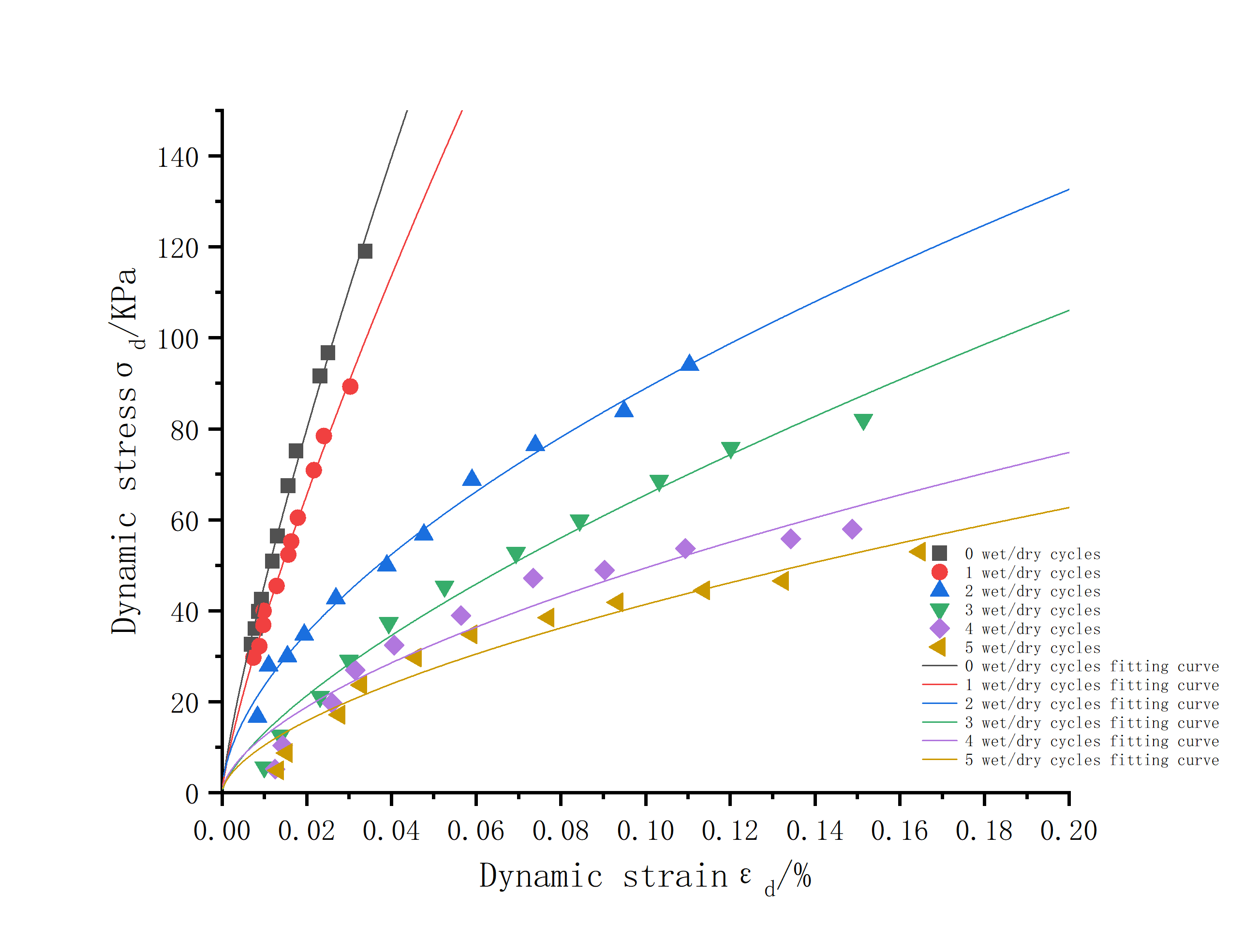 \| \| --- \| --- \| \| **(a)Perimeter pressure 40kPa, consolidation ratio 1.5 (b) Perimeter pressure 80kPa, consolidation ratio 1.5**  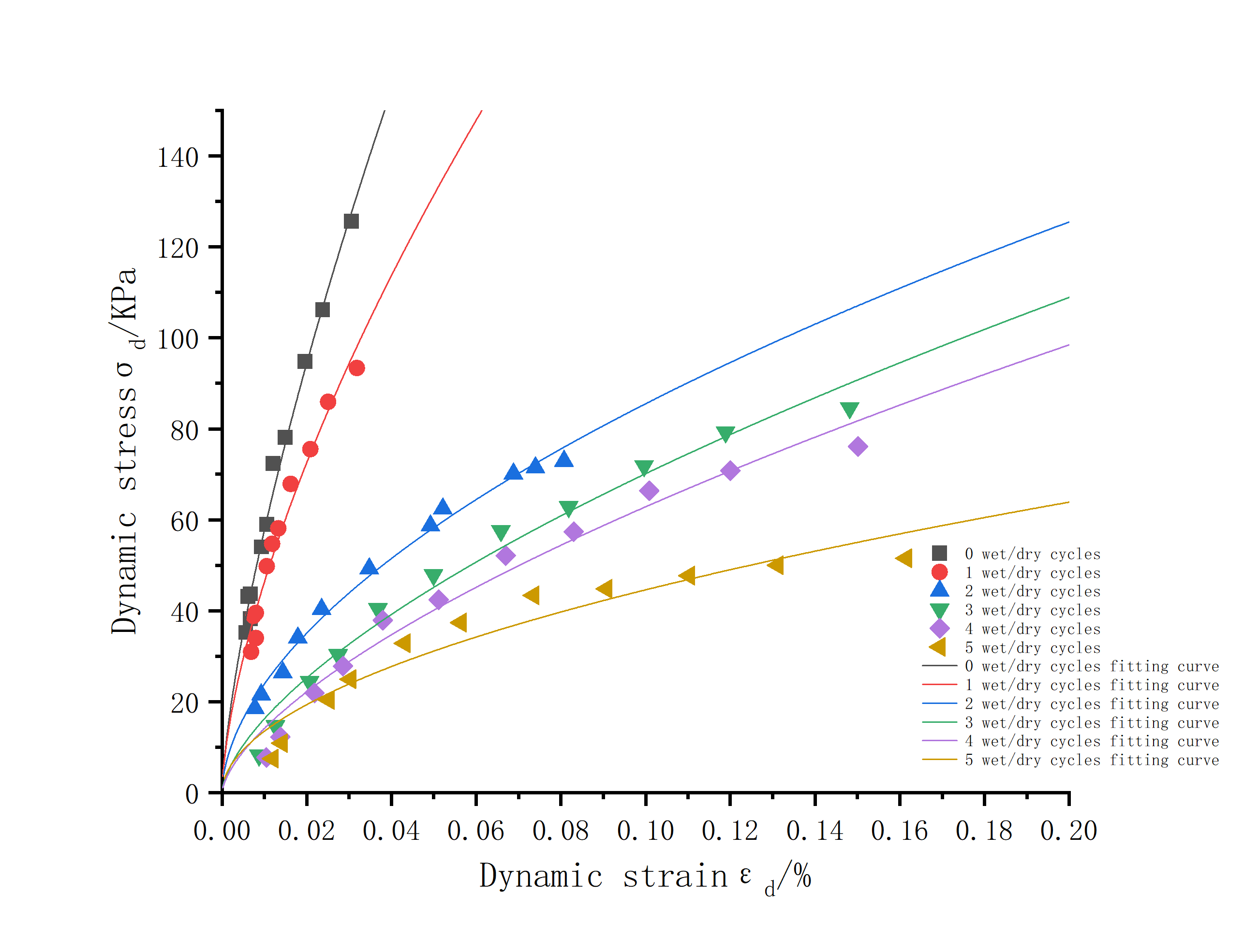 \| \| \| **(c) Perimeter pressure 120kPa, consolidation ratio 1.5** \| \| \| **Fig. 6 Dynamic stress-strain curve of cement:phosphogypsum:red clay=6:47:47 mix with different number of dry and wet cycles**   \| **Raw data for Figure 6** \| \| \| \| \| \| \| \| \| \| \| \| \| --- \| --- \| --- \| --- \| --- \| --- \| --- \| --- \| --- \| --- \| --- \| --- \| \| Perimeter pressure 40kPa \| \| \| \| \| \| \| \| \| \| \| \| \| dynamic stress/% \| Dynamic strain/KPa \| dynamic stress/% \| Dynamic strain/KPa \| dynamic stress/% \| Dynamic strain/KPa \| dynamic stress/% \| Dynamic strain/KPa \| dynamic stress/% \| Dynamic strain/KPa \| dynamic stress/% \| Dynamic strain/KPa \| \| 0.0144 \| 37.184 \| 0.0131 \| 34.38859 \| 0.01192 \| 25.90368 \| 0.01126 \| 12.377 \| 0.00861 \| 8.81 \| 0.00625 \| 4.348 \| \| 0.0169 \| 46.279 \| 0.0156 \| 43.48359 \| 0.01442 \| 34.99882 \| 0.01877 \| 20.409 \| 0.01712 \| 16.842 \| 0.01749 \| 11.653 \| \| 0.02066 \| 55.121 \| 0.01836 \| 52.32559 \| 0.02718 \| 43.84125 \| 0.02878 \| 28.278 \| 0.02613 \| 24.711 \| 0.03374 \| 20.531 \| \| 0.02375 \| 62.837 \| 0.02245 \| 60.04159 \| 0.04213 \| 51.55703 \| 0.04254 \| 35.982 \| 0.03989 \| 32.415 \| 0.04941 \| 26.465 \| \| 0.0275 \| 72.511 \| 0.04621 \| 69.71559 \| 0.05503 \| 61.23076 \| 0.06819 \| 42.621 \| 0.06554 \| 39.054 \| 0.07248 \| 31.137 \| \| 0.02875 \| 76.818 \| 0.06253 \| 74.02259 \| 0.06253 \| 64.53822 \| 0.125 \| 55.031 \| 0.12235 \| 51.464 \| 0.09997 \| 35.198 \| \| 0.03438 \| 88.349 \| 0.08299 \| 85.55359 \| 0.08299 \| 69.06897 \| 0.207 \| 67.703 \| 0.18435 \| 62.136 \| 0.135 \| 39.935 \| \| 0.0375 \| 96.307 \| 0.1135 \| 93.51159 \| 0.1135 \| 78.02668 \| 0.3 \| 78.457 \| 0.26025 \| 68.89 \| 0.175 \| 45.689 \| \| 0.04254 \| 104.423 \| 0.13902 \| 101.62759 \| 0.13902 \| 83.14265 \| 0.388 \| 87.916 \| 0.31535 \| 73.349 \| 0.221 \| 49.554 \| \| 0.0469 \| 114.707 \| 0.20443 \| 111.91159 \| 0.20443 \| 95.42701 \| 0.478 \| 92.457 \| 0.37535 \| 80.89 \| 0.274 \| 54.811 \| \| 0.05128 \| 125.437 \| 0.24881 \| 122.64159 \| 0.24881 \| 102.157 \| 0.571 \| 95.653 \| 0.45445 \| 84.086 \| 0.321 \| 58.53 \| \| 0.05568 \| 132.209 \| 0.3522 \| 135.41359 \| 0.3522 \| 115.92921 \| 0.666 \| 102.702 \| 0.59335 \| 87.135 \| 0.378 \| 62.69 \| \|  \|  \|  \|  \|  \|  \| 0.748 \| 105.652 \| 0.65873 \| 89.085 \| 0.427 \| 66.166 \| \|  \|  \|  \|  \|  \|  \|  \|  \|  \|  \| 0.477 \| 69.119 \| \|  \|  \|  \|  \|  \|  \|  \|  \|  \|  \| 0.527 \| 72.497 \|  \| Perimeter pressure 80kPa \| \| \| \| \| \| \| \| \| \| \| \| \| --- \| --- \| --- \| --- \| --- \| --- \| --- \| --- \| --- \| --- \| --- \| --- \| \| dynamic stress/% \| Dynamic strain/KPa \| dynamic stress/% \| Dynamic strain/KPa \| dynamic stress/% \| Dynamic strain/KPa \| dynamic stress/% \| Dynamic strain/KPa \| dynamic stress/% \| Dynamic strain/KPa \| dynamic stress/% \| Dynamic strain/KPa \| \| 0.00687 \| 32.645 \| 0.00742 \| 29.729 \| 0.0084 \| 16.748 \| 0.01 \| 5.661 \| 0.01253 \| 5.221 \| 0.01325 \| 4.913 \| \| 0.00788 \| 36.107 \| 0.00884 \| 32.231 \| 0.01103 \| 27.975 \| 0.01375 \| 12.55 \| 0.01432 \| 10.432 \| 0.01532 \| 8.761 \| \| 0.00863 \| 39.87 \| 0.00974 \| 36.895 \| 0.01543 \| 30.012 \| 0.02313 \| 21.161 \| 0.02599 \| 19.779 \| 0.02769 \| 17.141 \| \| 0.00931 \| 42.486 \| 0.00986 \| 39.974 \| 0.01939 \| 34.786 \| 0.03 \| 29.042 \| 0.03145 \| 26.943 \| 0.03287 \| 23.712 \| \| 0.01188 \| 50.946 \| 0.01291 \| 45.458 \| 0.0269 \| 42.754 \| 0.03939 \| 37.315 \| 0.04064 \| 32.459 \| 0.0457 \| 29.735 \| \| 0.01313 \| 56.419 \| 0.01569 \| 52.356 \| 0.03893 \| 49.984 \| 0.05252 \| 45.353 \| 0.05643 \| 38.984 \| 0.05894 \| 34.832 \| \| 0.01563 \| 67.5 \| 0.01637 \| 55.237 \| 0.04768 \| 56.783 \| 0.06942 \| 52.721 \| 0.07345 \| 47.225 \| 0.07712 \| 38.511 \| \| 0.01752 \| 75.146 \| 0.01793 \| 60.455 \| 0.05903 \| 68.774 \| 0.08442 \| 59.859 \| 0.09033 \| 48.934 \| 0.09331 \| 41.856 \| \| 0.02314 \| 91.567 \| 0.02169 \| 70.884 \| 0.07398 \| 76.441 \| 0.1032 \| 68.616 \| 0.1094 \| 53.711 \| 0.1138 \| 44.486 \| \| 0.02502 \| 96.718 \| 0.02407 \| 78.431 \| 0.09491 \| 83.861 \| 0.1201 \| 75.817 \| 0.13431 \| 55.836 \| 0.1324 \| 46.567 \| \| 0.03379 \| 119.022 \| 0.0303 \| 89.301 \| 0.11035 \| 94.031 \| 0.15142 \| 82.026 \| 0.1488 \| 57.948 \| 0.16473 \| 52.996 \|  \| Perimeter pressure 120kPa \| \| \| \| \| \| \| \| \| \| \| \| \| --- \| --- \| --- \| --- \| --- \| --- \| --- \| --- \| --- \| --- \| --- \| --- \| \| dynamic stress/% \| Dynamic strain/KPa \| dynamic stress/% \| Dynamic strain/KPa \| dynamic stress/% \| Dynamic strain/KPa \| dynamic stress/% \| Dynamic strain/KPa \| dynamic stress/% \| Dynamic strain/KPa \| dynamic stress/% \| Dynamic strain/KPa \| \| 0.00561 \| 35.2302 \| 0.00688 \| 30.99258 \| 0.0078 \| 18.56614 \| 0.00874 \| 8.2462 \| 0.0105 \| 7.78948 \| 0.01199 \| 7.4982 \| \| 0.00674 \| 38.2652 \| 0.008 \| 34.02758 \| 0.00923 \| 21.60114 \| 0.01261 \| 14.7082 \| 0.01376 \| 12.25148 \| 0.01418 \| 10.9192 \| \| 0.00617 \| 43.12898 \| 0.00744 \| 38.89136 \| 0.01436 \| 26.46492 \| 0.02067 \| 24.41998 \| 0.02182 \| 21.96326 \| 0.02523 \| 20.39998 \| \| 0.00673 \| 43.7446 \| 0.008 \| 39.50698 \| 0.01792 \| 34.08054 \| 0.02742 \| 30.3006 \| 0.02857 \| 27.84388 \| 0.03029 \| 24.9706 \| \| 0.00932 \| 54.0585 \| 0.01058 \| 49.82088 \| 0.02351 \| 40.39444 \| 0.03682 \| 40.4275 \| 0.03798 \| 37.97078 \| 0.04313 \| 32.8475 \| \| 0.01057 \| 58.9725 \| 0.01184 \| 54.73488 \| 0.03476 \| 49.30844 \| 0.04996 \| 47.9065 \| 0.05111 \| 42.44978 \| 0.05638 \| 37.3855 \| \| 0.01204 \| 72.3677 \| 0.01331 \| 58.13008 \| 0.04923 \| 58.70364 \| 0.06583 \| 57.5887 \| 0.06698 \| 52.13198 \| 0.07353 \| 43.3787 \| \| 0.01496 \| 78.1343 \| 0.01623 \| 67.89668 \| 0.05215 \| 62.47024 \| 0.08186 \| 62.8473 \| 0.08301 \| 57.39058 \| 0.09075 \| 44.8443 \| \| 0.01961 \| 94.7894 \| 0.02088 \| 75.55178 \| 0.06881 \| 70.12534 \| 0.09968 \| 71.8384 \| 0.10083 \| 66.38168 \| 0.11028 \| 47.7084 \| \| 0.0238 \| 106.1736 \| 0.02506 \| 85.93598 \| 0.07399 \| 71.50954 \| 0.11888 \| 79.2726 \| 0.12003 \| 70.81588 \| 0.13118 \| 50.0226 \| \| 0.03057 \| 125.58863 \| 0.03184 \| 93.35101 \| 0.08076 \| 72.92457 \| 0.1482 \| 84.59263 \| 0.15015 \| 76.13591 \| 0.16151 \| 51.56263 \| \| \| |
| --- | --- | --- | --- | --- | --- | --- | --- | --- | --- | --- | --- | --- | --- | --- | --- | --- | --- | --- | --- | --- | --- | --- | --- | --- | --- | --- | --- | --- | --- | --- | --- | --- | --- | --- | --- | --- | --- | --- | --- | --- | --- | --- | --- | --- | --- | --- | --- | --- | --- | --- | --- | --- | --- | --- | --- | --- | --- | --- | --- | --- | --- | --- | --- | --- | --- | --- | --- | --- | --- | --- | --- | --- | --- | --- | --- | --- | --- | --- | --- | --- | --- | --- | --- | --- | --- | --- | --- | --- | --- | --- | --- | --- | --- | --- | --- | --- | --- | --- | --- | --- | --- | --- | --- | --- | --- | --- | --- | --- | --- | --- | --- | --- | --- | --- | --- | --- | --- | --- | --- | --- | --- | --- | --- | --- | --- | --- | --- | --- | --- | --- | --- | --- | --- | --- | --- | --- | --- | --- | --- | --- | --- | --- | --- | --- | --- | --- | --- | --- | --- | --- | --- | --- | --- | --- | --- | --- | --- | --- | --- | --- | --- | --- | --- | --- | --- | --- | --- | --- | --- | --- | --- | --- | --- | --- | --- | --- | --- | --- | --- | --- | --- | --- | --- | --- | --- | --- | --- | --- | --- | --- | --- | --- | --- | --- | --- | --- | --- | --- | --- | --- | --- | --- | --- | --- | --- | --- | --- | --- | --- | --- | --- | --- | --- | --- | --- | --- | --- | --- | --- | --- | --- | --- | --- | --- | --- | --- | --- | --- | --- | --- | --- | --- | --- | --- | --- | --- | --- | --- | --- | --- | --- | --- | --- | --- | --- | --- | --- | --- | --- | --- | --- | --- | --- | --- | --- | --- | --- | --- | --- | --- | --- | --- | --- | --- | --- | --- | --- | --- | --- | --- | --- | --- | --- | --- | --- | --- | --- | --- | --- | --- | --- | --- | --- | --- | --- | --- | --- | --- | --- | --- | --- | --- | --- | --- | --- | --- | --- | --- | --- | --- | --- | --- | --- | --- | --- | --- | --- | --- | --- | --- | --- | --- | --- | --- | --- | --- | --- | --- | --- | --- | --- | --- | --- | --- | --- | --- | --- | --- | --- | --- | --- | --- | --- | --- | --- | --- | --- | --- | --- | --- | --- | --- | --- | --- | --- | --- | --- | --- | --- | --- | --- | --- | --- | --- | --- | --- | --- | --- | --- | --- | --- | --- | --- | --- | --- | --- | --- | --- | --- | --- | --- | --- | --- | --- | --- | --- | --- | --- | --- | --- | --- | --- | --- | --- | --- | --- | --- | --- | --- | --- | --- | --- | --- | --- | --- | --- | --- | --- | --- | --- | --- | --- | --- | --- | --- | --- | --- | --- | --- | --- | --- | --- | --- | --- | --- | --- | --- | --- | --- | --- | --- | --- | --- | --- | --- | --- | --- | --- | --- | --- | --- | --- | --- | --- | --- | --- | --- | --- | --- | --- | --- | --- | --- | --- | --- | --- | --- | --- | --- | --- | --- | --- | --- | --- | --- | --- | --- | --- | --- | --- | --- | --- | --- | --- | --- | --- | --- | --- | --- | --- | --- | --- | --- | --- | --- | --- | --- | --- | --- | --- | --- | --- | --- | --- | --- | --- | --- | --- | --- | --- | --- | --- | --- | --- | --- | --- | --- | --- | --- | --- | --- | --- | --- | --- | --- | --- | --- | --- | --- | --- | --- | --- | --- | --- | --- | --- | --- | --- | --- | --- | --- | --- | --- | --- | --- | --- | --- | --- | --- | --- | --- | --- | --- | --- | --- | --- |

| Table .11 Fitting parameters of dynamic stress-strain curves of mixes under different dry and wet cycles | | | | | | | | |
| --- | --- | --- | --- | --- | --- | --- | --- | --- |
| Fitting equation | Peripheral pressure  /KPa | Number of dry and wet cycles/N | R^2^ |  | Fitting equation | Peripheral pressure  /KPa | Number of dry and wet cycles/N | R^2^ |
|  | 40 | 0 | 0.996 |  | $\sigma_{d}=a*\varepsilon_{d}^{b}$ | 80 | 3 | 0.979 |
| $\sigma_{d}=a*\varepsilon_{d}^{b}$ |  | 1 | 0.982 |  | $\sigma_{d}=\frac{\varepsilon_{d}}{a{\varepsilon_{d}}^{b}}$ |  | 4 | 0.935 |
|  |  | 2 | 0.989 |  |  |  | 5 | 0.940 |
| $\sigma_{d}=\frac{\varepsilon_{d}}{a{\varepsilon_{d}}^{b}}$ |  | 3 | 0.991 |  | $\sigma_{d}=a*\varepsilon_{d}^{b}$ | 120 | 0 | 0.989 |
|  |  | 4 | 0.976 |  |  |  | 1 | 0.971 |
|  |  | 5 | 0.991 |  |  |  | 2 | 0.990 |
| $\sigma_{d}=a*\varepsilon_{d}^{b}$ | 80 | 0 | 0.996 |  |  |  | 3 | 0.980 |
|  |  | 1 | 0.993 |  | $\sigma_{d}=\frac{\varepsilon_{d}}{a{\varepsilon_{d}}^{b}}$ |  | 4 | 0.972 |
|  |  | 2 | 0.991 |  |  |  | 5 | 0.924 |

Note: where $\sigma_{d}$ is the dynamic stress;$\varepsilon_{d}$ is the dynamic strain;

Table. 12 Error evaluation results of dynamic stress-strain model for mixes under different dry and wet cycles

| Number of dry and wet cycles/N  Peripheral pressure  /KPa | 40 | | 80 | | 120 | |
| --- | --- | --- | --- | --- | --- | --- |
|  | MAE | RMSE | MAE | RMSE | MAE | RMSE |
| 0 | 1.1886 | 1.4828 | 1.5649 | 1.7827 | 2.2551 | 2.9554 |
| 1 | 2.9719 | 3.9112 | 1.2313 | 1.5289 | 2.8563 | 3.4656 |
| 2 | 1.7403 | 2.4952 | 1.7593 | 2.2202 | 1.7507 | 1.9442 |
| 3 | 2.7948 | 3.2932 | 3.0594 | 3.5773 | 2.9098 | 3.4583 |
| 4 | 3.6036 | 4.1861 | 4.0065 | 4.5077 | 3.0684 | 3.7623 |
| 5 | 1.3359 | 1.9234 | 3.2552 | 3.7149 | 3.5237 | 4.1293 |

Table. 13 Values of fitted parameters for dynamic stress-strain curves of mixes with different perimeter pressures and different numbers of wet and dry cycles

| Pressurization/KPa | Number of dry and wet cycles /N | a | b |
| --- | --- | --- | --- |
| 40 | 0 | 1813.46 | 0.89906 |
|  | 1 | 200.445 | 0.35152 |
|  | 2 | 182.997 | 0.39547 |
|  | 3 | 131.907 | 0.43708 |
| 80 | 0 | 1864.55 | 0.80521 |
|  | 1 | 1471.16 | 0.77552 |
|  | 2 | 335.64 | 0.58697 |
|  | 3 | 325.02 | 0.65588 |
| 120 | 0 | 1515.70 | 0.7092 |
|  | 1 | 914.40 | 0.6177 |
|  | 2 | 305.60 | 0.5731 |
|  | 3 | 302.34 | 0.6345 |

Table. 14 Parameter a, b fitting relationship equation

| Pressurization/KPa | | Parameter a relational equation | R^2^ | Parameter b relational equation | R^2^ |
| --- | --- | --- | --- | --- | --- |
| 40 | a=1657.3*e^(-N/0.2784)+156.14 | | 0.999 | b=e^(-0.116-1.06*N+0.279*N^2^) | 0.989 |
| 80 | a=3274.1*e^(-N/3.979)-1366.4 | | 0.902 | b=0.714+0.1145*cos(78.242*pi*N/180) | 0.984 |
| 120 | a=1537.9*e^(-N/1.614)-3.2106 | | 0.968 | b=0.71216-0.14155*N+0.03823*N^2^ | 0.982 |

Note：N is the number of wet/dry cycles

Table.15 Modified eigenstructural model

| Pressurization/KPa | Modified eigenstructural model |
| --- | --- |
| 40 | $\sigma_{d}=（1657.3*e^(-N/0.2784)+156.14)*\varepsilon_{d}^{e^(-0.116-1.06*N+0.279*N^{2})}$ |
| 80 | $\sigma_{d}=（3274.1*e^(-N/3.979)-1366.4）*\varepsilon_{d}^{0.714+0.1145*cos(78.242*pi*N/180)}$ |
| 120 | $\sigma_{d}=（1537.9*e^(-N/1.614)-3.2106）*\varepsilon_{d}^{0.71216-0.14155*N+0.03823*N^{2}}$ |

Note：N is the number of wet/dry cycles

| 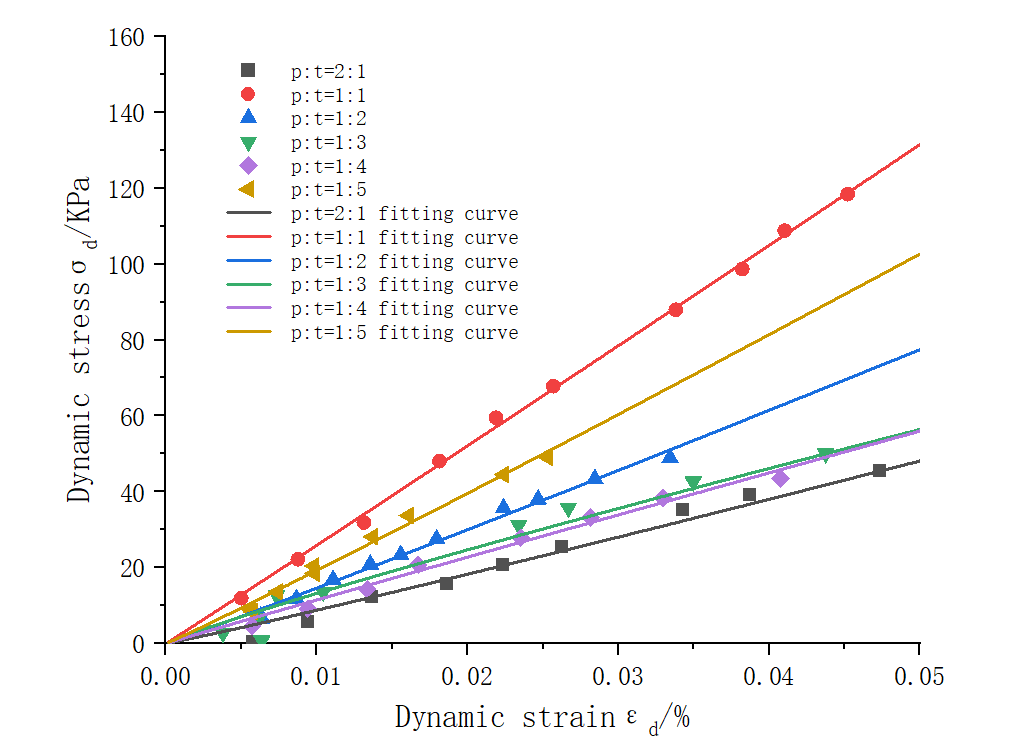 | 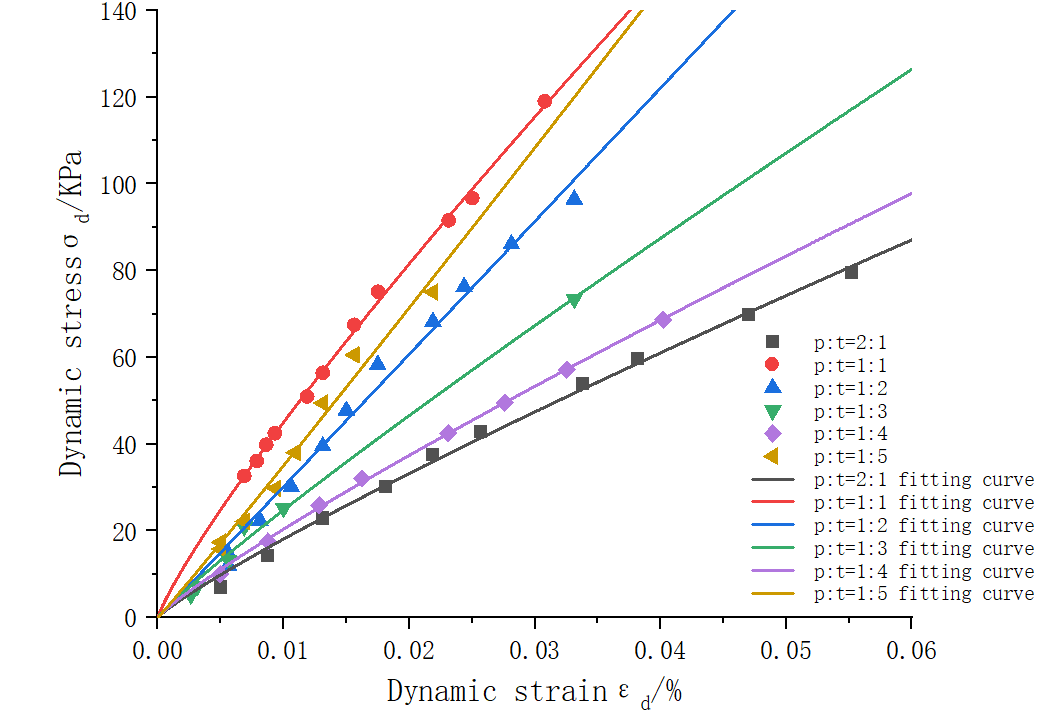 |
| --- | --- |
| **4% cement, Perimeter pressure 80kPa, consolidation ratio 1.5** | **6% cement, Perimeter pressure 80kPa, consolidation ratio 1.5** |
| 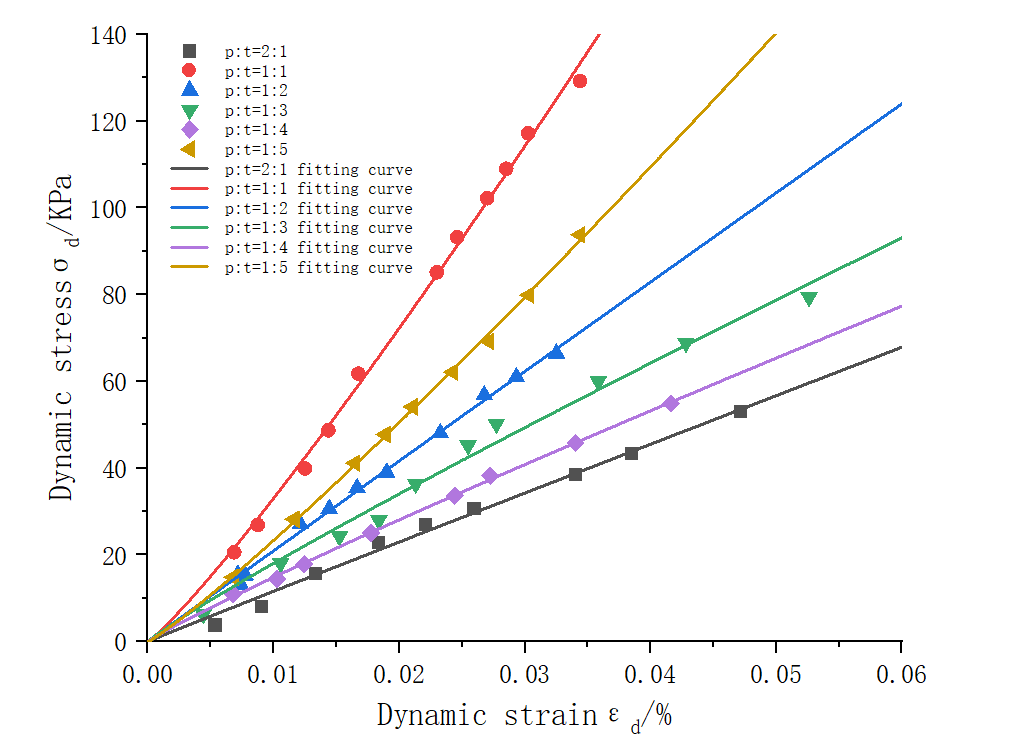 | 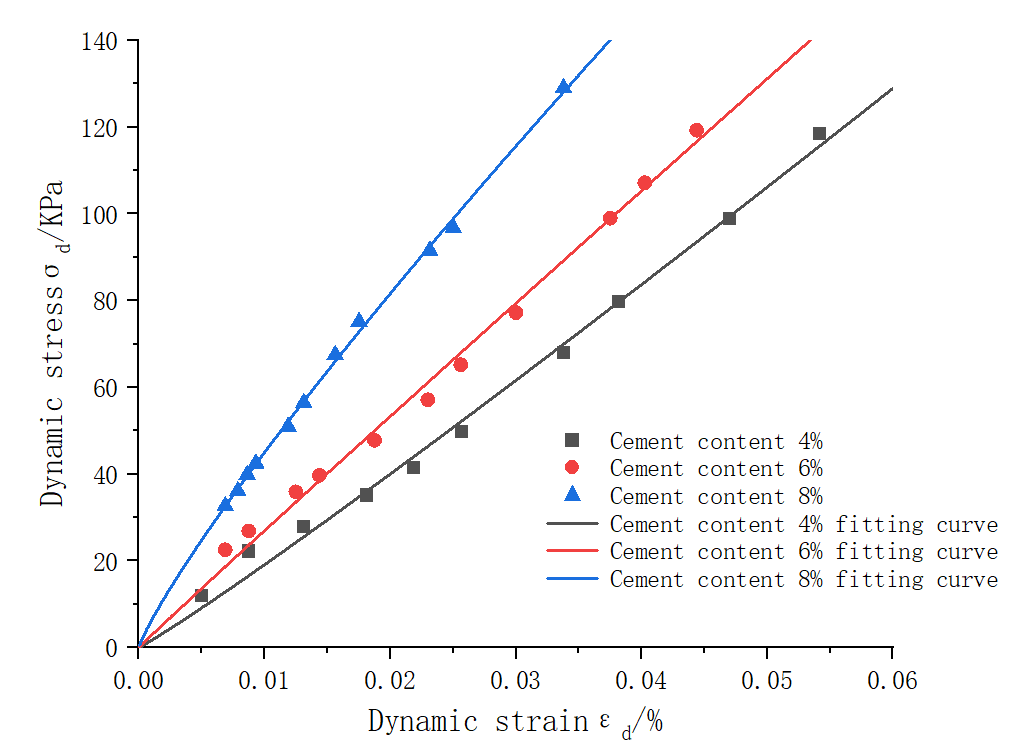 |
| **8% cement, Perimeter pressure 80kPa, consolidation ratio 1.5** | **P:T=1:1 Perimeter pressure 80kPa, consolidation ratio 1.5** |
| Fig. 7 Dynamic stress-strain curves of mixes with different mix ratios under 0 dry and wet cycles | |
| **Raw data for Figure 7**   \| C=4% \| \| \| \| \| \| \| \| \| \| \| \| \| --- \| --- \| --- \| --- \| --- \| --- \| --- \| --- \| --- \| --- \| --- \| --- \| \| dynamic stress/% \| Dynamic strain/KPa \| dynamic stress/% \| Dynamic strain/KPa \| dynamic stress/% \| Dynamic strain/KPa \| dynamic stress/% \| Dynamic strain/KPa \| dynamic stress/% \| Dynamic strain/KPa \| dynamic stress/% \| Dynamic strain/KPa \| \| 0.00575 \| 0.534 \| 0.005 \| 11.969 \| 0.00637 \| 6.622 \| 0.00637 \| 0.795 \| 0.00572 \| 4.534 \| 0.00574 \| 8.373 \| \| 0.00941 \| 5.731 \| 0.00876 \| 22.181 \| 0.00628 \| 7.152 \| 0.00378 \| 2.769 \| 0.00941 \| 9.131 \| 0.00565 \| 8.907 \| \| 0.01366 \| 12.327 \| 0.01313 \| 31.887 \| 0.00866 \| 11.755 \| 0.00617 \| 6.714 \| 0.01336 \| 14.327 \| 0.00741 \| 13.587 \| \| 0.01862 \| 15.808 \| 0.01815 \| 48.058 \| 0.01109 \| 16.861 \| 0.00734 \| 12.727 \| 0.01672 \| 20.808 \| 0.00984 \| 18.528 \| \| 0.02234 \| 20.851 \| 0.0219 \| 59.491 \| 0.01356 \| 20.9 \| 0.01044 \| 13.607 \| 0.02354 \| 27.851 \| 0.00981 \| 20.408 \| \| 0.02626 \| 25.504 \| 0.02569 \| 67.824 \| 0.01557 \| 23.423 \| 0.0234 \| 31.215 \| 0.02816 \| 33.204 \| 0.0137 \| 28.146 \| \| 0.03427 \| 35.172 \| 0.03382 \| 87.952 \| 0.01795 \| 27.55 \| 0.02669 \| 35.723 \| 0.03297 \| 38.372 \| 0.01608 \| 33.753 \| \| 0.03875 \| 39.154 \| 0.03822 \| 98.675 \| 0.0224 \| 35.724 \| 0.03497 \| 42.641 \| 0.04075 \| 43.54 \| 0.0224 \| 44.53 \| \| 0.04735 \| 45.593 \| 0.04103 \| 108.823 \| 0.02469 \| 38.054 \| 0.04375 \| 50.0879 \|  \|  \| 0.02531 \| 49.048 \| \| 0.05554 \| 50.761 \| 0.04522 \| 118.431 \| 0.02845 \| 43.45 \| 0.05175 \| 54.831 \|  \|  \|  \|  \| \|  \|  \|  \|  \| 0.03344 \| 48.899 \|  \|  \|  \|  \|  \|  \|  \| C=6% \| \| \| \| \| \| \| \| \| \| \| \| \| --- \| --- \| --- \| --- \| --- \| --- \| --- \| --- \| --- \| --- \| --- \| --- \| \| dynamic stress/% \| Dynamic strain/KPa \| dynamic stress/% \| Dynamic strain/KPa \| dynamic stress/% \| Dynamic strain/KPa \| dynamic stress/% \| Dynamic strain/KPa \| dynamic stress/% \| Dynamic strain/KPa \| dynamic stress/% \| Dynamic strain/KPa \| \| 0.005 \| 6.969 \| 0.00687 \| 32.645 \| 0.00562 \| 12.057 \| 0.00263 \| 5.23 \| 0.00498 \| 9.969 \| 0.005 \| 15.808 \| \| 0.00876 \| 14.181 \| 0.00788 \| 36.107 \| 0.00563 \| 14.602 \| 0.00313 \| 7.219 \| 0.00876 \| 17.581 \| 0.005 \| 17.357 \| \| 0.01313 \| 22.887 \| 0.00863 \| 39.87 \| 0.00813 \| 22.315 \| 0.00563 \| 13.274 \| 0.01283 \| 25.887 \| 0.00688 \| 22.147 \| \| 0.01815 \| 30.058 \| 0.00931 \| 42.486 \| 0.01062 \| 30.111 \| 0.00688 \| 20.977 \| 0.01625 \| 32.058 \| 0.00938 \| 29.778 \| \| 0.0219 \| 37.491 \| 0.01188 \| 50.946 \| 0.01313 \| 39.54 \| 0.01 \| 25.247 \| 0.0231 \| 42.491 \| 0.01094 \| 38.048 \| \| 0.02569 \| 42.824 \| 0.01313 \| 56.419 \| 0.015 \| 47.743 \| 0.03314 \| 73.419 \| 0.02759 \| 49.524 \| 0.01313 \| 49.466 \| \| 0.03382 \| 53.952 \| 0.01563 \| 67.5 \| 0.0175 \| 58.33 \|  \|  \| 0.03252 \| 57.152 \| 0.01563 \| 60.533 \| \| 0.03822 \| 59.675 \| 0.01752 \| 75.146 \| 0.02188 \| 68.245 \|  \|  \| 0.04022 \| 68.675 \| 0.02188 \| 75.051 \| \| 0.04703 \| 69.823 \| 0.02314 \| 91.567 \| 0.02438 \| 76.284 \|  \|  \|  \|  \| -- \| -- \| | |

| C=8% | | | | | | | | | | | |
| --- | --- | --- | --- | --- | --- | --- | --- | --- | --- | --- | --- |
| dynamic stress/% | Dynamic strain/KPa | dynamic stress/% | Dynamic strain/KPa | dynamic stress/% | Dynamic strain/KPa | dynamic stress/% | Dynamic strain/KPa | dynamic stress/% | Dynamic strain/KPa | dynamic stress/% | Dynamic strain/KPa |
| 0.00537 | 3.744 | 0.00687 | 20.563 | 0.00741 | 13.08 | 0.00441 | 6.253 | 0.00677 | 10.992 | 0.00679 | 14.831 |
| 0.00908 | 8.061 | 0.00875 | 26.86 | 0.00715 | 15.462 | 0.01057 | 18.079 | 0.01028 | 14.441 | 0.01165 | 28.217 |
| 0.01339 | 15.677 | 0.0125 | 39.887 | 0.00774 | 15.295 | 0.01524 | 24.254 | 0.01244 | 17.867 | 0.01649 | 41.127 |
| 0.01839 | 22.808 | 0.01437 | 48.688 | 0.01214 | 27.071 | 0.01839 | 27.937 | 0.01776 | 25.018 | 0.01889 | 47.738 |
| 0.02212 | 26.988 | 0.01676 | 61.764 | 0.01443 | 30.638 | 0.02131 | 36.345 | 0.02441 | 33.589 | 0.02107 | 54.146 |
| 0.02598 | 30.641 | 0.023 | 85.126 | 0.01665 | 35.429 | 0.02548 | 45.221 | 0.02724 | 38.21 | 0.02428 | 62.152 |
| 0.03405 | 38.522 | 0.02462 | 93.222 | 0.019 | 39.001 | 0.02774 | 50.174 | 0.03402 | 45.823 | 0.02713 | 69.204 |
| 0.03849 | 43.291 | 0.027 | 102.232 | 0.02327 | 48.077 | 0.03585 | 59.994 | 0.04162 | 54.893 | 0.03033 | 79.883 |
| 0.04719 | 52.943 | 0.02851 | 108.994 | 0.02677 | 56.818 | 0.04282 | 68.8519 |  |  | 0.03439 | 93.812 |
|  |  | 0.03026 | 117.179 | 0.02933 | 61.001 | 0.05262 | 79.382 |  |  |  |  |
|  |  | 0.03439 | 129.256 | 0.03251 | 66.449 |  |  |  |  |  |  |

| P:T=1:1 Perimeter pressure 80kPa, consolidation ratio 1.5 | | | | | |
| --- | --- | --- | --- | --- | --- |
| dynamic stress/% | Dynamic strain/KPa | dynamic stress/% | Dynamic strain/KPa | dynamic stress/% | Dynamic strain/KPa |
| 0.005 | 11.969 | 0.00687 | 22.563 | 0.00687 | 32.645 |
| 0.00876 | 22.181 | 0.00875 | 26.86 | 0.00788 | 36.107 |
| 0.01313 | 27.887 | 0.0125 | 35.887 | 0.00863 | 39.87 |
| 0.01815 | 35.058 | 0.01437 | 39.688 | 0.00931 | 42.486 |
| 0.0219 | 41.491 | 0.01876 | 47.764 | 0.01188 | 50.946 |
| 0.02569 | 49.824 | 0.023 | 57.126 | 0.01313 | 56.419 |
| 0.03382 | 67.952 | 0.02562 | 65.222 | 0.01563 | 67.5 |
| 0.03822 | 79.675 | 0.03 | 77.232 | 0.01752 | 75.146 |
| 0.04703 | 98.823 | 0.03751 | 98.994 | 0.02314 | 91.567 |
| 0.05422 | 118.431 | 0.04026 | 107.179 | 0.02502 | 96.718 |
|  |  | 0.04439 | 119.256 | 0.03379 | 129.022 |

| Table. 16 Fitting parameters of dynamic stress-strain curves of lower mixes with different ratios | | | |  |
| --- | --- | --- | --- | --- |
| Fitting equation | Cement content | Peripheral pressure  /KPa consolidation ratio | R^2^min | |
| $\sigma_{d}=a*\varepsilon_{d}^{b}$ | 4% | 80;1.5 | 0.985 | |
|  | 6% |  | 0.978 | |
|  | 8% |  | 0.985 | |

Note: where $\sigma_{d}$ is the dynamic stress;$\varepsilon_{d}$ is the dynamic strain;

Table.17 Levels of factors in orthogonal test

| Level | Pressurization/KPa | Consolidation ratio | Number of dry and wet cycles /N |
| --- | --- | --- | --- |
| 1 | 40 | 1 | 0 |
| 2 | 80 | 1.5 | 3 |
| 3 | 120 | 2 | 5 |

Table.18 Table of orthogonal tests

| Test number | Pressurization/KPa | Consolidation ratio | Number of dry and wet cycles /N | Maximum dynamic stress $\sigma_{d}$/kPa |
| --- | --- | --- | --- | --- |
| 1 | 1 | 1 | 1 | 218.9924 |
| 2 | 1 | 2 | 2 | 183.7487 |
| 3 | 1 | 3 | 3 | 192.505 |
| 4 | 2 | 1 | 2 | 215.118 |
| 5 | 2 | 2 | 3 | 211.5 |
| 6 | 2 | 3 | 1 | 387.6306 |
| 7 | 3 | 1 | 3 | 258.176 |
| 8 | 3 | 2 | 2 | 329.9323 |
| 9 | 3 | 3 | 1 | 451.6886 |

Table.19 Orthogonal test results of cement:phosphogypsum:red clay=6:47:47 mixes

| Independent variable | Degrees of freedom | Mean square | F | P | $R^{2}$ |
| --- | --- | --- | --- | --- | --- |
| Number of dry and wet cycles /N | 2 | 8295.123 | 149.316 | 0.007** | 0.998 |
| Pressurization/KPa | 2 | 16469.975 | 296.467 | 0.003** |  |
| Consolidation ratio | 2 | 4987.46 | 89.777 | 0.011* |  |

Note：* p<0.05 ** p<0.01

| 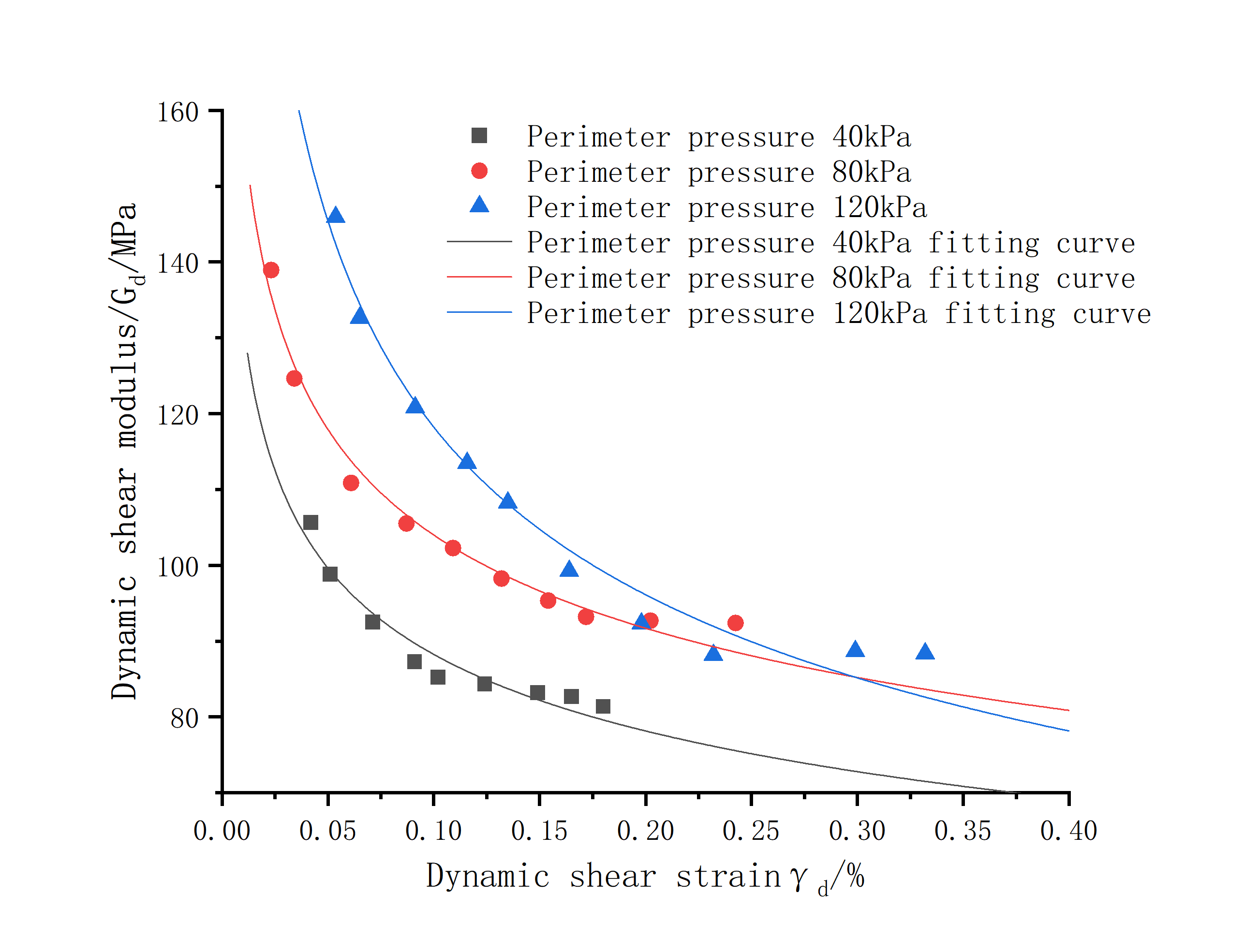 | 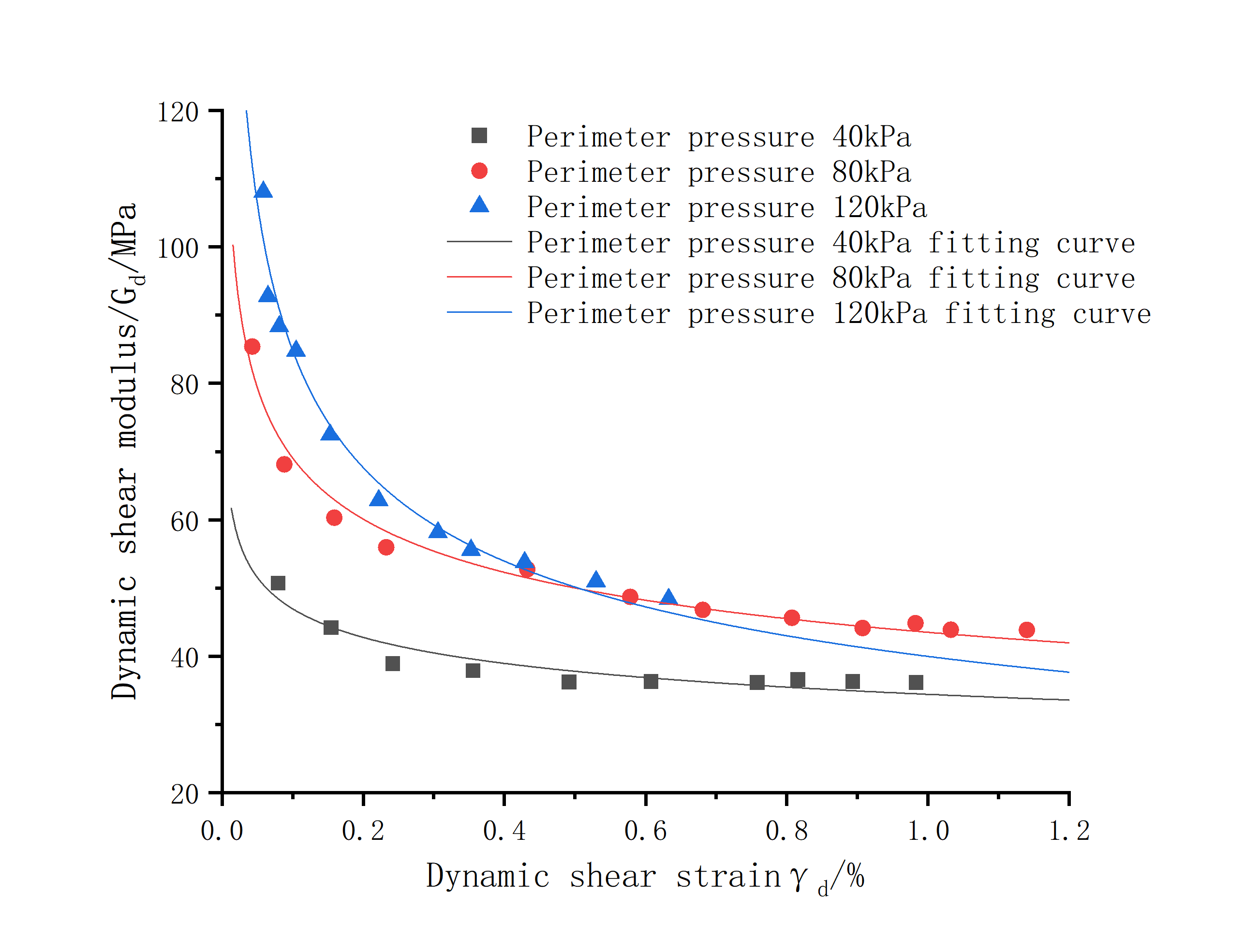 | |
| --- | --- | --- |
| **（a）0 wet/dry cycles** | **（b）3 wet/dry cycles** | |
| 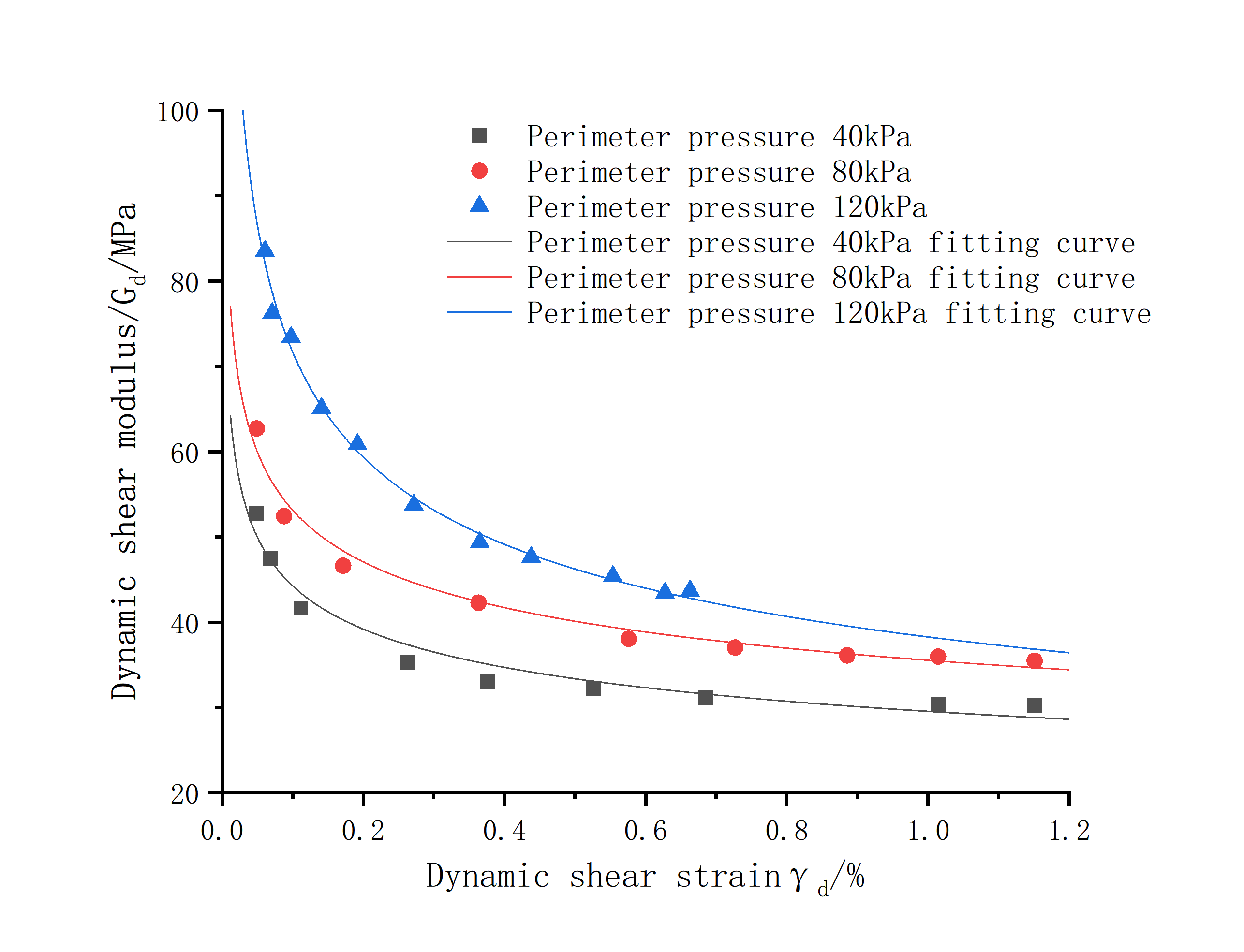 | | |
| **（c）5 wet/dry cycles** | | |
| Fig. 8 Dynamic shear modulus-dynamic shear strain curves of cement:phosphogypsum:red clay=6:47:47 mixes with different enclosing pressures, consolidation ratio 1.5   \| **Raw data for Figure 8** \| \| --- \|  \| **0 wet/dry cycles** \|  \|  \|  \|  \|  \| \| --- \| --- \| --- \| --- \| --- \| --- \| \| dynamic shear strain/% \| dynamic shear modulus/MPa \| dynamic shear strain/% \| dynamic shear modulus/MPa \| dynamic shear strain/% \| dynamic shear modulus/MPa \| \| 0.042 \| 105.65298 \| 0.02321 \| 138.93951 \| 0.0537 \| 145.93951 \| \| 0.051 \| 98.84925 \| 0.0342 \| 124.65298 \| 0.065 \| 132.65298 \| \| 0.0712 \| 92.51576 \| 0.061 \| 110.84925 \| 0.0912 \| 120.84925 \| \| 0.091 \| 87.28219 \| 0.0871 \| 105.51576 \| 0.1157 \| 113.51576 \| \| 0.102 \| 85.25742 \| 0.1091 \| 102.28219 \| 0.135 \| 108.28219 \| \| 0.124 \| 84.3419 \| 0.132 \| 98.25742 \| 0.164 \| 99.25742 \| \| 0.149 \| 83.20076 \| 0.154 \| 95.3419 \| 0.198 \| 92.3419 \| \| 0.165 \| 82.69131 \| 0.1719 \| 93.20076 \| 0.232 \| 88.20076 \| \| 0.18 \| 81.38464 \| 0.20236 \| 92.69131 \| 0.299 \| 88.69131 \| \|  \|  \| 0.24252 \| 92.38464 \| 0.332 \| 88.38464 \| \| **3 wet/dry cycles** \|  \|  \|  \|  \|  \| \| dynamic shear strain/% \| dynamic shear modulus/MPa \| dynamic shear strain/% \| dynamic shear modulus/MPa \| dynamic shear strain/% \| dynamic shear modulus/MPa \| \| 0.0795 \| 50.70287 \| 0.043 \| 85.39573 \| 0.0585 \| 108.05029 \| \| 0.1545 \| 44.19976 \| 0.08825 \| 68.10921 \| 0.0651 \| 92.76376 \| \| 0.242 \| 38.92184 \| 0.15925 \| 60.30548 \| 0.0813 \| 88.35205 \| \| 0.35575 \| 37.89388 \| 0.23275 \| 55.97199 \| 0.105 \| 84.75185 \| \| 0.492 \| 36.20657 \| 0.4325 \| 52.73842 \| 0.153 \| 72.47156 \| \| 0.60775 \| 36.27697 \| 0.57825 \| 48.71365 \| 0.222 \| 62.84185 \| \| 0.75825 \| 36.15935 \| 0.681 \| 46.79813 \| 0.306 \| 58.14322 \| \| 0.8155 \| 36.56813 \| 0.8075 \| 45.65699 \| 0.3526 \| 55.57385 \| \| 0.8935 \| 36.31258 \| 0.9075 \| 44.14754 \| 0.4287 \| 53.76251 \| \| 0.98325 \| 36.17582 \| 0.9825 \| 44.84087 \| 0.53 \| 50.9945 \| \|  \|  \| 1.0325 \| 43.89 \| 0.6327 \| 48.39757 \| \|  \|  \| 1.14 \| 43.87 \|  \|  \| \| **5wet/dry cycles** \|  \|  \|  \|  \|  \| \| dynamic shear strain/% \| dynamic shear modulus/MPa \| dynamic shear strain/% \| dynamic shear modulus/MPa \| dynamic shear strain/% \| dynamic shear modulus/MPa \| \| 0.0493 \| 52.70417 \| 0.0493 \| 62.70417 \| 0.0611 \| 83.50518 \| \| 0.0681 \| 47.41764 \| 0.0881 \| 52.41764 \| 0.071 \| 76.21866 \| \| 0.1117 \| 41.61392 \| 0.1717 \| 46.61392 \| 0.0978 \| 73.41493 \| \| 0.2633 \| 35.28042 \| 0.3633 \| 42.28042 \| 0.1411 \| 65.08143 \| \| 0.376 \| 33.04685 \| 0.576 \| 38.04685 \| 0.192 \| 60.84787 \| \| 0.5266 \| 32.22209 \| 0.7266 \| 37.02209 \| 0.2717 \| 53.73465 \| \| 0.6856 \| 31.10656 \| 0.8856 \| 36.10656 \| 0.3652 \| 49.36137 \| \| 1.0143 \| 30.36542 \| 1.0143 \| 35.96542 \| 0.4381 \| 47.64966 \| \| 1.1511 \| 30.25597 \| 1.1511 \| 35.45597 \| 0.5537 \| 45.38549 \| \| 1.472 \| 30.1493 \| 1.472 \| 35.1493 \| 0.6274 \| 43.42548 \| \|  \|  \|  \|  \| 0.6631 \| 43.6793 \|   Table. 20 Dynamic shear modulus-dynamic shear strain fitting parameters under different  enclosure Pressures   \| Fitting equation \| Number of wet and dry cycles/N \| Pressurization/KPa \| a \| b \| R^2^ \| MAE \| RMSE \|  \|  \| \| --- \| --- \| --- \| --- \| --- \| --- \| --- \| --- \| --- \| --- \| \| $G_{d}=\frac{1}{a{\gamma_{d}}^{b}}$ \| 0 \| 40 \| 0.01696 \| 0.17528 \| 0.944 \| 1.651 \| 1.856 \|  \|  \| \| 80 \| 0.0146 \| 0.18133 \| 0.980 \| 1.666 \| 2.056 \|  \|  \| \| 120 \| 0.01682 \| 0.29856 \| 0.973 \| 2.635 \| 3.162 \|  \|  \| \| 3 \| 40 \| 0.02904 \| 0.13482 \| 0.901 \| 1.365 \| 1.579 \|  \|  \| \| 80 \| 0.02297 \| 0.20055 \| 0.978 \| 1.372 \| 1.809 \|  \|  \| \| 120 \| 0.02501 \| 0.32668 \| 0.976 \| 2.322 \| 2.968 \|  \|  \| \| 5 \| 40 \| 0.33383 \| 0.17535 \| 0.951 \| 1.583 \| 1.763 \|  \|  \| \| 80 \| 0.02814 \| 0.17472 \| 0.974 \| 1.142 \| 1.395 \|  \|  \| \| 120 \| 0.02613 \| 0.2726 \| 0.993 \| 0.888 \| 1.113 \|  \|  \| | | |
| 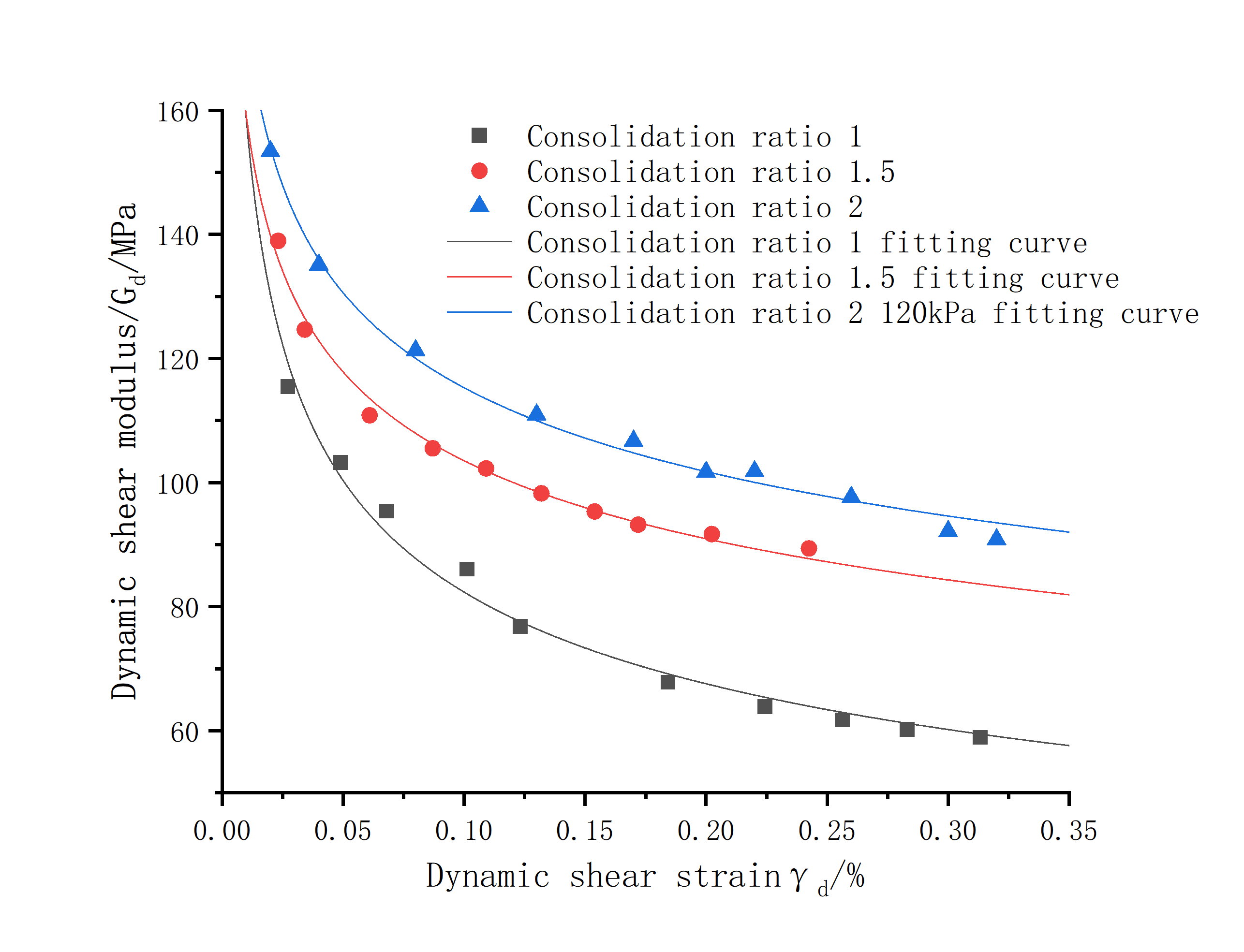 | | 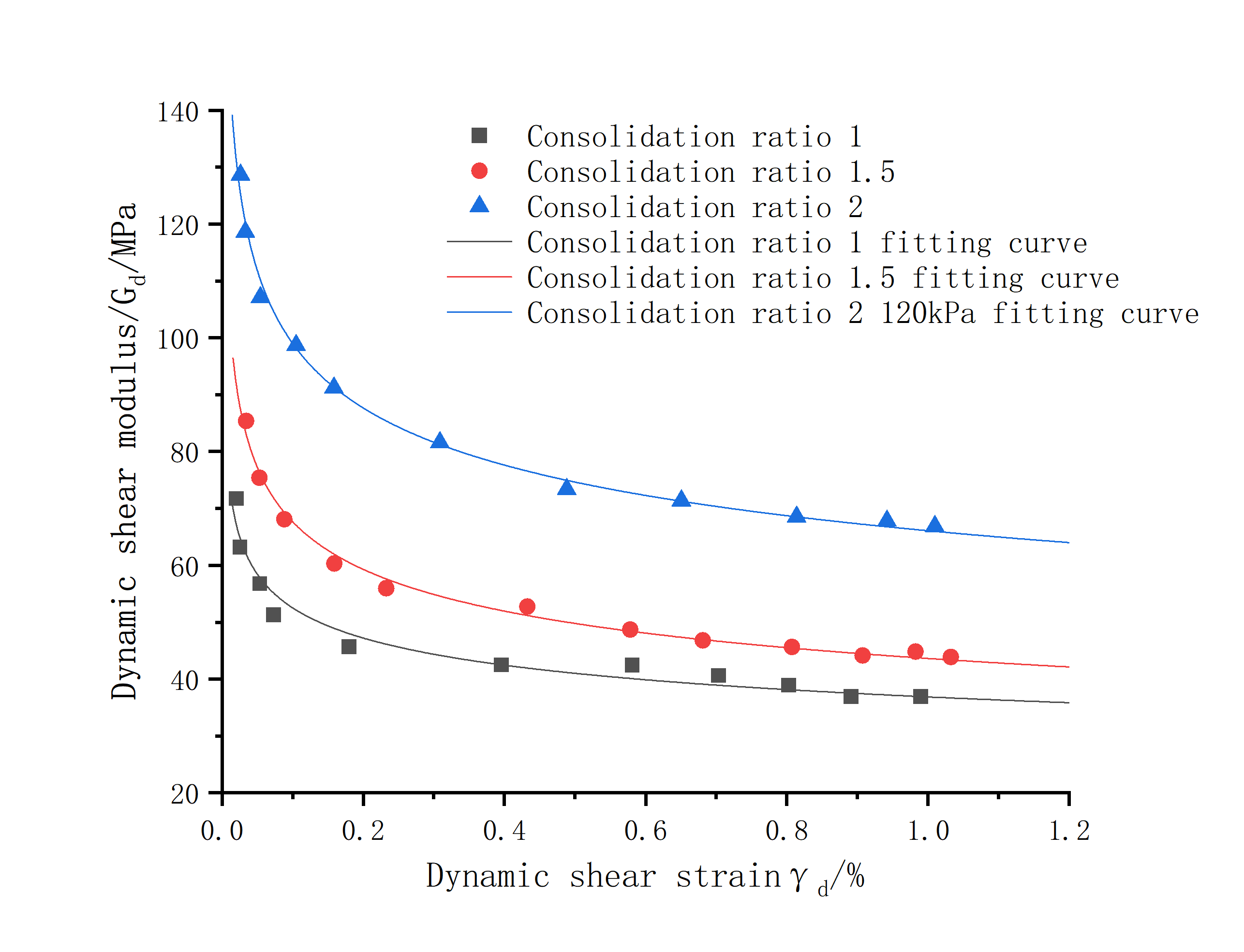 |
| **（a）0 wet/dry cycles** | | **（b）3 wet/dry cycles** |
| 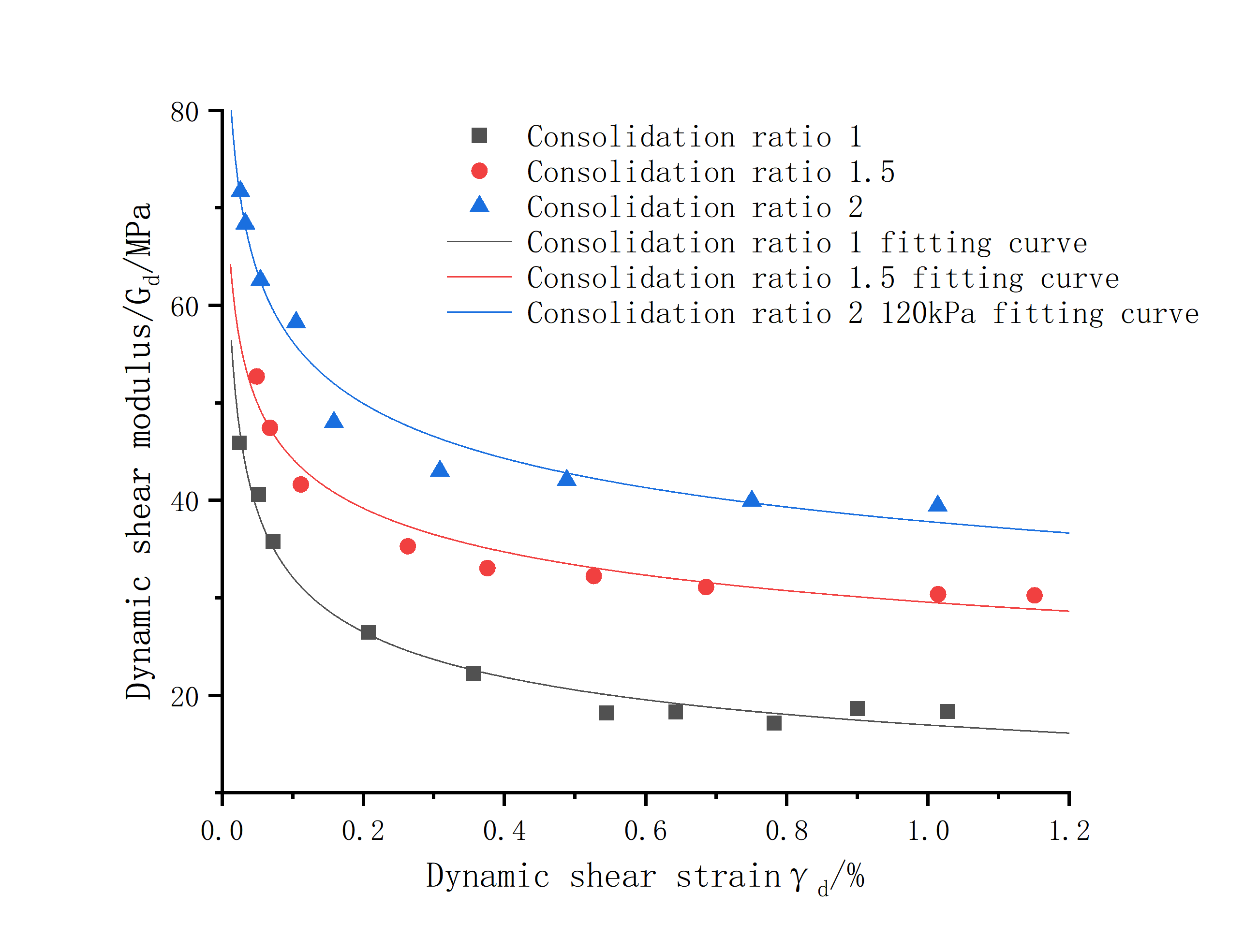 | | |
| **（c）5 wet/dry cycles** | | |
| Fig. 9 Dynamic shear modulus-dynamic shear strain curves of cement:phosphogypsum:red clay=6:47:47 mixes with different consolidation ratios, perimeter pressure 80KPa  **Raw data for Figure 9**   \| **0 wet/dry cycles** \|  \|  \|  \|  \|  \| \| --- \| --- \| --- \| --- \| --- \| --- \| \| dynamic shear strain/% \| dynamic shear modulus/MPa \| dynamic shear strain/% \| dynamic shear modulus/MPa \| dynamic shear strain/% \| dynamic shear modulus/MPa \| \| 0.0491 \| 103.19836 \| 0.0342 \| 124.65298 \| 0.04 \| 135.11062 \| \| 0.0681 \| 95.39463 \| 0.061 \| 110.84925 \| 0.08 \| 121.30689 \| \| 0.1013 \| 86.06113 \| 0.0871 \| 105.51576 \| 0.13 \| 110.97339 \| \| 0.1232 \| 76.82757 \| 0.1091 \| 102.28219 \| 0.17 \| 106.73983 \| \| 0.1843 \| 67.8028 \| 0.132 \| 98.25742 \| 0.2 \| 101.71506 \| \| 0.2243 \| 63.88728 \| 0.154 \| 95.3419 \| 0.22 \| 101.79954 \| \| 0.2563 \| 61.74614 \| 0.1719 \| 93.20076 \| 0.26 \| 97.6584 \| \| 0.2831 \| 60.23669 \| 0.20236 \| 91.69131 \| 0.3 \| 92.14894 \| \| 0.3133 \| 58.93002 \| 0.24252 \| 89.38464 \| 0.32 \| 90.84228 \| \| **3 wet/dry cycles** \|  \|  \|  \|  \|  \| \| dynamic shear strain/% \| dynamic shear modulus/MPa \| dynamic shear strain/% \| dynamic shear modulus/MPa \| dynamic shear strain/% \| dynamic shear modulus/MPa \| \| 0.02543 \| 63.19836 \| 0.053 \| 75.39573 \| 0.0331 \| 118.64714 \| \| 0.0536 \| 56.79463 \| 0.08825 \| 68.10921 \| 0.0545 \| 107.11062 \| \| 0.0732 \| 51.32113 \| 0.15925 \| 60.30548 \| 0.1051 \| 98.70689 \| \| 0.18 \| 45.70757 \| 0.23275 \| 55.97199 \| 0.1587 \| 91.23339 \| \| 0.396 \| 42.4828 \| 0.4325 \| 52.73842 \| 0.3088 \| 81.61983 \| \| 0.5811 \| 42.46728 \| 0.57825 \| 48.71365 \| 0.4882 \| 73.39506 \| \| 0.7035 \| 40.62614 \| 0.681 \| 46.79813 \| 0.6506 \| 71.37954 \| \| 0.8028 \| 38.90669 \| 0.8075 \| 45.65699 \| 0.8139 \| 68.5384 \| \| 0.8913 \| 36.96002 \| 0.9075 \| 44.14754 \| 0.942 \| 67.81894 \| \| 0.9897 \| 36.96002 \| 0.9825 \| 44.84087 \| 1.0098 \| 66.87228 \| \|  \|  \| 1.0325 \| 43.89 \|  \|  \| \| **5wet/dry cycles** \|  \|  \|  \|  \|  \| \| dynamic shear strain/% \| dynamic shear modulus/MPa \| dynamic shear strain/% \| dynamic shear modulus/MPa \| dynamic shear strain/% \| dynamic shear modulus/MPa \| \| 0.0518 \| 40.58822 \| 0.0681 \| 47.41764 \| 0.0331 \| 68.39828 \| \| 0.0721 \| 35.78449 \| 0.1117 \| 41.61392 \| 0.0545 \| 62.59455 \| \| 0.2075 \| 26.451 \| 0.2633 \| 35.28042 \| 0.1051 \| 58.26105 \| \| 0.3569 \| 22.21743 \| 0.376 \| 33.04685 \| 0.1587 \| 48.02749 \| \| 0.5444 \| 18.19266 \| 0.5266 \| 32.22209 \| 0.3088 \| 43.00272 \| \| 0.6428 \| 18.27714 \| 0.6856 \| 31.10656 \| 0.4882 \| 42.0872 \| \| 0.78193 \| 17.136 \| 1.0143 \| 30.36542 \| 0.7506 \| 39.94606 \| \| 0.9 \| 18.62655 \| 1.1511 \| 30.25597 \| 1.0139 \| 39.43661 \| \| 1.0278 \| 18.31988 \| 1.472 \| 30.1493 \| 1.242 \| 39.12994 \|   Table. 21 Results of dynamic shear modulus-dynamic shear strain fitting parameters and model error assessment under different consolidation ratios   \| Fitting equation \| Number of wet and dry cycles/N \| Consolidation ratio \| a \| b \| R^2^ \| MAE \| RMSE \| \| --- \| --- \| --- \| --- \| --- \| --- \| --- \| --- \| \| $G_{d}=\frac{1}{a{\gamma_{d}}^{b}}$ \| 0 \| 1 \| 0.02342 \| 0.28522 \| 0.985 \| 2.009 \| 2.376 \| \| 1.5 \| 0.01485 \| 0.18687 \| 0.989 \| 1.182 \| 1.541 \| \| 2 \| 0.01313 \| 0.17991 \| 0.993 \| 1.321 \| 1.557 \| \| 3 \| 1 \| 0.02713 \| 0.15383 \| 0.948 \| 1.720 \| 2.231 \| \| 1.5 \| 0.02302 \| 0.19228 \| 0.992 \| 1.018 \| 1.218 \| \| 2 \| 0.01518 \| 0.17664 \| 0.994 \| 1.230 \| 1.619 \| \| 5 \| 1 \| 0.05897 \| 0.27756 \| 0.985 \| 1.108 \| 1.251 \| \| 1.5 \| 0.03383 \| 0.17536 \| 0.951 \| 1.473 \| 1.691 \| \| 2 \| 0.02644 \| 0.17261 \| 0.970 \| 1.625 \| 2.101 \| | | |
| \| 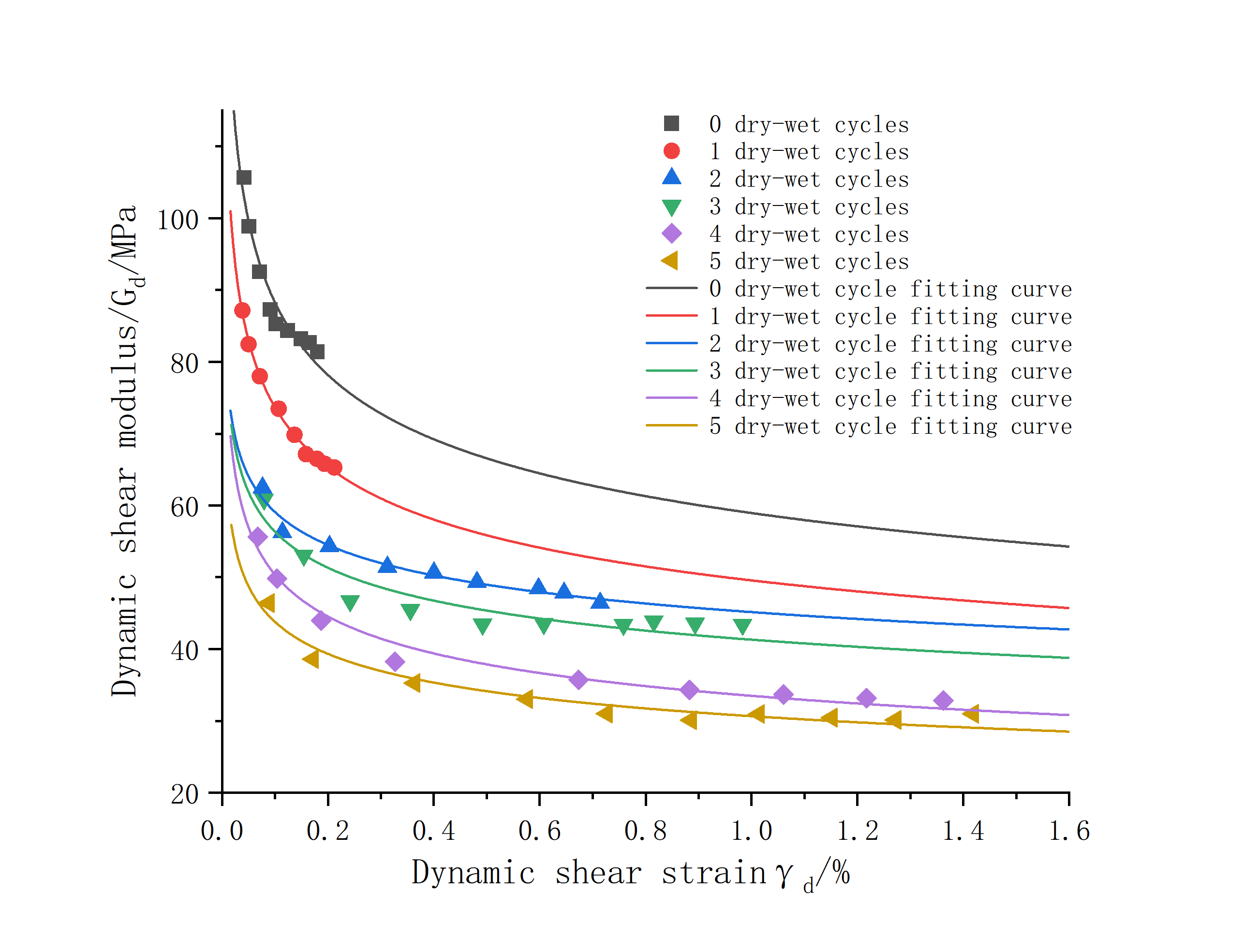 \| \| \| 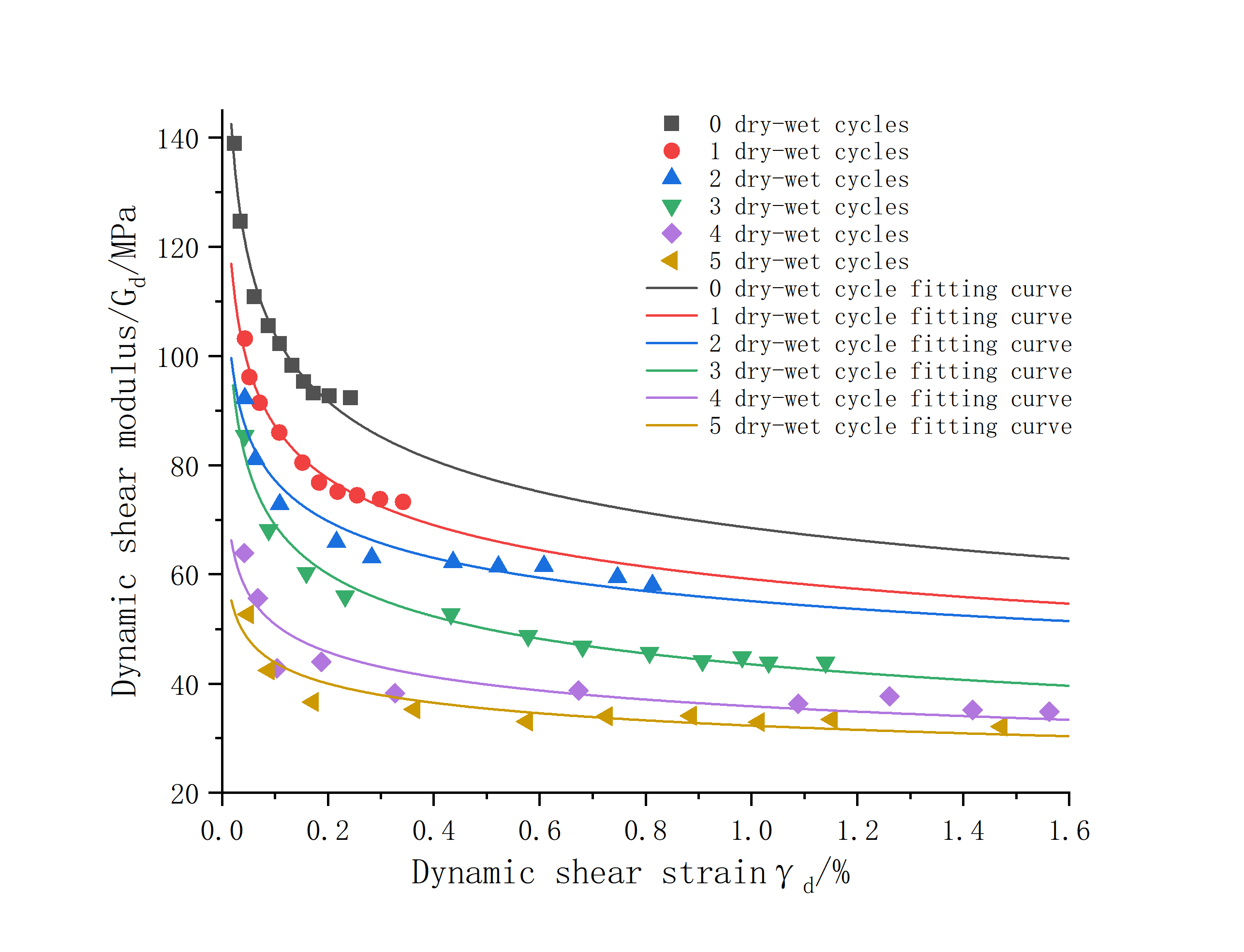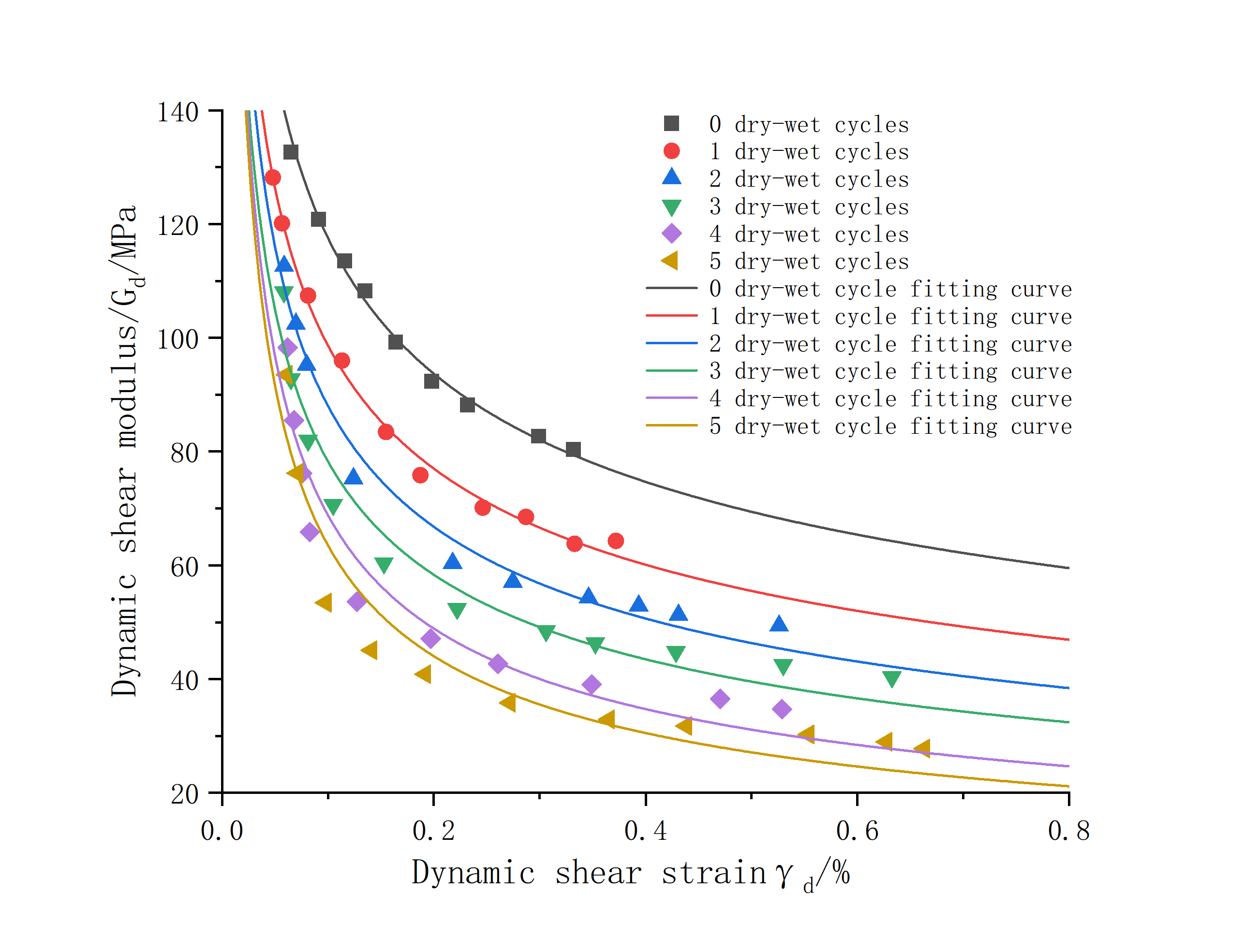 \| \| \| \| \| \| \| \| --- \| --- \| --- \| --- \| --- \| --- \| --- \| --- \| --- \| --- \| \| **(a) Perimeter pressure 40kPa** \| \| \| **(b) Perimeter pressure 80kPa (c) Perimeter pressure 120kPa** \| \| \| \| \| \| \| \| Fig. 10 Dynamic shear modulus-dynamic shear strain curves for cement:phosphogypsum:red clay=6:47:47 mixes with different number of wet and dry cycles, consolidation ratio 1.5  **Raw data for Figure 10**   \| **Perimeter pressure 40kPa** \| \| \| \| \| \| \| \| \| \| \| \| \| --- \| --- \| --- \| --- \| --- \| --- \| --- \| --- \| --- \| --- \| --- \| --- \| \| dynamic shear strain/% \| dynamic shear modulus/MPa \| dynamic shear strain/% \| dynamic shear modulus/MPa \| dynamic shear strain/% \| dynamic shear modulus/MPa \| dynamic shear strain/% \| dynamic shear modulus/MPa \| dynamic shear strain/% \| dynamic shear modulus/MPa \| dynamic shear strain/% \| dynamic shear modulus/MPa \| \| 0.051 \| 98.84925 \| 0.0502 \| 82.42925 \| 0.114 \| 56.23448 \| 0.1545 \| 53.03971 \| 0.1039 \| 49.82682 \| 0.1717 \| 38.61392 \| \| 0.0712 \| 92.51576 \| 0.0713 \| 77.97576 \| 0.2032 \| 54.34099 \| 0.242 \| 46.70621 \| 0.1872 \| 43.99332 \| 0.3633 \| 35.28042 \| \| 0.091 \| 87.28219 \| 0.107 \| 73.45219 \| 0.3124 \| 51.46242 \| 0.35575 \| 45.47265 \| 0.3266 \| 38.25975 \| 0.576 \| 33.04685 \| \| 0.102 \| 85.25742 \| 0.1372 \| 69.83742 \| 0.4002 \| 50.64265 \| 0.492 \| 43.44788 \| 0.6735 \| 35.73499 \| 0.7266 \| 31.02209 \| \| 0.124 \| 84.3419 \| 0.1584 \| 67.1419 \| 0.4814 \| 49.33713 \| 0.60775 \| 43.53236 \| 0.8826 \| 34.31946 \| 0.8856 \| 30.10656 \| \| 0.149 \| 83.20076 \| 0.1792 \| 66.50076 \| 0.598 \| 48.44599 \| 0.75825 \| 43.39122 \| 1.0605 \| 33.67832 \| 1.0143 \| 30.96542 \| \| 0.165 \| 82.69131 \| 0.1938 \| 65.79131 \| 0.6458 \| 47.83653 \| 0.8155 \| 43.88176 \| 1.2174 \| 33.16886 \| 1.1511 \| 30.45597 \| \| 0.18 \| 81.38464 \| 0.2122 \| 65.28664 \| 0.7142 \| 46.43087 \| 0.8935 \| 43.5751 \| 1.3623 \| 32.8622 \| 1.272 \| 30.1493 \| \|  \|  \|  \|  \|  \|  \| 0.98325 \| 43.41098 \|  \|  \| 1.4184 \| 30.98519 \| \| **Perimeter pressure 80kPa** \| \| \| \| \| \| \| \| \| \| \| \| \| dynamic shear strain/% \| dynamic shear modulus/MPa \| dynamic shear strain/% \| dynamic shear modulus/MPa \| dynamic shear strain/% \| dynamic shear modulus/MPa \| dynamic shear strain/% \| dynamic shear modulus/MPa \| dynamic shear strain/% \| dynamic shear modulus/MPa \| dynamic shear strain/% \| dynamic shear modulus/MPa \| \| 0.0342 \| 124.65298 \| 0.0517 \| 96.14298 \| 0.063 \| 81.1261 \| 0.08825 \| 68.10921 \| 0.0678 \| 55.63054 \| 0.0881 \| 42.41764 \| \| 0.061 \| 110.84925 \| 0.0712 \| 91.42925 \| 0.109 \| 72.86737 \| 0.15925 \| 60.30548 \| 0.1039 \| 42.82682 \| 0.1717 \| 36.61392 \| \| 0.0871 \| 105.51576 \| 0.1084 \| 85.97576 \| 0.2162 \| 65.97388 \| 0.23275 \| 55.97199 \| 0.1872 \| 43.99332 \| 0.3633 \| 35.28042 \| \| 0.1091 \| 102.28219 \| 0.1519 \| 80.45219 \| 0.283 \| 63.0953 \| 0.4325 \| 52.73842 \| 0.3266 \| 38.25975 \| 0.576 \| 33.04685 \| \| 0.132 \| 98.25742 \| 0.1834 \| 76.83742 \| 0.4364 \| 62.27554 \| 0.57825 \| 48.71365 \| 0.6735 \| 38.73499 \| 0.7266 \| 34.02209 \| \| 0.154 \| 95.3419 \| 0.2183 \| 75.1419 \| 0.5222 \| 61.47001 \| 0.681 \| 46.79813 \| 1.08826 \| 36.31946 \| 0.8856 \| 34.10656 \| \| 0.1719 \| 93.20076 \| 0.2551 \| 74.50076 \| 0.6082 \| 61.57887 \| 0.8075 \| 45.65699 \| 1.2605 \| 37.67832 \| 1.0143 \| 32.96542 \| \| 0.20236 \| 92.69131 \| 0.2984 \| 73.79131 \| 0.747 \| 59.46943 \| 0.9075 \| 44.14754 \| 1.4174 \| 35.16886 \| 1.1511 \| 33.45597 \| \| 0.24252 \| 92.38464 \| 0.342 \| 73.28664 \| 0.813 \| 58.06376 \| 0.9825 \| 44.84087 \| 1.5623 \| 34.8622 \| 1.472 \| 32.1493 \| \|  \|  \|  \|  \|  \|  \| 1.0325 \| 43.89 \|  \|  \|  \|  \| \|  \|  \|  \|  \|  \|  \| 1.14 \| 43.87 \|  \|  \|  \|  \| \| **Perimeter pressure 120kPa** \| \| \| \| \| \| \| \| \| \| \| \| \| dynamic shear strain/% \| dynamic shear modulus/MPa \| dynamic shear strain/% \| dynamic shear modulus/MPa \| dynamic shear strain/% \| dynamic shear modulus/MPa \| dynamic shear strain/% \| dynamic shear modulus/MPa \| dynamic shear strain/% \| dynamic shear modulus/MPa \| dynamic shear strain/% \| dynamic shear modulus/MPa \| \| 0.065 \| 132.65298 \| 0.0567 \| 120.14298 \| 0.0698 \| 102.45337 \| 0.0651 \| 92.76376 \| 0.068 \| 85.49121 \| 0.071 \| 76.21866 \| \| 0.0912 \| 120.84925 \| 0.0813 \| 107.42925 \| 0.0801 \| 95.19464 \| 0.0813 \| 81.96004 \| 0.0758 \| 76.18748 \| 0.0978 \| 53.41493 \| \| 0.1157 \| 113.51576 \| 0.1134 \| 95.97576 \| 0.1242 \| 75.30115 \| 0.105 \| 70.62654 \| 0.083 \| 65.85398 \| 0.1411 \| 45.08143 \| \| 0.135 \| 108.28219 \| 0.155 \| 83.45219 \| 0.2179 \| 60.42258 \| 0.153 \| 60.39297 \| 0.1275 \| 53.62042 \| 0.192 \| 40.84787 \| \| 0.164 \| 99.25742 \| 0.1874 \| 75.83742 \| 0.2747 \| 57.10282 \| 0.222 \| 52.36821 \| 0.1971 \| 47.09566 \| 0.2717 \| 35.8231 \| \| 0.198 \| 92.3419 \| 0.2463 \| 70.1419 \| 0.3462 \| 54.29729 \| 0.306 \| 48.45268 \| 0.2606 \| 42.68013 \| 0.3652 \| 32.90758 \| \| 0.232 \| 88.20076 \| 0.2871 \| 68.50076 \| 0.3936 \| 52.90615 \| 0.3526 \| 46.31154 \| 0.349 \| 39.03899 \| 0.4381 \| 31.76644 \| \| 0.299 \| 82.69131 \| 0.333 \| 63.79131 \| 0.431 \| 51.2967 \| 0.4287 \| 44.80209 \| 0.4704 \| 36.52954 \| 0.5537 \| 30.25699 \| \| 0.332 \| 80.38464 \| 0.372 \| 64.28664 \| 0.526 \| 49.39103 \| 0.53 \| 42.49542 \| 0.5287 \| 34.72287 \| 0.6274 \| 28.95032 \| \|  \|  \|  \|  \|  \|  \| 0.6327 \| 40.33131 \|  \|  \| 0.6631 \| 27.7862 \|   Table. 22 Dynamic shear modulus-dynamic shear strain fitting parameters and model error assessment results under dry and wet cycling \| \| \| \| \| \| \| \| \| \| \| Pressurization/KPa \| Number of wet and dry cycles/N \| \| a \| b \| R^2^ \| MAE \| RMSE \| \| 40 \| 0 \| \| 0.01696 \| 0.17528 \| 0.944 \| 1.651 \| 1.856 \| \| 1 \| \| 0.02017 \| 0.17209 \| 0.996 \| 0.376 \| 0.475 \| \| 2 \| \| 0.02214 \| 0.11681 \| 0.965 \| 0.630 \| 0.883 \| \| 3 \| \| 0.0242 \| 0.13482 \| 0.901 \| 1.638 \| 1.894 \| \| 4 \| \| 0.02984 \| 0.17704 \| 0.976 \| 0.920 \| 1.201 \| \| 5 \| \| 0.0326 \| 0.15471 \| 0.938 \| 1.006 \| 1.162 \| \| 80 \| 0 \| \| 0.0146 \| 0.18133 \| 0.980 \| 1.666 \| 2.056 \| \| 1 \| \| 0.01691 \| 0.16866 \| 0.977 \| 1.274 \| 1.531 \| \| 2 \| \| 0.01814 \| 0.14651 \| 0.937 \| 2.243 \| 2.624 \| \| 3 \| \| 0.02297 \| 0.20055 \| 0.978 \| 1.380 \| 1.783 \| \| 4 \| \| 0.02789 \| 0.15197 \| 0.854 \| 2.891 \| 3.658 \| \| 5 \| \| 0.03095 \| 0.13281 \| 0.855 \| 1.982 \| 2.369 \| \| 120 \| 0 \| \| 0.01808 \| 0.32739 \| 0.995 \| 1.303 \| 1.470 \| \| 1 \| \| 0.02308 \| 0.35741 \| 0.995 \| 1.272 \| 1.541 \| \| 2 \| \| 0.02845 \| 0.39891 \| 0.982 \| 2.553 \| 2.997 \| \| 3 \| \| 0.03393 \| 0.42423 \| 0.956 \| 3.694 \| 4.525 \| \| 4 \| \| 0.04525 \| 0.49337 \| 0.926 \| 4.633 \| 5.763 \| \| 5 \| \| 0.05319 \| 0.5288 \| 0.914 \| 4.827 \| 5.990 \| | | |

Table. 23 Parameter a, b fitting relationship equation

| Pressurization/KPa | Parameter a relational equation | R^2^ | Parameter b relational equation | R^2^ |
| --- | --- | --- | --- | --- |
| 40 | a=0.01502*e^(-N/-6.998)+0.0022 | 0.984 | b=0.148+0.0347*sin(pi*(N-3.5514)/0.024) | 0.989 |
| 80 | a=0.01223*e^(-N/-5.694)+0.0021 | 0.984 | b=0.0324+0.0347*sin(pi*(N+0.375)/1.425) | 0.894 |
| 120 | a=0.02218*e^(-N/-5.219)-0.0041 | 0.995 | b=0.31881+0.04115*N | 0.982 |

Note：N is the number of wet/dry cycles

Table. 24 Modified eigenmodel

| Pressurization/KPa | Modified eigenstructural model |
| --- | --- |
| 40 | $G_{d}=\frac{1}{{{（0.01502*e^(-N/-6.998)+0.0022）\gamma}_{d}}^{0.148+0.0347*sin(pi*(N-3.5514)/0.024)}}$ |
| 80 | $G_{d}=\frac{1}{（0.01223*e^(-N/-5.694)+0.0021）{\gamma_{d}}^{0.0324+0.0347*sin(pi*(N+0.375)/1.425)}}$ |
| 120 | $G_{d}=\frac{1}{（0.02218*e^(-N/-5.219)-0.0041{{）\gamma}_{d}}^{b=0.31881+0.04115*N}}$ |

Note：N is the number of wet/dry cycles

Table. 25 Levels of factors in orthogonal test

| Level | Pressurization/KPa | Consolidation ratio | Number of dry and wet cycles /N |
| --- | --- | --- | --- |
| 1 | 40 | 1 | 0 |
| 2 | 80 | 1.5 | 3 |
| 3 | 120 | 2 | 5 |

Table. 26 Table of orthogonal tests

| Test number | Pressurization/KPa | Consolidation ratio | Number of dry and wet cycles /N |
| --- | --- | --- | --- |
| 1 | 1 | 1 | 1 |
| 2 | 1 | 2 | 2 |
| 3 | 1 | 3 | 3 |
| 4 | 2 | 1 | 2 |
| 5 | 2 | 2 | 3 |
| 6 | 2 | 3 | 1 |
| 7 | 3 | 1 | 3 |
| 8 | 3 | 2 | 2 |
| 9 | 3 | 3 | 1 |

Table. 27 Orthogonal test results of cement:phosphogypsum:red clay=6:47:47 mixes

| Independent variable | Degrees of freedom | Mean square | F | P | $R^{2}$ |
| --- | --- | --- | --- | --- | --- |
| Consolidation ratio | 2 | 278.824 | 17.24 | 0.345 | 0.982 |
| Number of dry and wet cycles /N | 2 | 2665.79 | 19.696 | 0.0378* |  |
| Pressurization/KPa | 2 | 125.85 | 8.65 | 0.766 |  |

Note：* p<0.05 ** p<0.01

| 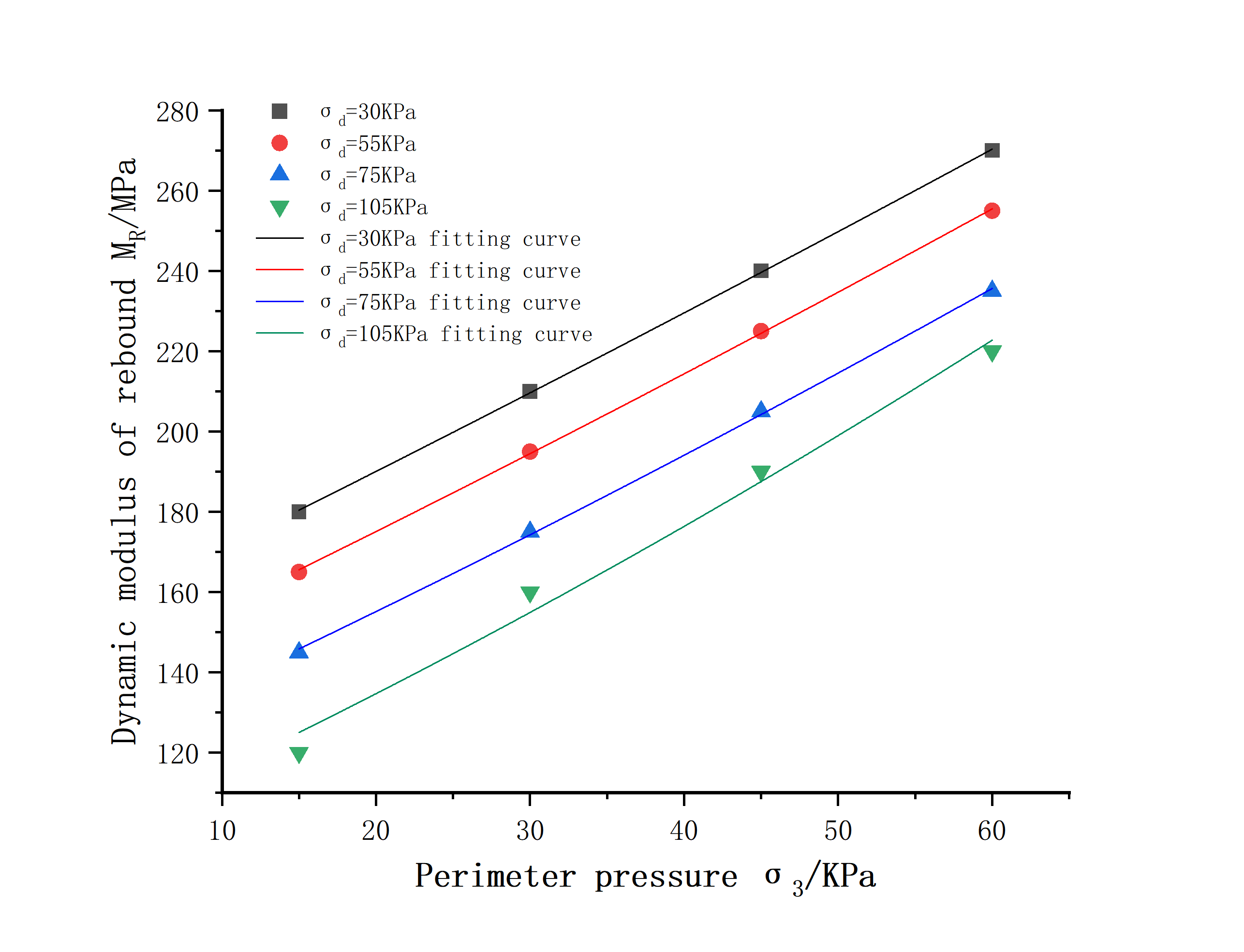 |
| --- |
| 0 wet/dry cycles |
| Fig. 11 Dynamic resilient modulus-perimeter pressure variation curves of cement:phosphogypsum:red clay=6:47:47 mixes under different bias stresses |

**Raw data for Figure 11**

| 0 wet/dry cycles | | | | |
| --- | --- | --- | --- | --- |
| Pressurization/KPa | Dynamic modulus of resilience/MPa | Dynamic modulus of resilience/MPa | Dynamic modulus of resilience/MPa | Dynamic modulus of resilience/MPa |
| 15 | 180 | 165 | 145 | 120 |
| 30 | 210 | 195 | 175 | 160 |
| 45 | 240 | 225 | 205 | 190 |
| 60 | 270 | 255 | 235 | 220 |

Table. 28 Dynamic modulus of rebound versus perimeter pressure curve fitting parameters and model error assessment results

| Bias stress/KPa | $k_{1}$ | $k_{2}$ | $k_{3}$ | R^2^ | MAE | RMSE |
| --- | --- | --- | --- | --- | --- | --- |
| 30 | 1.07966 | 1.22461 | 1.30455 | 0.998 | 0.148 | 0.370 |
| 55 | 0.94213 | 1.31303 | 0.86794 | 0.999 | 0.205 | 0.515 |
| 75 | 0.81852 | 1.45281 | 0.66941 | 0.998 | 0.297 | 0.745 |
| 105 | 0.67500 | 1.74847 | 0.51847 | 0.988 | 1.533 | 4.023 |

| 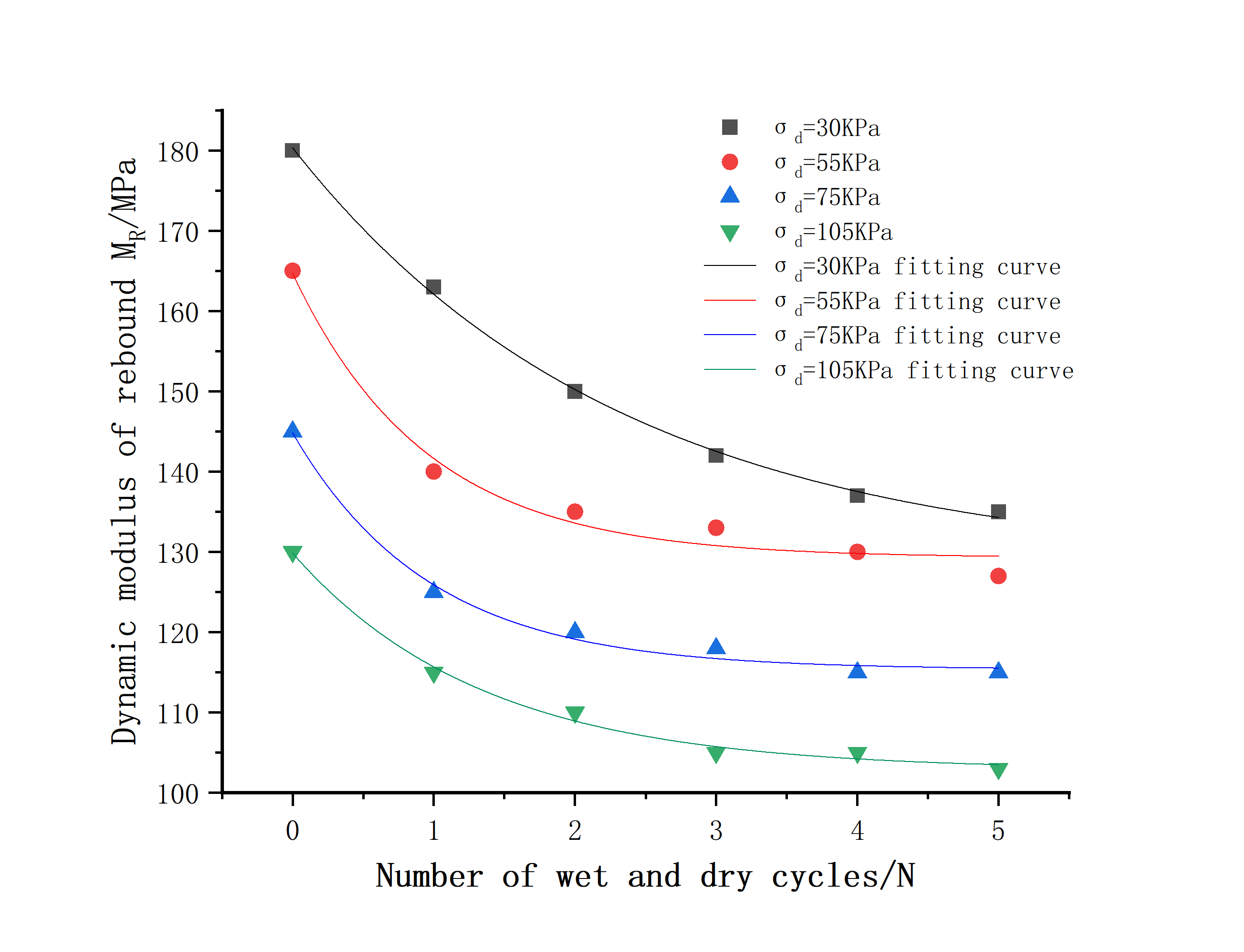 | | | | | | |  |
| --- | --- | --- | --- | --- | --- | --- | --- |
| Fig. 12 Variation curve of dynamic resilience modulus of cement:phosphogypsum:red clay=6:47:47 mix under bias stress - number of wet and dry cycles | | | | | | |  |
| **Raw data for Figure 12**   \| Number of wet and dry cycles/N \| Dynamic modulus of resilience/MPa \| Dynamic modulus of resilience/MPa \| Dynamic modulus of resilience/MPa \| Dynamic modulus of resilience/MPa \| \| --- \| --- \| --- \| --- \| --- \| \| 0 \| 180 \| 165 \| 145 \| 130 \| \| 1 \| 163 \| 140 \| 125 \| 115 \| \| 2 \| 150 \| 135 \| 120 \| 110 \| \| 3 \| 142 \| 133 \| 118 \| 105 \| \| 4 \| 137 \| 130 \| 115 \| 105 \| \| 5 \| 135 \| 127 \| 115 \| 103 \|   Table. 29 Results of curve fitting parameters and model error evaluation of dynamic modulus of resilience versus number of wet and dry cycles | | | | | | |  |
| Bias stress/KPa | $k_{1}$ | $k_{2}$ | $k_{3}$ | R^2^ | MAE | RMSE | |
| 30 | 128.268 | 1.16213 | 2.31662 | 0.998 | 0.544 | 0.589 | |
| 55 | 129.291 | 0.88999 | 0.94956 | 0.983 | 1.370 | 1.621 | |
| 75 | 115.375 | 0.7835 | 0.97228 | 0.993 | 0.770 | 0.842 | |
| 105 | 102.866 | 0.70812 | 1.34242 | 0.994 | 0.651 | 0.708 | |

Table. 30 Levels of factors in orthogonal test

| Level | Pressurization/KPa | Bias stress/KPa | Number of dry and wet cycles /N |
| --- | --- | --- | --- |
| 1 | 15 | 30 | 0 |
| 2 | 30 | 55 | 3 |
| 3 | 45 | 75 | 5 |

Table. 31 Table of orthogonal tests

| Test number | Pressurization/KPa | Bias stress/KPa | Number of dry and wet cycles /N | Dynamic modulus of resilience/Mpa |
| --- | --- | --- | --- | --- |
| 1 | 1 | 1 | 1 | 180 |
| 2 | 1 | 2 | 2 | 145 |
| 3 | 1 | 3 | 3 | 115 |
| 4 | 2 | 1 | 2 | 170 |
| 5 | 2 | 2 | 3 | 155 |
| 6 | 2 | 3 | 1 | 175 |
| 7 | 3 | 1 | 3 | 190 |
| 8 | 3 | 2 | 2 | 175 |
| 9 | 3 | 3 | 1 | 205 |

Table. 32 Orthogonal test results of cement:phosphogypsum:red clay=6:47:47 mixes

| Independent variable | Degrees of freedom | Mean square | F | p | R^2^ |
| --- | --- | --- | --- | --- | --- |
| Pressurization/KPa | 2 | 1411.11 | 19.844 | 0.048* | 0.975 |
| Bias stress/KPa | 2 | 442.778 | 6.227 | 0.138 |  |
| Number of dry and wet cycles /N | 2 | 951.11 | 13.375 | 0.070 |  |

Note：*P<0.05**P<0.01


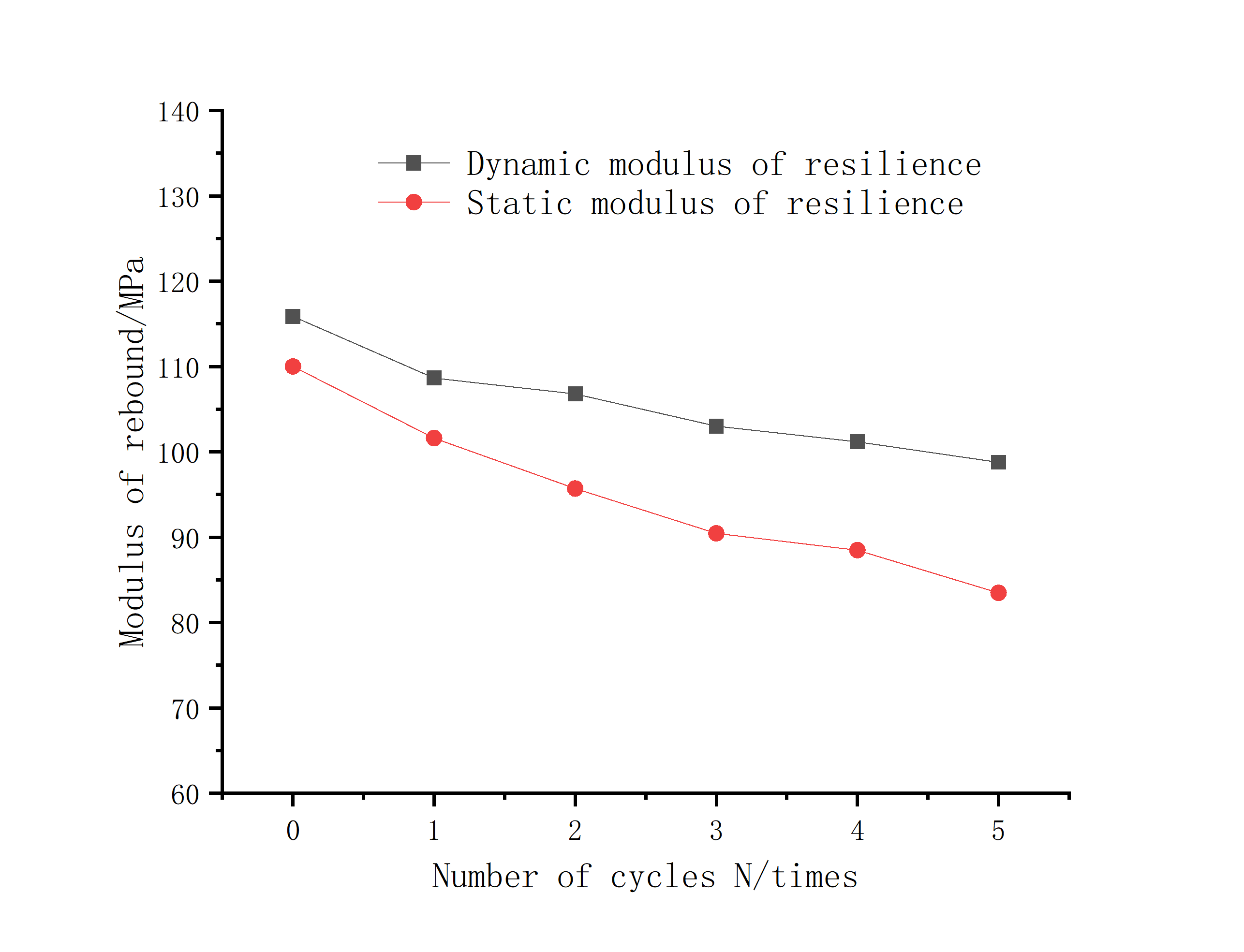

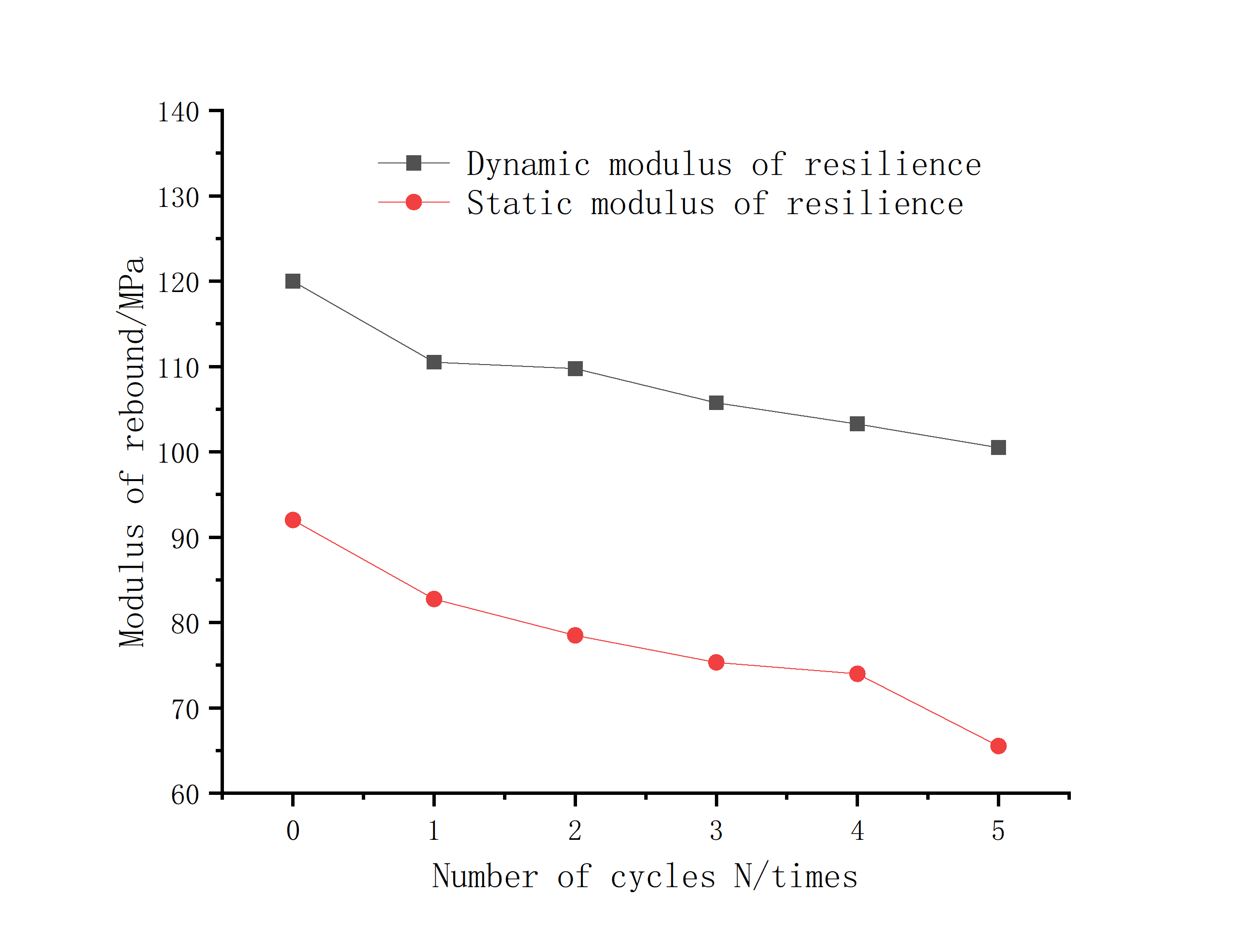

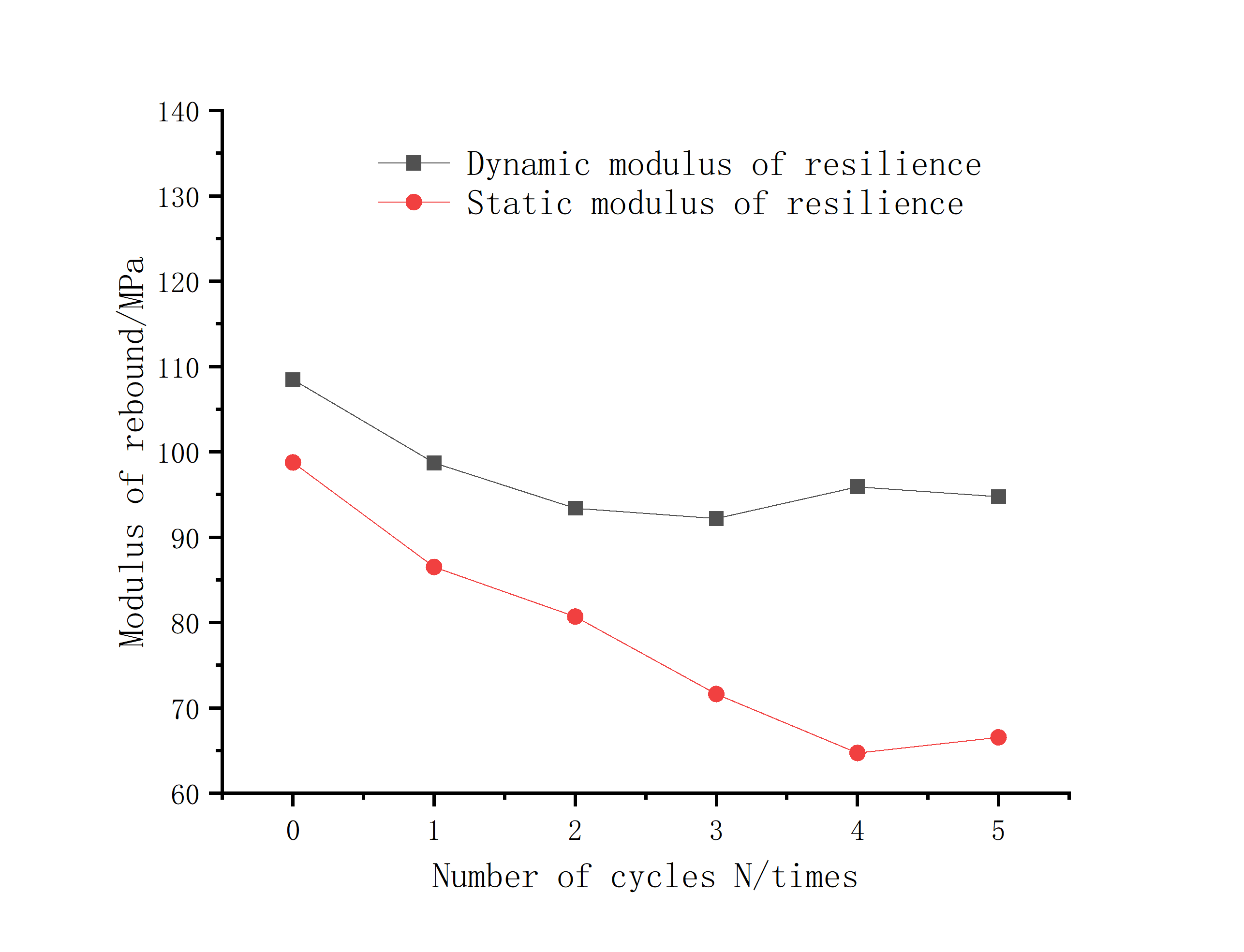


1. **(b ) (c)**

**Note: (a)Cement:Phosphogypsum:Red Clay = 6: 63: 31 (b)Cement:Phosphogypsum:Red Clay = 6:47:47 (c)Cement:Phosphogypsum:Red Clay = 6:31:63**

Fig. 13 Representative values of dynamic resilience modulus of mixes with different mix ratios-dry and wet cycle variation curves

**Raw data for Figure 13**

| **Cement:Phosphogypsum:Red Clay = 6: 63: 31** | | | **Cement:Phosphogypsum:Red Clay =6:47:47** | | |
| --- | --- | --- | --- | --- | --- |
|  | Dynamic modulus of resilience/MPa | Static modulus of resilience |  | Dynamic modulus of resilience/MPa | Static modulus of resilience |
| 0 | 115.88 | 110 | 0 | 108.46 | 98.76 |
| 1 | 108.64 | 101.6 | 1 | 98.7 | 86.5 |
| 2 | 106.8 | 95.7 | 2 | 93.4 | 80.7 |
| 3 | 103 | 90.44 | 3 | 92.2 | 71.62 |
| 4 | 101.17 | 88.47 | 4 | 95.9 | 64.7 |
| 5 | 98.78 | 83.48 | 5 | 94.75 | 66.54 |

| **Cement:Phosphogypsum:Red Clay = 6:31:63** | | |
| --- | --- | --- |
|  | Dynamic modulus of resilience/MPa | Static modulus of resilience |
| 0 | 108.46 | 98.76 |
| 1 | 98.7 | 86.5 |
| 2 | 93.4 | 80.7 |
| 3 | 92.2 | 71.62 |
| 4 | 95.9 | 64.7 |
| 5 | 94.75 | 66.54 |

Table .33 Dynamic and static modulus of resilience values

| specifications | C:P:T=6:47:47 | | C:P:T=6:63:31 | | C:P:T=6:31:63 | |
| --- | --- | --- | --- | --- | --- | --- |
| Number of wet and dry cycles | dynamic value（MPa） | static value（MPa） | dynamic value（MPa） | static value（MPa） | dynamic value（MPa） | static value（MPa） |
| 0 | 120.000 | 92.000 | 115.880 | 110.000 | 108.460 | 98.760 |
| 1 | 110.500 | 82.740 | 108.640 | 101.600 | 98.700 | 86.500 |
| 2 | 109.750 | 78.500 | 106.800 | 95.700 | 93.400 | 80.700 |
| 3 | 105.750 | 75.320 | 103.000 | 90.440 | 92.200 | 71.620 |
| 4 | 103.250 | 74.000 | 101.170 | 88.470 | 95.900 | 64.700 |
| 5 | 100.500 | 65.530 | 98.780 | 83.480 | 94.750 | 66.540 |
| Average of last three | 103.17 | 71.62 | 100.98 | 87.46 | 94.28 | 67.62 |
| Dynamic/static | 1.44 | | 1.15 | | 1.39 | |
|  | 1.1-1.5 | | | | | |
